# Discovery of Undescribed Clerodane Diterpenoids with Antimicrobial Activity Isolated from the Roots of *Solidago gigantea* Ait

**DOI:** 10.3390/ijms26189187

**Published:** 2025-09-20

**Authors:** Márton Baglyas, Zoltán Bozsó, Ildikó Schwarczinger, Péter G. Ott, József Bakonyi, András Darcsi, Ágnes M. Móricz

**Affiliations:** 1 Plant Protection Institute, HUN-REN Centre for Agricultural Research, Fehérvári út 132–144, 1116 Budapest, Hungary; baglyas.marton@atk.hun-ren.hu (M.B.); bozso.zoltan@atk.hun-ren.hu (Z.B.); schwarczinger.ildiko@atk.hun-ren.hu (I.S.); ott.peter@atk.hun-ren.hu (P.G.O.); bakonyi.jozsef@atk.hun-ren.hu (J.B.); 2 Doctoral School, Semmelweis University, Üllői út 26, 1085 Budapest, Hungary; 3 Pharmaceutical Chemistry and Technology Department, National Center for Public Health and Pharmacy, Szabolcs utca 33, 1135 Budapest, Hungary; darcsi.andras@nngyk.gov.hu

**Keywords:** giant goldenrod, clerodane diterpene, thin-layer chromatography–direct bioautography, TLC–MS, bioassay-guided isolation, antimicrobial activity

## Abstract

Three previously undescribed clerodane diterpenoids, including two *cis*-clerodanes, solidagolactone IX (**1**) and solidagoic acid K (**2**), and one *trans*-clerodane, solidagodiol (**3**), along with two known *cis*-clerodane diterpenoids, (−)-(5*R*,8*R*,9*R*,10*S*)-15,16-epoxy*-ent*-*neo*-cleroda-3,13,14-trien-18-ol (**4**) and solidagoic acid J (**5**), were isolated and comprehensively characterized from the ethanolic and ethyl acetate root extract of *Solidago gigantea* Ait. (giant goldenrod). Compound **4** has previously been reported from the roots of this species, whereas compound **5** was identified from the leaves of *S. gigantea* but not from the roots. The bioassay-guided isolation involved thin-layer chromatography–direct bioautography (TLC–DB) with a *Bacillus subtilis* antibacterial assay, preparative flash column chromatography, and TLC–mass spectrometry (MS). The chemical structures of the isolated compounds (**1**–**5**) were elucidated through extensive in-depth spectroscopic and spectrometric analyses, including one- and two-dimensional nuclear magnetic resonance (NMR) spectroscopy, high-resolution tandem mass spectrometry (HRMS/MS), and attenuated total reflectance Fourier-transform infrared (ATR–FTIR) spectroscopy. Their antimicrobial activities were evaluated using *in vitro* microdilution assays against *B. subtilis* and different plant pathogens. Compound **3** was the most active against the tested Gram-positive strains, exerting particularly potent effects against *Clavibacter michiganensis* with a minimal inhibitory concentration (MIC) value of 5.1 µM as well as *B. subtilis* and *Curtobacterium flaccumfaciens* pv. *flaccumfaciens* (MIC 21 µM for both). Compound **4** also strongly inhibited the growth of *C. michiganensis* (MIC 6.3 µM). Compounds **2**, **4**, and **5** displayed moderate to weak activity against *B. subtilis* and *C. flaccumfaciens* pv. *flaccumfaciens* with MIC values ranging from 100 to 402 µM. *Rhodococcus fascians* bacteria were moderately inhibited by compounds **3** (MIC 41 µM) and **4** (MIC 201 µM). Bactericidal activity was observed for compound **3** against *C. michiganensis* with a minimal bactericidal concentration (MBC) value of 83 µM. Compounds **2** and **3** demonstrated weak antifungal activity against *Fusarium graminearum*. Our findings underscore the value of bioassay-guided approaches in discovering previously undescribed bioactive compounds.

## 1. Introduction

The genus *Solidago* L., commonly known as goldenrods, belongs to the family Asteraceae and consists of approximately 139 herbaceous perennial species [1] that are predominantly native to North America (including Mexico), with a few species also indigenous to South America, Asia, and Europe (*Solidago virgaurea* L.) [1,2,3]. *Solidago canadensis* L., *Solidago gigantea* Ait., and *Euthamia graminifolia* (Nutt.) Cass. (formerly known as *Solidago graminifolia* (L.) Salisb.) were introduced from North America to Europe in the 18th century for ornamental purposes due to their decorative flowers. However, *S. canadensis* and *S. gigantea* escaped cultivation by the mid-19th century, and their populations began to expand exponentially toward the end of the 19th century [4]. Nowadays, they have become widespread weeds throughout Europe, and since 2004, both species have been included on the European and Mediterranean Plant Protection Organization (EPPO) list of invasive alien plants [5].

**Figure 1:**
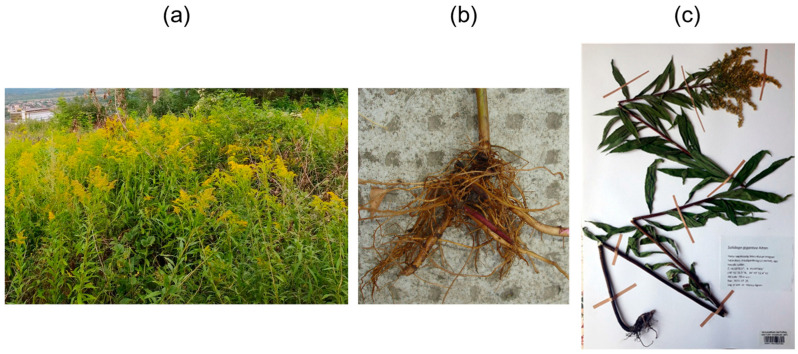
The widespread occurrence of invasive *Solidago gigantea* Ait. (giant goldenrod) in a field near Dorog, Hungary (**a**), the roots of *S. gigantea* (**b**), and the deposited voucher herbarium specimen (accession number: HNHM-TRA 00027284) (**c**).

*S. gigantea* Ait. (giant goldenrod) (Figure 1a–c) is an erect, rhizomatous, long-lived herb native to North America and is considered one of the most aggressive plant invaders in Europe. Owing to its vigorous vegetative growth and prolific seed production, it rapidly colonizes disturbed and semi-natural habitats, forming dense monocultures that suppress native vegetation, reduce biodiversity, and alter ecosystem functioning (Figure 1a) [6,7]. One of the strategies that contributes to its success as an invader is allelopathy, whereby *S. gigantea* releases allelochemicals into the soil that inhibit the germination and growth of neighboring plant species, which gives *S. gigantea* a competitive advantage in invaded habitats [8,9,10]. On the other hand, the aerial parts of *S. gigantea* have long been utilized in traditional folk medicine to treat urinary tract disorders, kidney and bladder inflammation, and microbial infections. These therapeutic uses are associated with its anti-inflammatory, antimicrobial, diuretic, and spasmolytic activities [11,12,13,14], demonstrating its versatile pharmacological potential. *Solidaginis herba*, containing the flowering and aerial parts of *S. gigantea*, *S. canadensis*, and *S. virgaurea*, is official in the European Pharmacopoeia [15]. The diverse biological effects of *S. gigantea* are attributed to a wide range of specialized metabolites, including flavonoids [16], phenolic acids [16], essential oil components, monoterpenoids [13], sesquiterpenoids [17], diterpenoids [18,19], and triterpenoids [20]. Antimicrobial compounds identified in the roots of goldenrods include polyacetylenes [21,22,23], clerodane diterpenoids [18,22,24,25,26,27], labdane diterpenoids [21], and essential oil terpenes [28]. Nevertheless, the antimicrobial constituents of *S. gigantea* roots remain underexplored.

We have recently detected eight clerodane diterpenoids with antimicrobial and/or acetylcholinesterase activity and isolated them from the roots of *S. gigantea* (Figure 1b) [18,19]. Encouraged by these findings, our ongoing search for bioactive constituents from *Solidago* species was continued [21,23,24,29,30,31,32], and further phytochemical investigations were undertaken to discover additional minor antimicrobial compounds from the roots of *S. gigantea*. Thus, this work aimed at non-targeted, effect-directed screening for antimicrobial compounds in the ethyl acetate and ethanol extracts of *S. gigantea* roots using TLC–direct bioautography (TLC–DB). This was followed by highly targeted bioassay-guided fractionation and isolation using preparative flash column chromatography and semi-preparative high-performance liquid chromatography (HPLC). The entire process was monitored by TLC hyphenations (ultraviolet (UV) and fluorescence detection (FLD), chemical derivatization with *p*-anisaldehyde–sulfuric acid or the vanillin–sulfuric acid reagent, DB using a *Bacillus subtilis* bioassay, and mass spectrometry (MS)). The structure elucidation of the isolated compounds was performed via comprehensive spectroscopic (nuclear magnetic resonance (NMR), attenuated total reflectance Fourier-transform infrared (ATR–FTIR), and UV spectroscopy), spectrometric techniques (flow injection analysis–high-resolution-heated electrospray ionization-tandem MS (FIA–HR-HESI-MS/MS)), and polarimetry. Moreover, the *in vitro* antibacterial, bactericidal, and antifungal activities of the isolated compounds were assessed against the non-pathogenic *B. subtilis* and the phytopathogenic *Curtobacterium flaccumfaciens* pv. *flaccumfaciens*, *Clavibacter michiganensis*, *Rhodococcus fascians*, *Pseudomonas syringae* pv. *tomato*, and *Xanthomonas arboricola* pv. *pruni*, as well as the plant pathogenic fungal strains *Fusarium graminearum* and *Bipolaris sorokiniana*.

## 2. Results and Discussion

### 2.1. Detection and Bioassay-Guided Isolation

**Figure 2:**
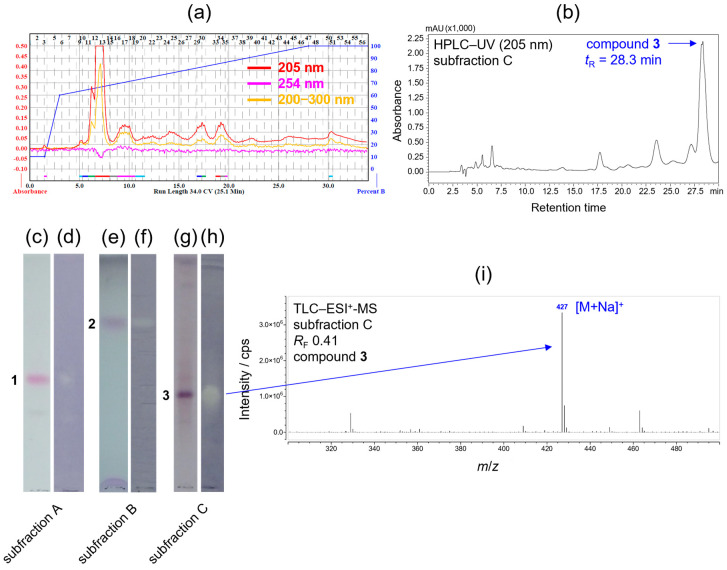
UV chromatograms at 205 nm (red), 254 nm (magenta), and 200–300 nm (yellow) (**a**) acquired by preparative reversed-phase (RP) flash column chromatographic separation of fraction 77–89 using a C_18_ column as a stationary phase with a gradient elution (blue). Semi-preparative RP-HPLC–UV chromatogram at 205 nm (**b**) recorded during the isolation of compound **3** (*t*_R_ = 28.3 min) from subfraction C (77–89/37–40) using a C_18_ column as a stationary phase with an isocratic elution. TLC chromatograms (**c**,**e**,**g**) visualized after derivatization with vanillin–sulfuric acid reagent (**c**,**e**) or *p*-anisaldehyde–sulfuric acid reagent (**g**), and TLC–DB bioautograms (**d**,**f**,**h**) obtained by a *B. subtilis* antibacterial assay of subfractions A (26–27/59–67/22–29), B ((26–27/32–39/23–26 + 26–27/40–48/39–67)/20–21), and C (77–89/37–40), containing compounds **1** (*R*_F_ 0.47), **2** (*R*_F_ 0.70), and **3** (*R*_F_ 0.41), respectively. TLC–ESI^+^-MS spectrum of compound **3** (**i**) recorded from subfraction C (77–89/37–40) at *R*_F_ 0.41 along with the assignment of the base peak (*m*/*z* 427 [M+Na]^+^).

In our preliminary investigations, the *n*-hexane, ethyl acetate, and ethanolic root extract of *S. gigantea* exhibited *in vitro* antibacterial activity against *B. subtilis*. In one of our recent works [18], eight high-abundance clerodane diterpenoids were detected, isolated, and identified from the ethanolic root extract of *S. gigantea* as the compounds potentially responsible for the observed antibacterial effect. Since our previous study primarily revealed the major constituents, the present work was undertaken to explore components occurring in lower abundance. For this purpose, an increased quantity of plant material (112 g from the collection of sample R2022 and 661 g from the collection of sample R2023) was subjected to extraction with ethanol and ethyl acetate, successive fractionation, purification, and isolation using preparative normal-phase (NP) and reversed-phase (RP) flash column chromatography (Figure 2a), and when necessary, semi-preparative RP-HPLC (Figure 2b), exploiting the orthogonal selectivity of the NP and RP stationary phases. Each separation step was monitored by TLC–UV/FLD, TLC–vanillin (Figure 2c,e and Figure S1) or TLC–*p*-anisaldehyde derivatization (Figure 2g and Figure S1), TLC–DB (Figure 2d,f,h, and Figure S1), TLC–MS (Figure 2i), and RP-HPLC–DAD-ESI-MS. As a result, five compounds were isolated, all of which exhibited antibacterial activity *in situ* in TLC–*B. subtilis* assay: compound **1** (1.3 mg, white amorphous solid), compound **2** (1.5 mg, white amorphous solid), compound **3** (2.0 mg, white amorphous solid), compound **4** (3.9 mg, pale-yellow oil), and compound **5** (2.7 mg, white amorphous solid).

Due to the wide polarity range of the isolated compounds, different TLC (silica gel) mobile phases were employed for their separation. TLC–DB with the *B. subtilis* antibacterial assay revealed inhibition zones for each isolated compound (Figure 2d,f,h and Figure S1), thereby demonstrating their *in situ* antibacterial activity.

### 2.2. Structure Elucidation

The structure elucidation was performed using extensive spectroscopic and spectrometric techniques as well as comparison with previously reported data. The novelty of the structures of compounds **1**–**3** was confirmed by searching in the CAS SciFinder^®^ and Reaxys databases. The recorded one- and two-dimensional NMR spectra (Figures S2–S9 for **1**, Figures S16–S23 for **2**, Figures S30–S36 for **3**, Figures S41–S47 for **4**, Figures S49–S55 for **5**), HR-HESI-MS(/MS) spectra (Figures S10–S13 for **1**, Figures S24–S27 for **2**, Figures S37–S38 for **3**, Figure S48 for **4**, Figures S56–S59 for **5**), UV spectra (Figure S14 for **1**, Figure S28 for **2**, Figure S39 for **3**), and ATR-FTIR spectra (Figure S15 for **1**, Figure S29 for **2**, Figure S40 for **3**) can be found in the Supplementary Materials.

**Figure 3:**
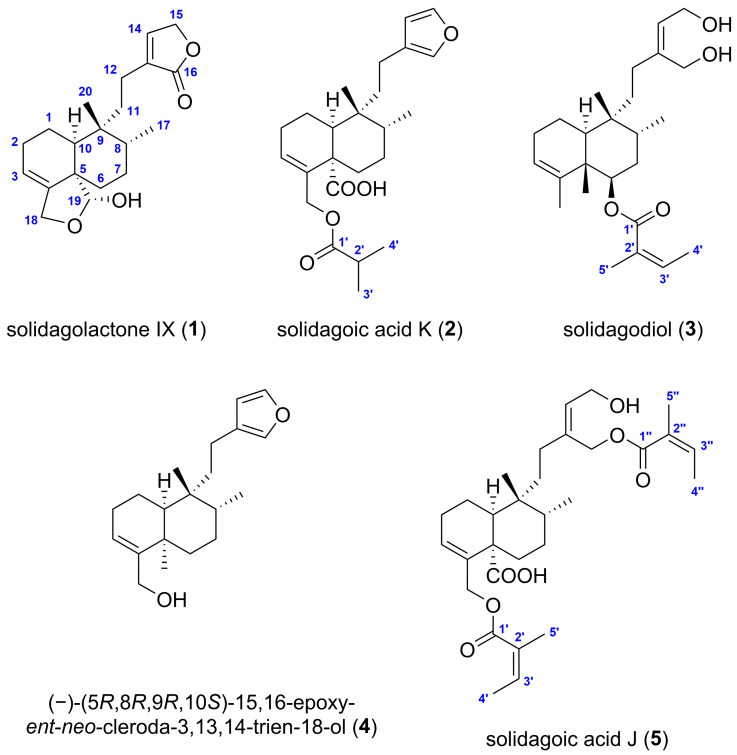
The chemical structures of the five isolated compounds (**1**–**5**) with atomic numbering (blue). Compounds **1** (solidagolactone IX, (−)-(5S*,8R*,9R*,10S*,19R*)-18,19-epoxy-19-hydroxy-cleroda-3,13-dien-16,15-olide), **2** (solidagoic acid K, (−)-(5S*,8R*,9R*,10S*)-15,16-epoxy-18-isobutyryloxy-cleroda-3,13,14-trien-19-oic acid), and **3** (solidagodiol, (−)-(5S*,6R*,8R*,9R*,10S*,13*Z*)-6-angeloyloxy-cleroda-3,13-dien-15,16-diol) are previously undescribed, whereas compounds **4** ((−)-(5*R*,8*R*,9*R*,10*S*)-15,16-epoxy*-ent*-*neo*-cleroda-3,13,14-trien-18-ol) and **5** (solidagoic acid J, (5S*,8R*,9R*,10S*,13*Z*)-16,18-diangeloyloxy-15-hydroxy-cleroda-3,13-dien-19-oic acid) are known clerodane diterpenoids.

Solidagolactone IX (**1**) (Figure 3) was obtained as a white amorphous solid with a specific optical rotation of [*α*]_D_^25^ −10.7 (*c* 0.075, CHCl_3_). Its molecular formula was established as C_20_H_28_O_4_ deduced from the ^13^C DEPTQ NMR spectrum and based on the positive *m*/*z* 355.1879 [M+Na]^+^ (calculated for C_20_H_28_O_4_Na^+^, *m*/*z* 355.1880 [M+Na]^+^, error: −0.3 ppm)) and negative ion mode HR-HESI-MS spectrum (*m*/*z* 331.1915 [M−H]^−^ (calculated for C_20_H_27_O_4_^−^, *m*/*z* 331.1915 [M−H]^−^, error: 0.1 ppm)), indicating 7 double bond equivalents (DBEs). Its ^1^H NMR spectrum (Table 1) exhibited proton resonances corresponding to two methyl groups at *δ*_H_ 0.97 (s, 3H, H_3_-20) and 0.81 (d, *J* = 6.9 Hz, 3H, H_3_-17), two vinylic hydrogens at *δ*_H_ 7.17 (br s, 1H, H-14) and 5.59 (m, 1H, H-3), two oxymethylene groups at *δ*_H_ 4.79 (br s, 2H, H_2_-15) and 4.35 (m, 2H, H_2_-18), an oxymethine group at *δ*_H_ 5.52 (d, *J* = 5.7 Hz, 1H, H-19), and a hydroxy group at *δ*_H_ 5.06 (d, *J* = 5.7 Hz, 1H, 19-OH). Based on the ^13^C DEPTQ (Table 1), ^1^H–^13^C multiplicity-edited HSQC (edHSQC) and ^1^H–^13^C HMBC spectroscopic data, the twenty ^13^C resonances were assigned to two methyl groups (*δ*_C_ 26.4, 15.9), six aliphatic (*δ*_C_ 32.1, 30.4, 28.1, 26.3, 20.6, 20.1) and two oxygenated methylene groups (*δ*_C_ 70.6, 67.2), two olefinic (*δ*_C_ 146.0, 118.3), two aliphatic (*δ*_C_ 38.6, 37.2) and one dioxygenated (*δ*_C_ 100.8) methine groups, and five non-hydrogenated carbons, including two olefinic (*δ*_C_ 142.1, 135.8), two aliphatic (*δ*_C_ 49.5, 38.2) and one ester (*δ*_C_ 176.1) carbons. The presence of the hydroxy group was supported by the characteristic broad IR absorption band at 3398 cm^–1^. Therefore, the structure of compound **1** contains two trisubstituted carbon–carbon double bonds and an ester group, indicating the presence of four rings to account for the required number of DBEs.

**Figure 4:**
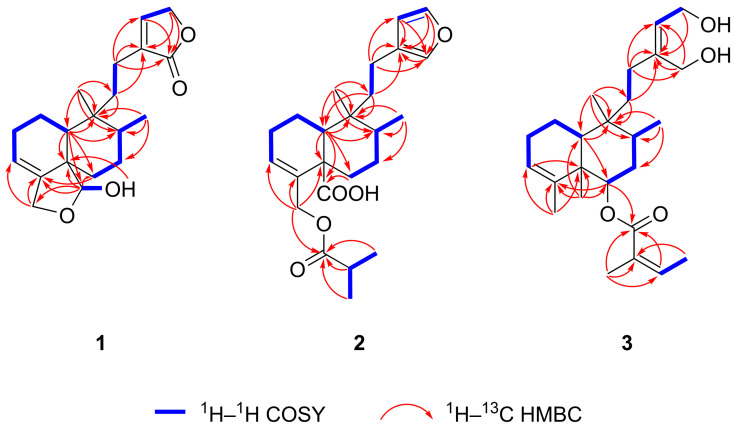
Selected ^1^H–^1^H COSY (blue) and ^1^H–^13^C HMBC (red) correlations of the three previously undescribed compounds (**1**–**3**).

^1^H–^1^H COSY and ^1^H–^1^H TOCSY spectra of compound **1** revealed five distinct spin systems: H-10/H_2_-1a/H_2_-1b/H_2_-2/H-3, H_2_-6a/H_2_-6b/H_2_-7/H-8/H_3_-17, H_2_-11a/H_2_-11b/H_2_-12a/H_2_-12b, H-14/H_2_-15, and H-19/19-OH (Figure 4). A 6/6 fused A/B ring system (C-1–C-10), bearing two methyl groups (C-17, C-20), an oxygenated methylene (C-18), and an oxygenated methine (C-19) group, was established based on the spin systems H-10/H_2_-1a/H_2_-1b/H_2_-2/H-3 and H_2_-6a/H_2_-6b/H_2_-7/H-8/H_3_-17, along with the ^1^H–^13^C HMBC correlations from H-10 to C-5, C-6, C-8, C-9, C-19, from H_3_-17 to C-7, C-8, C-9, from H_2_-18 to C-3, C-4, and from H_3_-20 to C-8, C-9, C-10 (Figure 4). A five-membered hemiacetal ring (*γ*-lactol) was constructed between C-18 and C-19, with the methylene group at C-18, from the spin system H-19/19-OH and from HMBC correlations from H-10 to C-19, H_2_-18 to C-3, C-4, from H-19 to C-4, C-5, C-6, C-18, and from 19-OH to C-5. The presence of a five-membered, *α*,*β*-unsaturated lactone moiety (*γ*-lactone, butenolide) substituted at C-13 (α position of the butenolide ring) was inferred from the spin system H-14/H_2_-15 and from the HMBC correlations from H_2_-11 to C-13, H_2_-12 to C-13, C-14, C-16, from H_2_-14 to C-16, and from H_2_-15 to C-13, C-16. The lactone unit is attached to C-9 via an ethylene bridge, which comprises C-11 and C-12 as deduced from the spin system H_2_-11a/H_2_-11b/H_2_-12a/H_2_-12b and from HMBC correlations from H_2_-11 to C-9, C-13, from H_2_-12 to C-13, and from H_3_-20 to C-11. These NMR spectroscopic data confirmed that compound **1** has a clerodane-type diterpene skeleton with a five-membered cyclic hemiacetal, and a side chain (C-11–C-16) featuring a butenolide ring.

**Figure 5:**
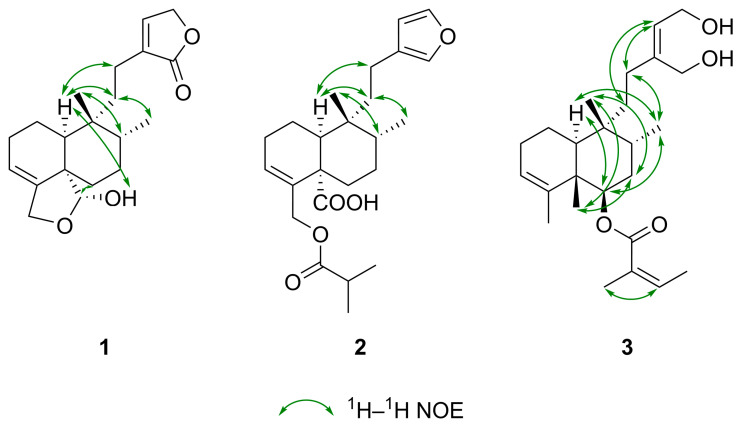
Key ^1^H–^1^H NOE correlations (green) observed in the ^1^H–^1^H ROESY spectra of the three previously undescribed compounds (**1**–**3**).

After determining the two-dimensional (2D) structure, the relative configuration of compound **1** was elucidated by diagnostic ^1^H–^1^H spin-spin coupling constants, ^1^H–^1^H NOE correlations observed in the ^1^H–^1^H ROESY spectrum, and ^13^C NMR-based empirical rules. A ^13^C NMR chemical shift difference of 10.5 ppm observed between C-17 and C-20 is indicative of a *cis*-fused A/B ring junction and concurrently implies a *trans* orientation between the C-17 and C-20 methyl groups [33,34], consistent with a *cis*-*trans* (CT)-type clerodane skeleton. The downfield ^13^C NMR chemical shift of C-20 (*δ*_C_ 27.0) supports this assignment, falling within the *δ*_C_ 21–29 range of *cis*-clerodanes, in contrast to *δ*_C_ 17–19, typical of *trans*-clerodanes [35,36]. Additionally, the ^1^H NMR chemical shift of H_3_-20 (*δ*_H_ 0.97) downfield relative to H_3_-17 (*δ*_H_ 0.81) further corroborates the *cis* A/B ring fusion [34]. An α-axial orientation for H-10 was indicated by its ^1^H–^1^H coupling constants of ^3^*J*_H-10, H-1ax_ = 12.1 Hz (axial–axial coupling) and ^3^*J*_H-10, H-1eq_ = 3.6 Hz (axial–equatorial coupling). The suggested CT-type clerodane diterpene framework with a nonsteroidal conformation and the α-position of 19-OH group and the *β*-orientation of H-19 was confirmed by NOE enhancements between H-10/H_2_-11a, H-10/H_2_-12a, H_3_-17/H_2_-11b, H_3_-17/H_3_-20, H-19/H_2_-6a, H-19/H_2_-7, 19-OH/H-10, H_3_-20/H-8, H_3_-20/H-10 (Figure 5), demonstrating a *cis* fusion of A and B rings [33]. The five-membered hemiacetal ring with the methylene at C-18 was further verified by the NOE cross-peak between H-3/H_2_-18. Consequently, the structure of compound **1**, a previously undescribed *cis*-clerodane diterpenoid, was established to be (−)-(5S*,8R*,9R*,10S*,19R*)-18,19-epoxy-19-hydroxy-cleroda-3,13-dien-16,15-olide, trivially named solidagolactone IX.

Solidagoic acid K (**2**) (Figure 3) was obtained as a white amorphous solid with a specific optical rotation of [*α*]_D_^25^ −25.3 (*c* 0.095, CHCl_3_). Its molecular formula was established to be C_24_H_34_O_5_, determined from the ^13^C DEPTQ NMR and positive (*m*/*z* 425.2298 [M+Na]^+^ (calculated for C_24_H_34_O_5_Na^+^, *m*/*z* 425.2298 [M+Na]^+^, error: −0.1 ppm)) and negative ion mode (*m*/*z* 401.2329 [M−H]^−^ (calculated for C_24_H_33_O_5_^−^, *m*/*z* 401.2329 [M−H]^−^, error: 0.1 ppm)) HR-HESI-MS spectra, demanding 5 DBEs. Comparison of the ^1^H, ^13^C, and 2D NMR spectroscopic data of compound **2** (Table 1) with those of compound **1** indicated the presence of the same 6/6 fused A/B ring system bearing two methyl groups. However, its molecular formula revealed four additional carbon atoms compared to **1**, which was attributed to the butyryloxy group. This assignment was evidenced by the ^1^H NMR resonances at *δ*_H_ 2.54 (sept, *J* = 7.0 Hz, 1H, H-2′), 1.15 (d, *J* = 7.0 Hz, 6H, H_3_-3′ and H_3_-4′), the ^13^C NMR resonances *δ*_C_ 176.9 (C, C-1′), 34.2 (CH, C-2′), 19.10 (CH_3_, C-3′/C-4′), 19.06 (CH_3_, C-3′/C-4′), the spin system H-2′/H_3_-3′/H_3_-4′ (Figure 4) identified from ^1^H–^1^H COSY and ^1^H–^1^H TOCSY spectra, and the HMBC correlations from H-2′ to C-1′, from H_3_-3′ to C-1′, and from H_3_-4′ to C-1′ (Figure 4). The attachment of the butyryloxy moiety at C-18 was clarified by the HMBC correlation from H_2_-18 to C-1′. The presence of a butyryloxy moiety was further corroborated by the MS/MS fragmentation pattern of the precursor ion at *m*/*z* 425.2298 [M+Na]^+^ to give fragment ions at *m*/*z* 337.1774 [M+Na−C_4_H_8_O_2_]^+^ and *m*/*z* 111.0415 (C_4_H_8_O_2_^+^). Additionally, the MS/MS fragmentation of the precursor ion at *m*/*z* 401.2329 [M−H]^−^ yielded a fragment ion at *m*/*z* 87.0451 (C_4_H_7_O_2_^−^), corresponding to an isobutyrate ion (C_4_H_7_O_2_^−^). A carboxylic group was identified by the ^13^C resonance at *δ*_C_ 179.5 (C, C-19) and was assigned to C-19, supported by the HMBC correlations from H_2_-6 to C-19 and H-10 to C-19, indicating the replacement of the five-membered hemiacetal in **1**. Further evidence was provided for the presence of a COOH group by the MS/MS fragmentation of the precursor ion at *m*/*z* 401.2329 [M−H]^−^, which resulted in the neutral loss of CO_2_, yielding a fragment ion at *m*/*z* 357.2432 [M−H−CO_2_]^−^. Another dissimilarity between compounds **2** and **1** was the side chain unit, which comprised C-11–C-16. The *α*,*β*-unsaturated lactone moiety was replaced by a *β*-substituted furan ring based on the ^1^H signals at *δ*_H_ 7.30 (t, *J* = 1.7 Hz, 1H, H-15), 7.15 (t, *J* = 1.5 Hz, 1H, H-16), 6.25 (dd, *J* = 1.8, 0.9 Hz, 1H, H-14), the ^13^C NMR resonances at *δ*_C_ 142.6 (CH, C-15), 138.6 (CH, C-16), 126.1 (C, C-13), 111.3 (CH, C-14), the spin system H-14/H-15 observed in the ^1^H–^1^H COSY spectrum, and the HMBC correlations from H-14 to C-13, C-16, from H-15 to C-13, C-16, and from H-16 to C-13. The *β*-substituted furan is connected to C-9 via an ethylene bridge, which comprises C-11 and C-12 (3-ethylfuran unit) as deduced from the spin system H_2_-11a/H_2_-11b/H_2_-12a/H_2_-12b and from HMBC correlations from H_2_-11 to C-9, C-13, and from H_2_-12 to C-13, C-14, and C-16. The relative configuration of compound **2** was elucidated to be the same (CT-type clerodane diterpene) as that of **1** by comparing their NOE enhancements (Figure 5) and ^1^H–^1^H coupling constants. This assignment was further supported by the ^1^H NMR chemical shift relationship between H_3_-17 (*δ*_H_ 0.85) and H_3_-20 (*δ*_H_ 0.98), with H_3_-20 being downfield than H_3_-17 [34], and by the ^13^C NMR chemical shift difference of 11.1 ppm between C-17 (*δ*_C_ 15.8) and C-20 (*δ*_C_ 26.9), which exceeds the empirical threshold of 10 ppm [33,34]. Thus, compound **2**, a previously undescribed *cis*-clerodane furanoditerpenoid acid, was assigned as (−)-(5S*,8R*,9R*,10S*)-15,16-epoxy-18-isobutyryloxy-cleroda-3,13,14-trien-19-oic acid, trivially named solidagoic acid K.

Compound **3** (Figure 3) was obtained as a white amorphous solid with a specific optical rotation of [*α*]_D_^25^ −9.1 (*c* 0.11, CHCl_3_). It gave a molecular formula of C_25_H_40_O_4_ based on the ^13^C DEPTQ NMR data and the positive-ion HR-HESI-MS signal at *m*/*z* 427.2819 [M+Na]^+^ (calculated for C_25_H_40_O_4_Na^+^, *m*/*z* 427.2818 [M+Na]^+^, error: −0.3 ppm). The ^1^H, ^13^C, and 2D NMR spectroscopic data (Table 1) showed that compound **3** shared the same 6/6 fused A/B ring system as compounds **1** and **2**. However, besides the methyl groups attached at C-8 and C-9, two additional methyl groups are linked to the bicyclic ring system at C-4 (*δ*_H_ 1.56 (ov., 3H, H_3_-18), *δ*_C_ 20.7/20.8 (CH_3_, C-18)) and C-5 (*δ*_H_ 1.20 (s, 3H, H_3_-19), *δ*_C_ 17.1 (CH_3_, C-19)) as in clerodane, supported by the HMBC correlations from H_3_-18 to C-3, C-4, C-5 and from H_3_-19 to C-4, C-5, C-6, C-10 (Figure 4). A different acyloxy group was present in comparison to compound **2.** The molecular formula of compound **3** indicated 25 carbon atoms, with five additional carbons compared to the clerodane skeleton, which were assigned to an angeloyloxy moiety. It was identified by the ^1^H resonances at *δ*_H_ 6.04 (qq, *J* = 7.3, 1.3 Hz, 1H, H-3′), 2.00 (dq, *J* = 7.3, 1.6 Hz, 3H, H_3_-4′), 1.89 (p, *J* = 1.5 Hz, 3H, H_3_-5′), and by the five ^13^C NMR signals at *δ*_C_ 167.5 (C, C-1′), 137.8 (CH, C-3′), 128.7 (C, C-2′), 20.8/20.7 (CH_3_, C-5′), and 15.8 (CH_3_, C-4′), confirmed by the spin system H-3′/H_3_-4′ (Figure 4) along with the HMBC correlations from H-3′ to C-1′, from H_3_-4′ to C-2′, and from H_3_-5′ to C-1′, C-2′, C-3′. The presence of an angeloyloxy moiety was further proved by the MS/MS fragmentation of the precursor ion at *m*/*z* 427.2815 [M+Na]^+^, which resulted in the formation of the fragment ion at *m*/*z* 327.2293 [M+Na−C_5_H_8_O_2_]^+^, corresponding to a neutral loss of an angeloyloxy (C_5_H_8_O_2_) group. In contrast to compound **2**, the acyloxy group is located at C-6 of the clerodane skeleton rather than at C-18, as indicated by the downfield ^1^H and ^13^C NMR chemical shifts of H-6 (*δ*_H_ 5.09) and C-6 (*δ*_C_ 74.2). This assignment was confirmed by the HMBC correlation from H-6 to C-1′. Another structural difference compared to compounds **1** and **2** was observed in the side chain unit, which comprised C-11–C-16. Instead of lactone and furan rings, a but-2-ene-1,4-diol moiety was constructed based on the ^1^H NMR signals at *δ*_H_ 5.64 (t, *J* = 6.9 Hz, 1H, H-14), 4.22 (dd, *J* = 6.9, 2.6 Hz, 1H, H_2_-15), 4.18 (d, *J* = 2.0 Hz, 1H, H_2_-16), the ^13^C NMR resonances at *δ*_C_ 144.9 (C, C-13), 126.4 (CH, C-14), 61.2 (CH_2_, C-16), 58.8 (CH_2_, C-15), the COSY correlation between H-14/H_2_-15, and the HMBC correlations from H-14 to C-13, from H_2_-15 to C-13, and from H_2_-16 to C-13, C-14. The characteristic broad IR absorption band at 3357 cm^–1^ confirmed the presence of the hydroxy groups. The but-2-ene-1,4-diol moiety is attached to C-9 via an ethylene bridge, which comprises C-11 and C-12, supported by the spin system H_2_-11a/H_2_-11b/H_2_-12a/H_2_-12b and HMBC correlations from H_2_-11 to C-9, C-13, and from H_2_-12 to C-13, C-14, and C-16. Regarding the relative configuration of compound **3**, the presence of an angeloyloxy rather than an isomeric tigloyloxy moiety was suggested by the ^1^H NMR chemical shift of H-3′ (*δ*_H_ 6.04), which closely matches the reported value for methyl angelate (*δ*_H_ 6.06) and differs substantially from that of methyl tiglate (*δ*_H_ 6.90) [37]. The NOE interaction between H-3′/H_3_-5′, along with the absence of NOE correlation between H_3_-4′/H_3_-5′ (Figure 5), confirmed the *Z* configuration of the double bond, thereby establishing the substituent as an angeloyloxy group. The angeloyloxy group linked to C-6 was placed at the *β*-equatorial orientation, and appropriately, the H-6 proton was located at the α-axial position based on the ^1^H–^1^H coupling constants of ^3^*J*_H-6, H-7ax_ = 11.1 Hz (axial–axial coupling) and ^3^*J*_H-6, H-7eq_ = 4.6 Hz (axial–equatorial coupling). Starting from the α-oriented H-6 proton, according to the NOE correlations between H-6/H-10, H-6/H_3_-17, H-10/H_3_-17, and H_3_-17/H_2_-12, H-6, H-10, H_3_-17, and the side chain (C-10–C-16) were assigned as α and simultaneously H_3_-19, H_3_-20, and the angeloyloxy group as *β*, supported by the NOE interactions between H_2_-7a/H_3_-19, H_2_-7a/H_3_-20, H_3_-19/H_3_-20. The established TT-type clerodane diterpene skeleton was corroborated by the ^13^C NMR chemical shift difference of 5.2 ppm between C-17 (*δ*_C_ 15.2) and C-20 (*δ*_C_ 20.4) [33,38] and the ^1^H NMR chemical shift of H_3_-20 (*δ*_H_ 0.97) upfield from H_3_-17 (*δ*_H_ 1.08) [34]. The ROESY spectrum displayed cross-peaks between H-14/H_2_-11 and H-14/H_2_-12, although no correlation was observed between H-14/H_2_-16, which is consistent with a double bond of *Z* configuration. Compound **3**, a previously undescribed *trans*-clerodane diterpenoid, was consequently elucidated as (−)-(5S*,6R*,8R*,9R*,10S*,13*Z*)-6-angeloyloxy-cleroda-3,13-dien-15,16-diol, trivially named solidagodiol.

In addition, two known *cis*-clerodane diterpenoids, (−)-(5*R*,8*R*,9*R*,10*S*)-15,16-epoxy*-ent*-*neo*-cleroda-3,13,14-trien-18-ol (**4**) [18,26] (Figure 3) and solidagoic acid J ((5S*,8R*,9R*,10S*,13*Z*)-16,18-diangeloyloxy-15-hydroxy-cleroda-3,13-dien-19-oic acid) (**5**) [29] (Figure 3), were identified by comparing their NMR and HRMS(/MS) data with those reported in the literature and by matching RP-HPLC retention times and TLC *R*_F_ values with previously isolated compounds.

Clerodane diterpenoids are a large and widespread class of specialized metabolites, with over 1300 compounds isolated from various natural sources, including plants, fungi, bacteria, and marine animals, accounting for the majority of diterpenoids [39,40]. These natural products have attracted considerable attention in the past decade due to their diverse and valuable biological activities, such as *α*-glucosidase inhibitory [41], allelopathic [22], analgesic [42], antiadipogenic [43], antibacterial [44], antidiabetic [45], anti-inflammatory [44], antifeedant [46], antifungal [47], antihypertensive [48], antileishmanial [49], antiproliferative [45], antiviral [50], cardioprotective [51], cytotoxic [52], immunomodulatory [53], immunosuppressant [54], insecticidal [55], neuroprotective [56], and phytotoxic [57] properties. Clerodane diterpenoids are classified into two major groups—*cis* and *trans*—according to the relative configuration of the 6/6 fused A/B ring junction, with approximately 75% being *trans* and 25% *cis* [39]. The basic clerodane skeleton is further divided into four types—*cis*-*cis* (CC), *cis*-*trans* (CT), *trans*-*cis* (TC), and *trans*-*trans* (TT)—based on the configuration at the A/B ring fusion (H_3_-19/H-10) and the relationship between the methyl groups attached to C-8 and C-9 (H_3_-17/H_3_-20) (Figure 3) [58]. As predicted from their biosynthetic pathways, a *cis* orientation between the methyl groups H_3_-17 and H_3_-20 has been described in most clerodanes [58]. Compounds **1**, **2**, **4**, and **5** belong to the most rarely occurring CT-type class, whereas compound **3** is classified as a member of the TT-type group (Figure 3). The co-occurrence of *cis*- and *trans*-clerodane diterpenoids within the same plant species is noteworthy, though not unprecedented, as both CT-type and TC-type clerodanes have also been reported in *Solidago altissima* [33]. Compounds **1**–**3** are previously undescribed clerodane diterpenoids that have not yet been isolated from natural sources or synthesized. Compound **4** was isolated from the roots of *S. gigantea* [18,26]. Solidagoic acid J (**5**) was originally isolated from the *n*-hexane extract of *S. gigantea* leaves [29]; however, its presence in the roots has not been reported, representing the first identification of this compound from this plant organ. For both compounds, alternative isolation procedures have been demonstrated as previously described (see Section 3.5).

### 2.3. Antimicrobial Assays

Compounds **1**–**5** were assessed for their *in vitro* antimicrobial activity against the Gram-positive, non-pathogenic *B. subtilis* and seven phytopathogenic bacterial and fungal strains, selected for their frequent occurrence and relevance as common agents of various plant diseases: the Gram-positive bacterial pathogens *C. flaccumfaciens* pv. *flaccumfaciens* (causing bacterial wilt on beans), *C. michiganensis* (previously known as *C. michiganensis* subsp. *michiganensis*, the causal agent of bacterial canker on tomato), and *R. fascians* (responsible for the leafy gall syndrome); the Gram-negative bacterial pathogens *P. syringae* pv. *tomato* (causing bacterial speck and infecting tomato and *Arabidopsis thaliana*) and *X. arboricola* pv. *pruni* (causing bacterial leaf and fruit spot on stone fruits); and the fungal pathogens *B. sorokiniana* and *F. graminearum* (both causing common root rot and infecting various crops). The antibacterial, bactericidal, and antifungal activities of the isolates were evaluated in comparison with the respective positive controls, gentamicin (antibiotic) for bacterial strains and benomyl (fungicide) for fungal strains. The minimal inhibitory concentration (MIC) and minimal bactericidal concentration (MBC) values are summarized in Table 2.

As depicted in Table 2, compound **3** demonstrated the strongest antibacterial activity against all the tested Gram-positive bacterial strains. A particularly strong antibacterial and moderate bactericidal effect was observed against *C. michiganensis* (MIC 5.1 µM, MBC 83 µM). Compound **3** was also found to display strong inhibitory activity against *B. subtilis* and *C. flaccumfaciens* pv. *flaccumfaciens* with MIC values of 21 µM and a moderate antibacterial effect against *R. fascians* (MIC 41 µM). Compound **4** was also highly effective against *C. michiganensis* with a MIC value of 6.3 µM. Compounds **2**, **4**, and **5** exhibited moderate antibacterial activity against *B. subtilis* with MIC values ranging from 100 to 166 µM and a weak antibacterial effect against *C. flaccumfaciens* pv. *flaccumfaciens* with MIC values between 258 and 402 µM). These results suggest the selective potency of compound **3** against *B. subtilis*, *C. flaccumfaciens* pv. *flaccumfaciens* and *C. michiganensis*. In contrast, compound **1** demonstrated no detectable antibacterial activity at the concentrations tested (up to 402 µM) against the studied microorganisms. None of the isolated compounds were effective at the concentrations tested (up to 402 µM) against the Gram-negative *P. syringae* pv. *tomato* and *Xanthomonas arboricola* pv. *pruni*. This result may be explained by the structural and functional differences between the cell walls of Gram-negative and Gram-positive bacteria, with the former often serving as a barrier to numerous antibacterial agents [59]. When compared to the reference antibiotic gentamicin, the efficacy of compounds **3** and **4** against *C. michiganensis* was comparable to that of gentamicin. However, the antibacterial activity of the positive control exceeded that of the other compounds tested by 1–2 orders of magnitude. Compounds **2** and **3** exhibited a weak antifungal effect against *F. graminearum* with 43% and 38% mycelium growth inhibition at the highest concentration levels (415 and 413 µM), respectively. However, none of the tested isolates exhibited detectable antifungal activity at the tested concentrations (up to 502 µM) against *B. sorokiniana*. A structure–activity relationship (SAR) analysis suggests that the presence of an alcoholic hydroxy group appears to be crucial for the observed strong antibacterial activity. Additionally, the furan and angeloyloxy moieties might also contribute to the enhancement of the antibacterial effect. However, these hypotheses remain preliminary and require further investigation for confirmation. Notably, for compounds **1**, **2**, and **5**, determining the MIC and MBC values against certain bacterial strains was not feasible due to insufficient sample availability.

In our recent study, the antibacterial activity of compound **4** against *B. subtilis* was detected *in situ* in the TLC layer by TLC–DB [18]. However, the determination of its MIC and MBC values was precluded by insufficient sample quantity [27]. In the present work, this finding was extended and validated by microplate-based assays, confirming the antibacterial activity of compound **4**. Note that the antimicrobial activity of solidagoic acid J (**5**) has been reported in our recent publication [29] and is also demonstrated herein.

## 3. Materials and Methods

### 3.1. Materials and Reagents

Aluminum- and glass-backed TLC silica gel 60 F_254_ plates (20 × 10 cm or 20 × 20 cm) were purchased from Merck (Darmstadt, Germany). Reagents and solvents were commercially available and used without further purification. Solvents of analytical grade (*n*-hexane, acetone, ethyl acetate, methanol, chloroform (stabilized with 5–50 ppm amylene), isopropyl alcohol, toluene, and isopropyl acetate) and gradient-grade methanol were obtained from Molar Chemicals (Halásztelek, Hungary). LC-MS-grade methanol was purchased from VWR (Radnor, PA, USA), whereas LC-MS-grade water and HPLC-grade methanol were acquired from Reanal (Budapest, Hungary). Bidistilled water was obtained using a Vitrotech VDB-3A apparatus (Vitro-Tech-Lab Ltd., Gyál, Hungary). Ultrapure water was prepared by a Millipore Direct-Q 3 UV Water Purification System (Merck). For NMR spectroscopy, chloroform-*d* (99.80 atom% D, water < 0.01%) was supplied by Eurisotop (Saint-Aubin, France). Benomyl, gentamicin, formic acid (LC-MS grade), and *p*-anisaldehyde were purchased from Sigma-Aldrich (Burlington, MA, USA). 3-(4,5-Dimethylthiazol-2-yl)-2,5-diphenyltetrazolium bromide (MTT) was obtained from Carl Roth (Karlsruhe, Germany), acetic acid from Lach-Ner (Neratovice, Czech Republic), and concentrated sulfuric acid (96%) from Carlo Erba (Milan, Italy). Tryptone (from casein, pancreatic digest), sodium chloride, and vanillin were supplied by Reanal, agar by Merck, and yeast extract by Scharlab (Barcelona, Spain). Nutrient Broth (NB) was acquired from Biolab (Budapest, Hungary). *Bacillus subtilis* (F1276) was a gift from József Farkas (Central Food Research Institute, Budapest, Hungary). *Clavibacter michiganensis* (NCAIM B.01813) and *Curtobacterium flaccumfaciens* pv. *flaccumfaciens* (NCAIM B.01609) were obtained from Dénes Dlauchy (National Collection of Agricultural and Industrial Microorganisms (NCAIM), Budapest, Hungary). *Bipolaris sorokiniana* (Sacc.) Shoemaker H-299 (NCBI GenBank accession No. MH697869) was collected from barley in Hungary. *Fusarium graminearum* Schwabe (NCAIM F.00730) and *Rhodococcus fascians* (NCAIM B.01608) were purchased from NCAIM. *Pseudomonas syringae* pv*. tomato* DC3000 Lux was donated by Julia Vorholt (ETH Zurich, Zurich, Switzerland). The *Xanthomonas arboricola* pv. *pruni* strain (No. XapHU1) was isolated in 2016 from *Prunus armeniaca* L. cv. Bergecot by Ildikó Schwarczinger (Plant Protection Institute, HUN-REN Centre for Agricultural Research, Budapest, Hungary) [60].

### 3.2. Plant Material

The roots of *S. gigantea* were collected near Harta, Hungary (46°41′51.5′′ N 19°02′52.4′′ E, altitude: 90 m a. s. l.) in August 2022 (sample R2022) and in August 2023 (sample R2023). A voucher specimen (Figure 1c) was deposited at the Hungarian Natural History Museum, Budapest, Hungary (accession number: HNHM-TRA 00027284). The fresh plant material was air-dried at room temperature, then chopped and finely ground using a coffee grinder (Sencor SCG 2050, Sencor, Říčany, Czech Republic).

### 3.3. TLC–UV/FLD and Derivatization with p-Anisaldehyde and Vanillin–Sulfuric Acid

For TLC analysis, samples were manually applied using a 10 µL microsyringe (Hamilton, Bonaduz, Switzerland) as 5 mm bands with 5–10 mm track distance and 8 mm distance from the lower edge on TLC silica gel 60 F_254_ plates. TLC separation was performed in a twin-trough chamber (CAMAG, Muttenz, Switzerland) pre-saturated for 10 min with the mobile phases of chloroform–ethyl acetate–methanol, 17:2:1 (*V*/*V*) for compound **1**; chloroform–ethyl acetate, 3:1 (*V*/*V*) for compound **2**; chloroform–ethyl acetate–methanol, 15:3:2 (*V*/*V*) for compound **3**; chloroform–ethyl acetate, 47:3 (*V*/*V*) for compound **4**; isopropyl acetate–toluene–methanol (13:6:1, *V*/*V*) for compound **5** up to 80 mm distance from the lower plate edge. After development, the plates were dried with a cold air stream from a hair dryer and documented with a digital camera (Cybershot DSC-HX60, Sony, Neu-Isenberg, Germany) under a UV lamp (CAMAG) at 254 nm and 366 nm.

For post-chromatographic derivatization, the developed and dried glass-backed TLC plates were immersed in *p*-anisaldehyde–sulfuric acid reagent (500 μL of *p*-anisaldehyde, 10 mL of acetic acid, 100 mL of methanol, and 5 mL of concentrated sulfuric acid (96%)) or vanillin–sulfuric acid reagent (400 mg of vanillin, 100 mL of methanol and 2 mL of concentrated sulfuric acid (96%)), then heated at 110 °C for 5 min (Advanced Hot Plate, VWR). Derivatization was followed by documentation of chromatograms using a digital camera at Vis under white light illumination in the transmittance mode using a 96891 Salobrena 2 LED lamp (EGLO Lux, Dunakeszi, Hungary).

### 3.4. TLC–DB (B. subtilis Antibacterial Assay)

TLC–direct bioautography (DB) featuring a *B. subtilis* antibacterial assay was applied for the *in situ* detection of the separated antibacterial compounds in the TLC chromatogram of crude extracts, fractions, and isolated compounds. The developed and dried aluminum-backed TLC layers (see Section 3.3) were dipped in a bacterial cell suspension of *B. subtilis* (OD_600_ = 1.2) and then incubated at 37 °C for 2 h in a vapor chamber (100% relative humidity). Subsequently, the plates were stained with MTT (1 mg/mL aqueous solution), a vital dye that enables the visualization of bacterial viability. Following an additional 30-min incubation, the bioautograms were documented under visible light using a digital camera. The presence of antibacterial compounds was indicated by bright inhibition zones against a purple background.

### 3.5. Extraction and Isolation

The air-dried and powdered roots of *S. gigantea* (112 g from the collection of sample R2022 and 661 g from the collection of sample R2023) were exhaustively extracted at room temperature by maceration with ethyl acetate (sample R2022, 3 × 750 mL, each for 72 h) and ethanol (sample R2023, 3 × 4500 mL, each for 72 h), respectively. The resulting crude extracts were filtered using Reanal filter papers (pore size: 7–10 μm, ref. 106), combined, and concentrated *in vacuo* at 40 °C with a rotary evaporator (Rotavapor R-134, Büchi, Flawil, Switzerland) to yield dry residues of 6.8 g (sample R2022, ethyl acetate extraction) and 35.5 g (sample R2023, ethanolic extraction). Portions (4.75 g of sample R2022 and 8.0 g of sample R2023) of these dry residues were subsequently subjected to successive chromatographic separations. Each separation step was monitored by TLC–UV/FLD, TLC–*p*-anisaldehyde derivatization, TLC–DB, TLC–MS, and RP-HPLC–DAD-ESI-MS. Fractions with similar fingerprints were pooled.

The 4.75 g portion of the dry residue of sample R2022 was fractionated by normal-phase (NP) flash column chromatography (CombiFlash NextGen 300, Teledyne Isco, Lincoln, NE, USA) using a silica gel column (RediSep Rf Bronze, 20–40 μm, 40 g) with a gradient solvent system of *n*-hexane and acetone (0.0–5.0 min, 0%; 5.0–25.0 min, 0–50%; 25.0–35.0 min, 50–100% acetone; flow rate: 45 mL/min), affording 105 fractions. Fraction 26–27 (3.13 g) was subjected to NP flash column chromatography using a silica gel column (RediSep Rf Bronze, 20–40 μm, 40 g) with a gradient solvent system of *n*-hexane and ethyl acetate (0.0–3.0 min, 0%; 3.0–28.0 min, 0–20%; 28.0–33.0 min, 20–100%; ethyl acetate; flow rate: 30 mL/min) to furnish 70 subfractions.

Subfraction 26–27/32–39 (545 mg) and subfraction 26–27/40–48 (80 mg) were separately fractionated by NP flash column chromatography using a silica gel column (RediSep Rf Gold, 20–40 μm, 12 g) with a gradient solvent system of chloroform and ethyl acetate (0.0–5.0 min, 0%; 5.0–6.0 min, 0–2%; 6.0–11.0 min, 2%; 11.0–12.0 min, 2–4%; 12.0–17.0 min, 4%; 17.0–18.0 min, 4–6%; 18.0–23.0 min, 6%; 23.0–33.0 min, 6–15%; 33.0–36.0 min, 15–100% ethyl acetate; flow rate: 15 mL/min) to give 45 and 74 subfractions, respectively. Subfraction 26–27/40–48/7–10 (subfraction D) (7.6 mg) was further purified by semi-preparative, reversed-phase high-performance liquid chromatography (RP-HPLC) at 35 °C on a Gemini C_18_ column (250 mm × 10 mm, 10 μm, Phenomenex, Torrance, CA, USA) using an isocratic elution with 78% B (A: 5% aqueous methanol + 0.1% formic acid, B: methanol + 0.1% formic acid; flow rate: 4 mL/min; UV detection: 220 nm)—method **A**—to furnish compound **4** (3.9 mg, *t*_R_ = 17.6 min). Subfractions 26–27/32–39/23–26 (26.8 mg) and 26–27/40–48/39–67 (30.8 mg) were pooled and the combined sample (57.6 mg) was further separated by RP flash column chromatography on a C_18_ column (RediSep Rf Gold C18, 20–40 μm, 30 g) using a gradient solvent system of water + 0.1% formic acid and methanol + 0.1% formic acid (0.0–1.4 min, 50%; 1.4–2.9 min, 50–80%; 2.9–31.4 min, 80–100% methanol + 0.1% formic acid; flow rate: 20 mL/min) to provide 48 subfractions. Subfraction (26–27/32–39/23–26 + 26–27/40–48/39–67)/20–21 (subfraction B) (4.5 mg) was further purified by method **A** (see above) to obtain compound **2** (1.5 mg, *t*_R_ = 26.3 min).

Subfraction 26–27/59–67 (31.8 mg) was separated by NP flash column chromatography using a silica gel column (RediSep Rf Gold, 20–40 μm, 4 g) with a gradient solvent system of chloroform and isopropyl alcohol (0.0–1.5 min, 0%; 1.5–2.5 min, 0–1%; 2.5–7.5 min, 1%; 7.5–9.0 min, 1–2%; 9.0–21.0 min, 2%; 21.0–22.0 min, 2–3%, 22.0–30.0 min, 3%; 30.0–35.0 min, 3–4%; 35.0–40.0 min, 4–100% isopropyl alcohol; flow rate: 9 mL/min) to obtain 47 subfractions. Subfraction 26–27/59–67/22–29 (subfraction A) (5.7 mg) was further purified by RP flash column chromatography on a C_18_ column (RediSep Rf Gold C18, 20–40 μm, 30 g) using a gradient solvent system of water + 0.1% formic acid and methanol + 0.1% formic acid (0.0–1.7 min, 10%; 1.7–3.9 min, 10–70%; 3.9–26.0 min, 70–100% methanol + 0.1% formic acid; flow rate: 20 mL/min) to obtain compound **1** (1.3 mg, *t*_R_ = 17.3–25.7 min, subfraction 26–27/59–67/22–29/19–27).

Fraction 37–44 (696 mg) was fractionated by RP flash column chromatography on a C_18_ column (RediSep Rf Gold C18, 20–40 μm, 30 g) using a gradient solvent system of water + 0.1% formic acid and methanol + 0.1% formic acid (0.0–0.6 min, 0%; 0.6–1.2 min, 0–50%; 1.2–34.4 min, 50–100% methanol + 0.1% formic acid; flow rate: 20 mL/min) to obtain 50 subfractions. Subfraction 37–44/37–40 (subfraction E) (22.1 mg) was further purified by NP flash column chromatography using a silica gel column (RediSep Rf Gold, 20–40 μm, 12 g) with a gradient solvent system of *n*-hexane and isopropyl alcohol (0.0–1.0 min, 0%; 1.0–31.0 min, 0–10%; 31.0–40.0 min, 10%; 40.0–45.0 min, 10–100% isopropyl alcohol; flow rate: 20 mL/min) to give compound **5** (2.7 mg, *t*_R_ = 12.9–13.8 min, subfraction 37–44/37–40/18).

The 8.0 g portion of the dry residue of sample R2023 was subjected to NP flash column chromatography using a silica gel column (RediSep Rf Gold, 20–40 μm, 40 g) with a gradient solvent system of *n*-hexane and acetone (0.0–2.0 min, 0%; 2.0–4.0 min, 0–10%; 4.0–14.0 min, 10%; 14.0–16.0 min, 10–15%; 16.0–23.5 min, 15%; 23.5–38.5 min, 15–50%; 38.5–43.5 min, 50–100% acetone; flow rate: 40 mL/min) to yield 111 fractions. Fraction 77–89 (310 mg) was fractionated by RP flash column chromatography on a C_18_ column (RediSep Rf Gold C18, 20–40 μm, 30 g) using a gradient solvent system of water + 0.1% formic acid and methanol + 0.1% formic acid (0.0–1.1 min, 10%; 1.1–2.2 min, 10–60%; 2.2–20.7 min, 60–100% methanol + 0.1% formic acid; flow rate: 35 mL/min) to provide 56 subfractions. Subfraction 77–89/37–40 (subfraction C) (14.7 mg) was further purified by semi-preparative RP-HPLC at 35 °C on a Gemini C_18_ column (250 mm × 10 mm, 10 μm, Phenomenex) using an isocratic elution with 75% B (A: 5% aqueous methanol + 0.1% formic acid, B: methanol + 0.1% formic acid; flow rate: 4 mL/min; UV detection: 205 nm) to furnish compound **3** (2.0 mg, *t*_R_ = 28.3 min).

### 3.6. Compound Characterization

Solidagolactone IX ((−)-(5S*,8R*,9R*,10S*,19R*)-18,19-epoxy-19-hydroxy-cleroda-3,13-dien-16,15-olide) (**1**): White amorphous solid; [*α*]_D_^25^ −10.7 (*c* 0.075, CHCl_3_); UV (EtOH) *λ*_max_ (log *ε*) 206 nm (3.71); IR (ATR) *ν*_max_ 3398, 2956, 2923, 2857, 1743, 1595, 1355, 1071 cm^−1^; ^1^H (500 MHz, CDCl_3_) and ^13^C (126 MHz, CDCl_3_) NMR spectroscopic data, see Table 1; HR-HESI-MS *m*/*z* 355.1879 [M+Na]^+^ (calculated for C_20_H_28_O_4_Na^+^, *m*/*z* 355.1880 [M+Na]^+^, error: −0.3 ppm), *m*/*z* 331.1915 [M−H]^−^ (calculated for C_20_H_27_O_4_^−^, *m*/*z* 331.1915 [M−H]^−^, error: 0.1 ppm); TLC (silica gel): *R*_F_ 0.47 (chloroform–ethyl acetate–methanol 17:2:1, *V*/*V*); color after derivatization with vanillin–sulfuric acid reagent: pink.

Solidagoic acid K ((−)-(5S*,8R*,9R*,10S*)-15,16-epoxy-18-isobutyryloxy-cleroda-3,13,14-trien-19-oic acid) (**2**): White amorphous solid; [*α*]_D_^25^ −25.3 (*c* 0.095, CHCl_3_); UV (EtOH) *λ*_max_ (log *ε*) 205 nm (3.85); IR (ATR) *ν*_max_ 2966, 2932, 2878, 1732, 1694, 1455, 1385, 1348, 1256, 1191, 1157, 1093, 1025 cm^−1^; ^1^H (500 MHz, CDCl_3_) and ^13^C (126 MHz, CDCl_3_) NMR spectroscopic data, see Table 1; HR-HESI-MS *m*/*z* 425.2298 [M+Na]^+^ (calculated for C_24_H_34_O_5_Na^+^, *m*/*z* 425.2298 [M+Na]^+^, error: −0.1 ppm), *m*/*z* 401.2329 [M−H]^−^ (calculated for C_24_H_33_O_5_^−^, *m*/*z* 401.2329 [M−H]^−^, error: 0.1 ppm); TLC (silica gel): *R*_F_ 0.70 (chloroform–ethyl acetate 3:1, *V*/*V*); color after derivatization with vanillin–sulfuric acid reagent: purple.

Solidagodiol ((−)-(5S*,6R*,8R*,9R*,10S*,13*Z*)-6-angeloyloxy-cleroda-3,13-dien-15,16-diol) (**3**): White amorphous solid; [*α*]_D_^25^ −9.1 (*c* 0.11, CHCl_3_); UV (EtOH) *λ*_max_ (log *ε*) 204 nm (4.00); IR (ATR) *ν*_max_ 3357, 2959, 2928, 2880, 1771, 1698, 1649, 1596, 1455, 1384, 1233, 1163, 1073, 1040, 1001 cm^−1^; ^1^H (500 MHz, CDCl_3_) and ^13^C (126 MHz, CDCl_3_) NMR spectroscopic data, see Table 1; HR-HESI-MS *m*/*z* 427.2819 [M+Na]^+^ (calculated for C_25_H_40_O_4_Na^+^, *m*/*z* 427.2818 [M+Na]^+^, error: −0.3 ppm); TLC (silica gel): *R*_F_ 0.41 (chloroform–ethyl acetate–methanol 15:3:2, *V*/*V*); color after derivatization with *p*-anisaldehyde–sulfuric acid reagent: purple.

(−)-(5*R*,8*R*,9*R*,10*S*)-15,16-epoxy*-ent*-*neo*-cleroda-3,13,14-trien-18-ol (**4**): Pale-yellow oil; ^1^H NMR (500 MHz, CDCl_3_) *δ* 7.35 (t, *J* = 1.7 Hz, 1H, H-15), 7.21 (m, 1H, H-16), 6.28 (dd, *J* = 1.9, 0.9 Hz, 1H, H-14), 5.73 (ddd, *J* = 4.6, 2.9, 1.3 Hz, 1H, H-3), 4.13 (m, 2H, H_2_-18), 2.43 (m, 1H, H_2_-12a), 2.34 (dddd, *J* = 14.2, 12.6, 4.4, 1.1 Hz, 1H, H_2_-12b), 2.13 (m, 1H, H_2_-2a), 2.07 (m, 1H, H_2_-2b), 1.96 (td, *J* = 13.3, 4.9 Hz, 1H, H_2_-11a), 1.87 (m, 1H, H_2_-1a), 1.72 (ov., 1H, H_2_-6a), 1.70 (ov., 1H, H_2_-1b), 1.63 (m, 1H, H-8), 1.54 (ov., 1H, H_2_-6b), 1.52 (ov., 1H, H-10), 1.51 (ov., 1H, H_2_-7a), 1.41 (ov., 1H, H_2_-11b), 1.40 (ov., 1H, H_2_-7b), 1.23 (s, 3H, H_3_-19), 1.07 (s, 3H, H_3_-20), 0.90 (d, *J* = 6.9 Hz, 3H, H_3_-17); ^13^C NMR (126 MHz, CDCl_3_) *δ* 145.8 (C, C-4), 142.8 (CH, C-15), 138.6 (CH, C-16), 126.1 (C, C-13), 124.8 (CH, C-3), 111.2 (CH, C-14), 64.2 (CH_2_, C-18), 44.8 (CH, C-10), 38.9 (CH_2_, C-9), 38.7 (CH_2_, C-11), 38.2 (CH_2_, C-5), 37.4 (CH, C-8), 32.2 (CH_2_, C-6), 29.3 (CH_3_, C-19), 27.4 (CH_2_, C-7), 25.9 (CH_3_, C-20), 25.5 (CH_2_, C-2), 19.5 (CH_2_, C-1), 18.9 (CH_2_, C-12), 15.4 (CH_3_, C-17); HR-HESI-MS *m*/*z* 355.1879 [M+Na]^+^ (calculated for C_20_H_28_O_4_Na^+^, *m*/*z* 355.1880 [M+Na]^+^, error: −0.1 ppm), *m*/*z* 331.1915 [M−H]^−^ (calculated for C_20_H_27_O_4_^−^, *m*/*z* 331.1915 [M−H]^−^, error: −0.1 ppm); TLC (silica gel): *R*_F_ 0.68 (chloroform–ethyl acetate 47:3, *V*/*V*); color after derivatization with vanillin–sulfuric acid reagent: greyish-blue.

Solidagoic acid J ((5S*,8R*,9R*,10S*,13*Z*)-16,18-diangeloyloxy-15-hydroxy-cleroda-3,13-dien-19-oic acid) (**5**): White amorphous solid; ^1^H (500 MHz, CDCl_3_) *δ* 6.11 (qq, *J* = 7.3, 1.5 Hz, 1H, H-3″), 6.04 (qq, *J* = 7.2, 1.6 Hz, 1H, H-3′), 5.93 (t, *J* = 4.1 Hz, 1H, H-3), 5.71 (t, *J* = 7.1 Hz, 1H, H-14), 4.82 (d, *J* = 12.3 Hz, 1H, H_2_-16a), 4.66 (d, *J* = 12.3 Hz, 1H, H_2_-16b), 4.52 (m, 2H, H_2_-18), 4.27 (dd, *J* = 7.0, 2.2 Hz, 1H, H-15), 2.41 (dt, *J* = 14.0, 3.0 Hz, 1H, H_2_-6a), 2.32 (ov., 1H, H-10), 2.26 (td, *J* = 13.2, 3.2 Hz, 1H, H_2_-12a), 2.17 (m, 2H, H_2_-2), 1.98 (dq, *J* = 7.2, 1.6 Hz, 6H, H_3_-4′ and H_3_-4″), 1.94 (td, *J* = 13.6, 4.7 Hz, 1H, H_2_-12b), 1.88 (ov., 3H, H_3_-5′), 1.87 (ov., 3H, H_3_-5″), 1.75 (ov., 1H, H_2_-1a), 1.66 (ov., 1H, H_2_-7a), 1.65 (ov., 1H, H-8), 1.62 (ov., 1H, H_2_-11a), 1.54 (ov., 1H, H_2_-1b), 1.52 (ov., 1H, H_2_-6b), 1.34 (ov., 1H, H_2_-7b), 1.20 (ov., 1H, H_2_-11b), 0.93 (s, 3H, H_3_-20), 0.80 (d, *J* = 6.6 Hz, H_3_-17); ^13^C (126 MHz, CDCl_3_) *δ* 178.4 (C, C-19), 168.7 (C, C-1″), 167.7 (C, C-1′), 139.5 (CH, C-3″), 139.1 (C, C-13), 138.3 (CH, C-3′), 136.1 (C, C-4), 128.5 (CH, C-4), 128.1 (CH, C-3), 128.0 (C, C-2′), 127.6 (C, C-2″), 64.6 (CH_2_, C-18), 61.4 (CH_2_, C-16), 58.9 (CH_2_, C-15), 49.8 (C, C-5), 42.7 (CH, C-10), 38.8 (C, C-9), 37.0 (CH, C-8), 30.2 (CH_2_, C-6), 30.1 (CH_2_, C-11), 29.5 (CH_2_, C-12), 28.0 (CH_2_, C-7), 27.1 (CH_3_, C-20), 26.5 (CH_2_, C-2), 20.8 (CH_3_, C-5′), 20.7 (CH_3_, C-5″), 19.6 (CH_2_, C-1), 16.1 (CH_3_, C-4″), 15.9 (CH_3_, C-4′), 15.8 (CH_3_, C-17); HR-HESI-MS *m*/*z* 539.2977 [M+Na]^+^ (calculated for C_30_H_44_O_7_Na^+^, *m*/*z* 539.2979 [M+Na]^+^, error: −0.5 ppm), *m*/*z* 515.3014 [M−H]^−^ (calculated for C_30_H_43_O_7_^−^, *m*/*z* 515.3014 [M−H]^−^, error: 0.0 ppm); TLC (silica gel): *R*_F_ 0.58 (isopropyl acetate–toluene–methanol 13:6:1, *V*/*V*); color after derivatization with *p*-anisaldehyde–sulfuric acid reagent: purple.

### 3.7. TLC–ESI-MS

For TLC–MS analysis, methanol was delivered at a flow rate of 0.2 mL/min using a binary HPLC pump (LC-20AB, Shimadzu, Kyoto, Japan) through the oval elution head (4 mm × 2 mm) of the CAMAG TLC–MS Interface 2, and directed into a single quadrupole, low-resolution electrospray ionization mass spectrometer (ESI-MS) (LCMS-2020, Shimadzu). The mass spectrometric parameters were as follows: nebulizer gas (N_2_) flow rate, 1.5 L/min; drying gas (N_2_) flow rate, 10 L/min; interface temperature, 350 °C; heat block temperature, 400 °C; and desolvation line temperature, 250 °C. The detector voltage was set to +4.5 kV for positive ion mode and –4.5 kV for negative ion mode. Full-scan mass spectra were acquired in both ionization modes in the *m*/*z* range of 200–950 at a scan speed of 790 amu/s. Instrument control, data acquisition, and analysis, including background subtraction, were performed using Shimadzu LabSolutions software (version 5.42v).

### 3.8. FIA–HR-HESI-MS(/MS)

The flow injection analysis (FIA) HR-HESI-MS/MS spectra of the isolated compounds were acquired using a Vanquish Flex UHPLC system (VF-P10, Dionex Softron, Germering, Germany) coupled to an Orbitrap Exploris 120 hybrid quadrupole-orbitrap mass spectrometer (Thermo Fisher Scientific, Bremen, Germany) equipped with a HESI-II probe. Methanol was used as the carrier solvent at a flow rate of 0.2 mL/min. Full-scan mass spectra were recorded in both positive and negative ionization modes within the *m/z* range of 100–1000 with a lock mass correction at a resolution of 120,000. The spray voltage was +3.4 kV (positive mode) and −2.0 kV (negative mode). The ion transfer tube was set to 320 °C, and the vaporizer to 250 °C. Nitrogen was prepared by a Peak Scientific Genius XE 35 gas generator (Glasgow, UK) and was used as auxiliary and sheath gas at flow rates of 5 and 10 arbitrary units, respectively.

HRMS/MS spectra were acquired in HCD fragmentation mode with normalized collision energies between 15% and 50%. Precursor ions were selected with a quadrupole isolation window of *m/z* 0.4. Tandem mass spectra were recorded without a lock mass correction at a resolution of 120,000. The instrument control, data acquisition, and analysis were performed by Xcalibur software (version 4.7.69, Thermo Fisher Scientific).

### 3.9. Spectroscopy

#### 3.9.1. NMR Spectroscopy

Samples were prepared in chloroform-*d* (CDCl_3_) solvent and measured in standard 5 mm NMR tubes. All NMR spectra were recorded on a Bruker AVANCE III 500 (^1^H: 500.1 MHz, ^13^C: 125.8 MHz; 11.7 T) instrument equipped with a 5 mm triple-resonance, *z*-gradient cryoprobe (CP TCI 500S2 H-C/N-D-05 Z) (Bruker Corporation, Billerica, MA, USA) at 296 K. The NMR spectrometer was operated and controlled by Bruker TopSpin software (version 3.5). All standard pulse sequences were taken from the spectrometer software library. ^1^H and ^13^C chemical shifts are reported on the delta (*δ*) scale as parts per million (ppm) referenced to the NMR solvent used (CHCl_3_ residual peak at *δ*_H_ = 7.26 ppm and CDCl_3_ at *δ*_C_ = 77.16 ppm). ^1^H–^1^H spin-spin coupling constant (*J*) values are reported in hertz (Hz). Signal multiplicities are denoted as follows: s—singlet; br s—broad singlet; d—doublet; t—triplet; p—pentet; sept—septet; m—multiplet; dd—doublet of doublets; td—triplet of doublets; dq—doublet of quartets; and qq—quartet of quartets, ddd—doublet of doublets of doublets, dddd—doublet of doublets of doublets of doublets. The complete ^1^H and ^13^C resonance assignments were carried out using conventional one-dimensional (1D) ^1^H (*zg*) and ^13^C DEPTQ (*deptqsp*) as well as two-dimensional (2D) homonuclear ^1^H–^1^H COSY (*cosygpqf*), ^1^H–^1^H TOCSY (*mlevph*, mixing time: 120 ms), ^1^H–^1^H ROESY (*roesyph.2*, mixing time: 300 ms), and heteronuclear ^1^H–^13^C multiplicity-edited HSQC (edHSQC, *hsqcedetgpsisp2.3*, optimized for ^1^*J*_C–H_ = 145 Hz) and ^1^H–^13^C HMBC (*hmbcetgpl3nd*, optimized for *^n^J*_C–H_ = 8 Hz) experiments. The NMR data of the known compounds were compared with previously reported literature values.

#### 3.9.2. UV Spectroscopy

UV spectra were collected at room temperature using a PerkinElmer Lambda 35 spectrophotometer (PerkinElmer, Waltham, MA, USA). The spectra were recorded in the range of 190–400 nm (scan speed: 60 nm/min, slit width: 1 nm). Data were processed and analyzed by the UV WinLab software (version 5.2.0.0646).

#### 3.9.3. ATR-FTIR Spectroscopy

ATR-FTIR spectra were collected using a PerkinElmer Spectrum 400 FT-IR/FT-NIR spectrometer equipped with a diamond/ZnSe ATR crystal and an MIR TGS detector. The spectra were recorded in the range of 4000–650 cm^−1^ (spectral resolution: 4 cm^−1^, 32 scans per sample). Data were processed and analyzed by PerkinElmer Spectrum Software (version 6.3.1).

### 3.10. Polarimetry

Optical rotations were measured in chloroform at 25 °C using a PerkinElmer 341 LC polarimeter with a 1 dm optical path length, employing the sodium D-line (589.3 nm).

### 3.11. Antibacterial and Antifungal Activity Microplate Assays

#### 3.11.1. Cell Culture

*B. subtilis* was cultured on Luria–Bertani (LB) agar plate (5 g/L yeast extract, 10 g/L tryptone, 10 g/L sodium chloride, 15 g/L agar) at 37 °C for 24 h, *P. syringae* pv. *tomato* on LB agar plate at 28 °C for 24 h, *C. michiganensis, C. flaccumfaciens* pv. *flaccumfaciens*, and *X. arboricola* pv. *pruni* on Nutrient agar plate (5 g/L sodium chloride, 11 g/L peptones, 15 g/L agar) at 28 °C for 24 h, and *R. fascians* on Waksman agar plate (5 g/L meat extract, 5 g/L peptone, 5 g/L sodium chloride, 10 g/L glucose, 15 g/L agar, pH adjusted to 7.2 with a 40% aqueous sodium hydroxide solution) at 30 °C for 24 h. *F. graminearum* and *B. sorokiniana* were grown in LB broth at 21 °C for 72 h by shaking at 120 rpm.

Before conducting the antibacterial assays, each bacterial suspension was diluted with the specified broth to achieve a final cell concentration of 10^5^ CFU/mL. For the antifungal assays, fungal mycelia were washed with fresh LB medium and homogenized using a FastPrep^®^-24 Classic homogenizer (MP Biomedicals, Irvine, CA, USA). The resulting mycelial fragments were resuspended in 1 mL of LB medium and then transferred to 2 mL Eppendorf tubes containing seven 2 mm glass beads. Homogenization was carried out at 4.5 m/s for 2 × 20 s. Finally, a mycelium suspension with OD_600_ = 0.2 was prepared in LB broth.

#### 3.11.2. Determination of Minimal Inhibitory Concentration (MIC) Values

The *in vitro* antimicrobial activity of the isolated compounds (**1**–**5**) was assessed by determining their minimal inhibitory concentration (MIC) and minimal bactericidal concentration (MBC) values. The assays were conducted against a panel of bacterial strains, including the Gram-positive *B. subtilis*, *C. flaccumfaciens* pv. *flaccumfaciens*, *C. michiganensis*, and *R. fascians*, as well as the Gram-negative *P. syringae* pv. *tomato* and *X. arboricola* pv. *pruni*. In addition, antifungal activity was evaluated against *B. sorokiniana* and *F. graminearum*. The experiments were performed using a microplate-based assay as previously described [29]. For antibacterial assays, non-treated, flat-bottom 96-well microplates (VWR, cat. no. 734-2781) were used. For antifungal assays, non-treated, U-bottom 96-well microplates (Nest Scientific, Woodbridge, NJ, USA; cat. no. 701111) were employed. Gentamicin (0.1 mg/mL in water) and benomyl (25 mg/mL in ethanol) served as positive controls for the antibacterial and antifungal assays, respectively, while ethanol was used as the negative control. A two-fold ethanolic dilution series of compounds **1**–**5** (2 mg/mL in ethanol) and positive controls (10 µL per well) was prepared in triplicate. Following dilution, the ethanol was evaporated from the wells in a laminar box (BA-900, Radel & Hahn, Debrecen, Hungary). For the antibacterial assays, 150 µL of bacterial suspension (10^5^ CFU/mL) was added to each well. For the antifungal assays, 70 µL of LB broth, followed by 50 µL of a mycelium suspension (OD_600_ = 0.2 in LB broth), was added to each well. For antibacterial assays, the final concentrations of compounds **1**–**5** and gentamicin in the wells were in the range of 2.1–133.3 μg/mL (4.0–402 μM) and 0.1–6.7 μg/mL (0.2–14 μM), respectively. For antifungal assays, the final concentrations of compounds **1**–**5** and benomyl in the wells were in the range of 2.6–166.7 μg/mL (5.0–502 μM) and 32.6–2083 μg/mL (112–7184 μM), respectively. Absorbance at 600 nm was measured using a CLARIOstar^®^ Plus microplate reader (BMG LABTECH, Ortenberg, Germany), both immediately and following incubation with shaking at 500 rpm using a Grant PHMP microplate thermoshaker (Grant Instruments, Royston, UK): *B. subtilis* for 24 h at 37 °C; *C. flaccumfaciens* pv. *flaccumfaciens* and *C. michiganensis* for 48 h at 28 °C; *P. syringae* pv. *tomato* and *X. arboricola* pv. *pruni* for 24 h at 28 °C; and *R. fascians* for 48 h at 30 °C. Antifungal assays were incubated for 72 h at 21 °C without shaking. The experiments were repeated on two separate occasions.

#### 3.11.3. Determination of Minimal Bactericidal Concentration (MBC) Values

A 10 µL aliquot was taken from microplate wells that showed no bacterial growth after 24 or 48 h of incubation and was dotted onto the surface of LB agar (for *B. subtilis*), Waksman agar (for *R. fascians*), and NB agar (for *C. michiganensis* and *C. flaccumfaciens* pv. *flaccumfaciens*). The minimal bactericidal concentration (MBC) was determined as the lowest concentration of the tested compound at which no colony formed after 24 h incubation at 37 °C for *B. subtilis*, after 48 h incubation at 30 °C for *R. fascians*, and after 48 h incubation at 28 °C for *C. michiganensis* and *C. flaccumfaciens* pv. *flaccumfaciens*.

## 4. Conclusions

In conclusion, the non-targeted phytochemical analysis and the bioassay-guided fractionation of the ethanolic and ethyl acetate root extract of *S. gigantea* resulted in the isolation of three previously undescribed clerodane diterpenoids, including two *cis*-clerodanes, solidagolactone IX (**1**) and solidagoic acid K (**2**), and one *trans*-clerodane, solidagodiol (**3**), along with two known *cis*-clerodane diterpenoids, (−)-(5*R*,8*R*,9*R*,10*S*)-15,16-epoxy*-ent*-*neo*-cleroda-3,13,14-trien-18-ol (**4**) and solidagoic acid J (**5**). Compound **4** had previously been isolated from the roots of this species, with its antibacterial activity against *B. subtilis* demonstrated only *in situ* by thin-layer chromatography coupled with direct bioautography (TLC–DB). In the present study, this activity was confirmed for the first time by an *in vitro* microplate-based assay. Compound **5** had previously been identified from the leaves of *S. gigantea* but not from the roots; therefore, it represents the first report of its occurrence in this organ.

Compound **3** exhibited the strongest antibacterial activity against all tested Gram-positive bacterial strains, including *B. subtilis, C. flaccumfaciens* pv. *flaccumfaciens, C. michiganensis*, and *R. fascians*, with MIC values ranging from 5.1 to 41 µM, and demonstrated moderate bactericidal activity against *C. michiganensis* with an MBC value of 83 µM. Compound **4** was also highly active and selective for *C. michiganensis* (MIC 6.3 µM). The weak antifungal activity of compounds **2** and **3** was also observed against *F. graminearum*. The isolated compounds represent potential leads for the development of more potent plant-derived pesticide candidates, offering sustainable and eco-friendly alternatives that may help reduce the ecological footprint of conventional synthetic pesticides.

This work not only expands our phytochemical and biological knowledge of *S. gigantea* but also demonstrates the effectiveness of integrating TLC hyphenations, such as TLC–DB and TLC–MS, into bioassay-guided isolation workflows for the discovery of previously undescribed, bioactive specialized metabolites. Compared to traditional trial-and-error methods, the outlined bioassay-guided strategy offers a more cost-effective, time-efficient, and reliable approach for detecting, isolating, and identifying biologically active compounds.

## Figures and Tables

**Table 1:** ^1^H NMR (500 MHz, CDCl_3_) and ^13^C NMR (126 MHz, CDCl_3_) spectroscopic data for the isolated compounds (**1**–**3**) in chloroform-*d* (*δ* in ppm, *J* in Hz). The multiplicity of overlapping (ov.) signals could not be discerned and is, therefore, not reported.

Position	Solidagolactone IX (1)		Solidagoic Acid K (2)		Solidagodiol (3)	
	*δ*_H_ (ppm), Multiplicity, *J* (Hz)	*δ*_C_ (ppm), Type	*δ*_H_ (ppm), Multiplicity, *J* (Hz)	*δ*_C_ (ppm), Type	*δ*_H_ (ppm), Multiplicity, *J* (Hz)	*δ*_C_ (ppm), Type
1a	1.69, ov.	20.1, CH_2_	1.80, m	19.6, CH_2_	1.68, ov.	17.5, CH_2_
1b			1.57, ov.		1.56, ov.	
2a	2.15, m	26.3, CH_2_	2.19, m	26.5, CH_2_	2.04, m	26.5, CH_2_
2b					1.99, ov.	
3	5.59, m	118.3, CH	5.91, t (4.0)	128.5, CH	5.17, br s	122.3, CH
4	–	142.1, C	–	135.8, C	–	143.0, C
5	–	49.5, C	–	50.2, C	–	42.8, C
6a	1.69, ov.	30.4, CH_2_	2.37, ov.	30.1, CH_2_	5.09, dd (11.1, 4.6)	74.2, CH
6b	1.53, m		1.52, m			
7a	1.35, m	28.1, CH_2_	1.65, ov.	27.9, CH_2_	1.89, ov.	32.3, CH_2_
7b			1.35, m		1.66, ov.	
8	1.68, ov.	37.2, CH	1.66, ov.	37.1, CH	1.75, m	36.1, CH
9	–	38.2, C	–	38.9, C	–	37.4, C
10	1.89, dd (12.1, 3.6)	38.6, CH	2.41, ov.	42.2, CH	1.56, ov.	44.8, CH
11a	1.67, ov.	32.1, CH_2_	1.59, ov.	31.9, CH_2_	1.54, ov.	38.0, CH_2_
11b	1.17, td (13.4, 4.5)		1.42, td (13.8, 5.1)		1.17, m	
12a	2.63, m	20.6, CH_2_	2.55, ov.	19.0, CH_2_	2.08, m	29.2, CH_2_
12b	2.18, td (13.1, 4.4)		2.24, td (13.2, 5.1)			
13	–	135.8, C	–	126.1, C	–	144.9, C
14	7.17, br s	146.0, CH	6.25, dd (1.8, 0.9)	111.3, CH	5.64, t (6.9)	126.4, CH
15	4.79, br s	70.6, CH_2_	7.30, t (1.7)	142.6, CH	4.22, dd (6.9, 2.6)	58.8, CH_2_
16	–	176.1, C	7.15, t (1.5)	138.6, CH	4.18, d (2.0)	61.2, CH_2_
17	0.81, d (6.9)	15.9, CH_3_	0.85, d (6.2)	15.8, CH_3_	1.08, d (7.1)	15.2, CH_3_
18	4.35, m	67.2, CH_2_	4.43, m	64.7, CH_2_	1.56, ov.	20.76/20.81, CH_3_
19	5.52, d (5.7)	100.8, CH	–	179.5, C	1.20, s	17.1, CH_3_
20	0.97, s	26.4, CH_3_	0.98, s	26.9, CH_3_	0.97, s	20.4, CH_3_
1′			–	176.9, C	–	167.5, C
2′			2.54, sept (7.0)	34.2, CH	–	128.7, C
3′			1.15, d (7.0)	19.10 *, CH_3_	6.04, qq (7.3, 1.3)	137.8, CH
4′			1.15, d (7.0)	19.06 *, CH_3_	2.00, dq (7.3, 1.6)	15.8, CH_3_
5′					1.89, p (1.5)	20.76/20.81, CH_3_
19-OH	5.06, d (5.7)	–				

* Interchangeable resonances. ov.: overlapping signals (multiplicities could not be discerned).

**Table 2:** The minimal inhibitory concentration (MIC) and minimal bactericidal concentration (MBC) values (given in µM) of the isolated compounds (**1**–**5**) and positive controls, gentamicin (for bacterial strains) and benomyl (for fungal strains), against the *Bacillus subtilis* (*Bs*), *Curtobacterium flaccumfaciens* pv. *flaccumfaciens* (*Cff*), *Clavibacter michiganensis* (*Cm*), *Pseudomonas syringae* pv. *tomato* (*Pstom*), *Rhodococcus fascians* (*Rf*), and *Xanthomonas arboricola* pv. *pruni* (*Xap*) bacterial strains and against the *Bipolaris sorokiniana* (*Bip*) and *Fusarium graminearum* (*Fg*) fungal strains. Gram-positive and Gram-negative bacteria are abbreviated as G+ and G−, respectively. For compounds **1**, **2**, and **5**, MIC and MBC values against certain bacterial strains could not be determined due to limited sample availability. Note that the antimicrobial activity of solidagoic acid J (**5**) has been reported in our recent publication [29] and is also demonstrated herein.

Compounds	*Bs* (G+)		*Cff* (G+)		*Cm* (G+)		*Rf* (G+)		*Pstom* (G−)		*Xap* (G−)		*Bip*	*Fg*
	MIC	MBC	MIC	MBC	MIC	MBC	MIC	MBC	MIC	MBC	MIC	MBC	MIC	MIC
**1**	>402	>402	>402	>402	>402	>402	N/A	N/A	>402	>402	N/A	N/A	>502	>502
**2**	166	>332	332	>332	41	>332	N/A	N/A	>332	>332	>332	>332	>415	>415 ^b^
**3**	21	>330	21	>330	5.1	83	41	>330	>330	>330	>330	>330	>413	>413 ^c^
**4**	100	>402	402	>402	6.3	>402	201	>402	>402	>402	>402	>402	>502	>502
**5** [29]	129	>258	258	>258	129	>258	N/A	N/A	N/A	N/A	N/A	N/A	N/A	N/A
Gentamicin ^a^	1.7	3.5	1.7	1.7	3.5	3.5	1.7	3.5	0.9	1.7	3.5	3.5		
Benomyl ^a^													3593	1797

N/A—no data available; ^a^ positive control; ^b^ 43% inhibition at 415 µM; ^c^ 38% inhibition at 413 µM.

## Data Availability

The data supporting the findings of this study are available in the Supporting Information and from the corresponding author upon reasonable request.

## Supplementary Materials

The following supporting information can be downloaded at https://www.mdpi.com/article/10.3390/ijms26189187/s1.
