# Supplementary material for: Discovery of Undescribed Clerodane Diterpenoids with Antimicrobial Activity Isolated from the Roots of Solidago gigantea Ait
Source: Int J Mol Sci. 2025 Sep 20;26(18):9187. doi: 10.3390/ijms26189187 (PMC12471146; doi:10.3390/ijms26189187)
Supplement: Supplementary file 1 [file ijms-26-09187-s001.zip › ijms-3877171-supplementary.pdf]

Supporting Information

Discovery of Undescribed Clerodane Diterpenoids with Antimicrobial Activity Isolated from the Roots of *Solidago gigantea* Ait

Márton Baglyas [1,2], Zoltán Bozsó [1], Ildikó Schwarczinger [1], Péter G. Ott [1], József Bakonyi [1], András Darcsi [3] and Ágnes M. Móricz [1,*]

1 Plant Protection Institute, HUN-REN Centre for Agricultural Research, Fehérvári út 132–144, 1116 Budapest, Hungary; baglyas.marton@atk.hun-ren.hu (M.B.); bozso.zoltan@atk.hun-ren.hu (Z.B.); schwarczinger.ildiko@atk.hun-ren.hu (I.S.); ott.peter@atk.hun-ren.hu (P.G.O.); bakonyi.jozsef@atk.hun-ren.hu (J.B.)

2 Doctoral School, Semmelweis University, Üllői út 26, 1085 Budapest, Hungary

3 Pharmaceutical Chemistry and Technology Department, National Center for Public Health and Phar-macy, Szabolcs utca 33, 1135 Budapest, Hungary; darcsi.andras@nngyk.gov.hu

* Correspondence: moricz.agnes@atk.hun-ren.hu

Table of contents

No.	Legend	Page
**Figure S1.**	TLC chromatograms (**a**, **c**) visualized after derivatization with vanillin–sulfuric acid reagent (**a**) or *p*-anisaldehyde–sulfuric acid reagent (**c**), and TLC–DB bioautograms (**b, d**) obtained by a *B. subtilis* antibacterial assay of subfractions D (26-27/40-48/7-10) and E (37-44/37-40), containing compounds **4** (R_F 0.68) and **5** (R_F 0.58), respectively.	S-5
**Figure S2.**	^{1}H NMR spectrum of solidagolactone IX (**1**) (500 MHz, $CDCl_3$).	S-5
**Figure S3.**	^{13}C DEPTQ NMR spectrum of solidagolactone IX (**1**) (126 MHz, $CDCl_3$).	S-6
**Figure S4.**	^{1}H–^{1}H COSY NMR spectrum of solidagolactone IX (**1**) (500 MHz, $CDCl_3$).	S-6
**Figure S5.**	^{1}H–^{13}C edHSQC NMR spectrum of solidagolactone IX (**1**) (500/126 MHz, $CDCl_3$).	S-7
**Figure S6.**	^{1}H–^{13}C edHSQC NMR spectrum of solidagolactone IX (**1**) (500/126 MHz, $CDCl_3$) – aliphatic region (δ_C 40.0–14.0).	S-7
**Figure S7.**	^{1}H–^{13}C HMBC NMR spectrum of solidagolactone IX (**1**) (500/126 MHz, $CDCl_3$).	S-8
**Figure S8.**	^{1}H–^{1}H TOCSY NMR spectrum of solidagolactone IX (**1**) (500 MHz, $CDCl_3$).	S-8
**Figure S9.**	^{1}H–^{1}H ROESY NMR spectrum of solidagolactone IX (**1**) (500 MHz, $CDCl_3$).	S-9
**Figure S10.**	HR-ESI$^+$-MS spectrum of solidagolactone IX (**1**).	S-9
**Figure S11.**	HR-ESI$^+$-MS/MS of solidagolactone IX (**1**) with a normalized HCD collision energy of 50%.	S-10
**Figure S12.**	HR-ESI$^-$-MS spectrum of solidagolactone IX (**1**).	S-10
**Figure S13.**	HR-ESI$^-$-MS/MS of solidagolactone IX (**1**) with a normalized HCD collision energy of 20%.	S-10
**Figure S14.**	UV spectrum of solidagolactone IX (**1**) in ethanol.	S-11
**Figure S15.**	ATR-FTIR spectrum of solidagolactone IX (**1**).	S-11
**Figure S16.**	^{1}H NMR spectrum of solidagoic acid K (**2**) (500 MHz, $CDCl_3$).	S-12
**Figure S17.**	^{13}C DEPTQ NMR spectrum of solidagoic acid K (**2**) (126 MHz, $CDCl_3$).	S-12
**Figure S18.**	^{1}H–^{1}H COSY NMR spectrum of solidagoic acid K (**2**) (500 MHz, $CDCl_3$).	S-13
**Figure S19.**	^{1}H–^{13}C edHSQC NMR spectrum of solidagoic acid K (**2**) (500/126 MHz, $CDCl_3$).	S-13
**Figure S20.**	^{1}H–^{13}C edHSQC NMR spectrum of solidagoic acid K (**2**) (500/126 MHz, $CDCl_3$) – aliphatic region (δ_C 44.0–14.0).	S-14
**Figure S21.**	^{1}H–^{13}C HMBC NMR spectrum of solidagoic acid K (**2**) (500/126 MHz, $CDCl_3$).	S-14
**Figure S22.**	^{1}H–^{1}H TOCSY NMR spectrum of solidagoic acid K (**2**) (500 MHz, $CDCl_3$).	S-15
**Figure S23.**	^{1}H–^{1}H ROESY NMR spectrum of solidagoic acid K (**2**) (500 MHz, $CDCl_3$).	S-15
**Figure S24.**	HR-ESI$^+$-MS spectrum of solidagoic acid K (**2**).	S-16

**Figure S25.**	HR-ESI+-MS/MS spectrum of solidagoic acid K (**2**) with a normalized HCD collision energy of 30%.	S-16
**Figure S26.**	HR-ESI−-MS spectrum of solidagoic acid K (**2**).	S-16
**Figure S27.**	HR-ESI−-MS/MS spectrum of solidagoic acid K (**2**) with a normalized HCD collision energy of 30%.	S-17
**Figure S28.**	UV spectrum of solidagoic acid K (**2**) in ethanol.	S-17
**Figure S29.**	ATR-FTIR spectrum of solidagoic acid K (**2**).	S-18
**Figure S30.**	^{1}H NMR spectrum of solidagodiol (**3**) (500 MHz, $CDCl_3$).	S-18
**Figure S31.**	^{13}C DEPTQ NMR spectrum of solidagodiol (**3**) (126 MHz, $CDCl_3$).	S-19
**Figure S32.**	^{1}H–^{1}H COSY NMR spectrum of solidagodiol (**3**) (500 MHz, $CDCl_3$).	S-19
**Figure S33.**	^{1}H–^{13}C edHSQC NMR spectrum of solidagodiol (**3**) (500/126 MHz, $CDCl_3$).	S-20
**Figure S34.**	^{1}H–^{13}C HMBC NMR spectrum of solidagodiol (**3**) (500/126 MHz, $CDCl_3$).	S-20
**Figure S35.**	^{1}H–^{1}H TOCSY NMR spectrum of solidagodiol (**3**) (500 MHz, $CDCl_3$).	S-21
**Figure S36.**	^{1}H–^{1}H ROESY NMR spectrum of solidagodiol (**3**) (500 MHz, $CDCl_3$).	S-21
**Figure S37.**	HR-ESI+-MS spectrum of solidagodiol (**3**).	S-22
**Figure S38.**	HR-ESI+-MS/MS spectrum of solidagodiol (**3**) with a normalized HCD collision energy of 25%.	S-22
**Figure S39.**	UV spectrum of solidagodiol (**3**) in ethanol.	S-23
**Figure S40.**	ATR-FTIR spectrum of solidagodiol (**3**).	S-23
**Figure S41.**	^{1}H NMR spectrum of (–)-(5*R*,8*R*,9*R*,10*S*)-15,16-epoxy-*ent-neo*-cleroda-3,13,14-trien-18-ol (**4**) (500 MHz, $CDCl_3$).	S-24
**Figure S42.**	^{13}C DEPTQ NMR spectrum of (–)-(5*R*,8*R*,9*R*,10*S*)-15,16-epoxy-*ent-neo*-cleroda-3,13,14-trien-18-ol (**4**) (126 MHz, $CDCl_3$).	S-24
**Figure S43.**	^{1}H–^{1}H COSY NMR spectrum of (–)-(5*R*,8*R*,9*R*,10*S*)-15,16-epoxy-*ent-neo*-cleroda-3,13,14-trien-18-ol (**4**) (500 MHz, $CDCl_3$).	S-25
**Figure S44.**	^{1}H–^{13}C edHSQC NMR spectrum of (–)-(5*R*,8*R*,9*R*,10*S*)-15,16-epoxy-*ent-neo*-cleroda-3,13,14-trien-18-ol (**4**) (500/126 MHz, $CDCl_3$).	S-25
**Figure S45.**	^{1}H–^{13}C HMBC NMR spectrum of (–)-(5*R*,8*R*,9*R*,10*S*)-15,16-epoxy-*ent-neo*-cleroda-3,13,14-trien-18-ol (**4**) (500/126 MHz, $CDCl_3$).	S-26
**Figure S46.**	^{1}H–^{1}H TOCSY NMR spectrum of (–)-(5*R*,8*R*,9*R*,10*S*)-15,16-epoxy-*ent-neo*-cleroda-3,13,14-trien-18-ol (**4**) (500 MHz, $CDCl_3$).	S-26
**Figure S47.**	^{1}H–^{1}H ROESY NMR spectrum of (–)-(5*R*,8*R*,9*R*,10*S*)-15,16-epoxy-*ent-neo*-cleroda-3,13,14-trien-18-ol (**4**) (500 MHz, $CDCl_3$).	S-27
**Figure S48.**	HR-ESI+-MS spectrum of (–)-(5*R*,8*R*,9*R*,10*S*)-15,16-epoxy-*ent-neo*-cleroda-3,13,14-trien-18-ol (**4**).	S-27
**Figure S49.**	^{1}H NMR spectrum of solidagoic acid J (**5**) (500 MHz, $CDCl_3$).	S-28
**Figure S50.**	^{13}C DEPTQ NMR spectrum of solidagoic acid J (**5**) (126 MHz, $CDCl_3$).	S-28
**Figure S51.**	^{1}H–^{1}H COSY NMR spectrum of solidagoic acid J (**5**) (500 MHz, $CDCl_3$).	S-29
**Figure S52.**	^{1}H–^{13}C edHSQC NMR spectrum of solidagoic acid J (**5**) (500/126 MHz, $CDCl_3$).	S-29
**Figure S53.**	^{1}H–^{13}C HMBC NMR spectrum of solidagoic acid J (**5**) (500/126 MHz, $CDCl_3$).	S-30

**Figure S54.**	^1H–^1H TOCSY NMR spectrum of solidagoic acid J (**5**) (500 MHz, $CDCl_3$).	S-30
**Figure S55.**	^1H–^1H ROESY NMR spectrum of solidagoic acid J (**5**) (500 MHz, $CDCl_3$).	S-31
**Figure S56.**	HR-ESI$^+$-MS spectrum of solidagoic acid J (**5**).	S-31
**Figure S57.**	HR-ESI$^+$-MS/MS spectrum of solidagoic acid J (**5**) with a normalized HCD collision energy of 40%.	S-32
**Figure S58.**	HR-ESI$^-$-MS spectrum of solidagoic acid J (**5**).	S-32
**Figure S59.**	HR-ESI$^-$-MS/MS spectrum of solidagoic acid J (**5**) with a normalized HCD collision energy of 15%.	S-32

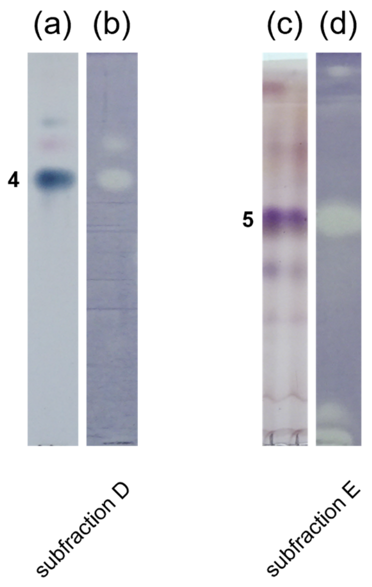

Figure S1. TLC chromatograms (**a**, **c**) visualized after derivatization with vanillin–sulfuric acid reagent (**a**) or *p*-anisaldehyde–sulfuric acid reagent (**c**), and TLC–DB bioautograms (**b, d**) obtained by a *B. subtilis* antibacterial assay of subfractions D (26-27/40-48/7-10) and E (37-44/37-40), containing compounds **4** (R_F 0.68) and **5** (R_F 0.58), respectively.

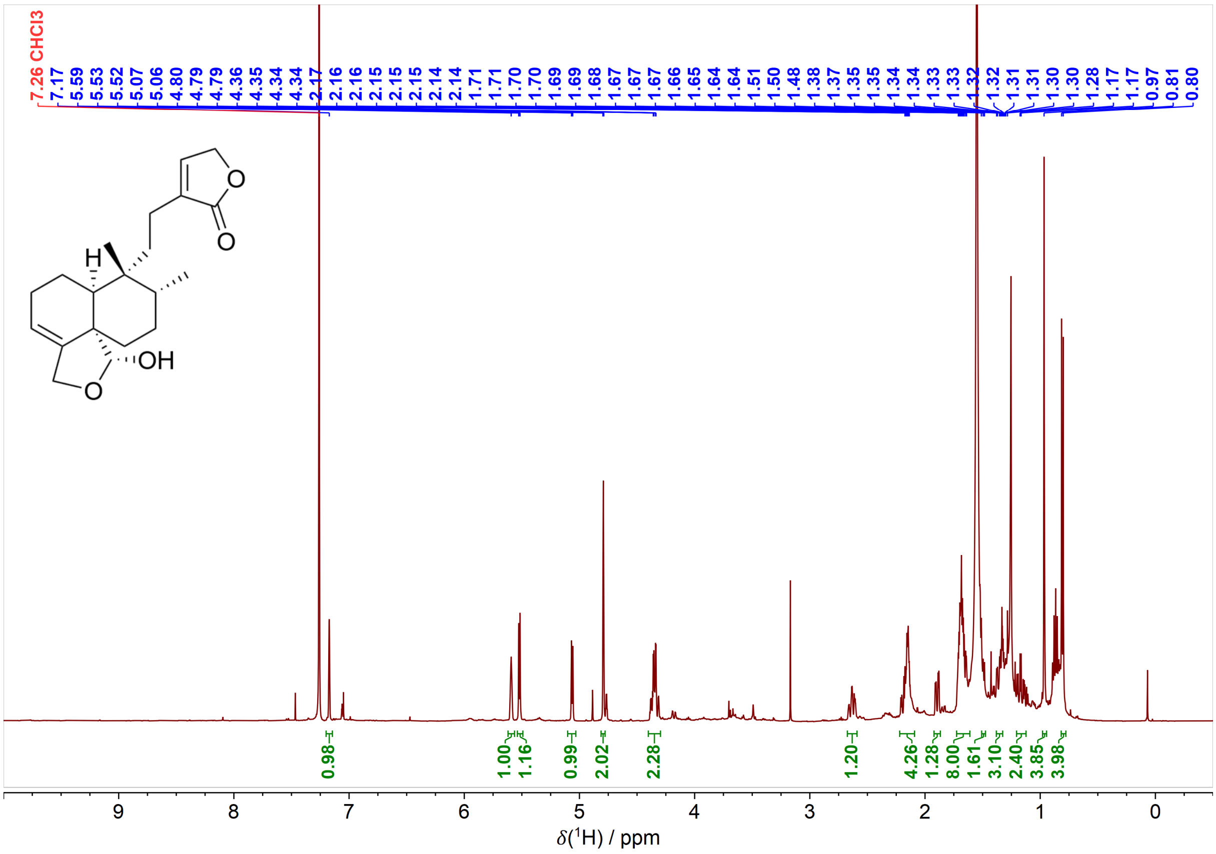

Figure S2. ^{1}H NMR spectrum of solidagolactone IX (**1**) (500 MHz, $CDCl_3$).

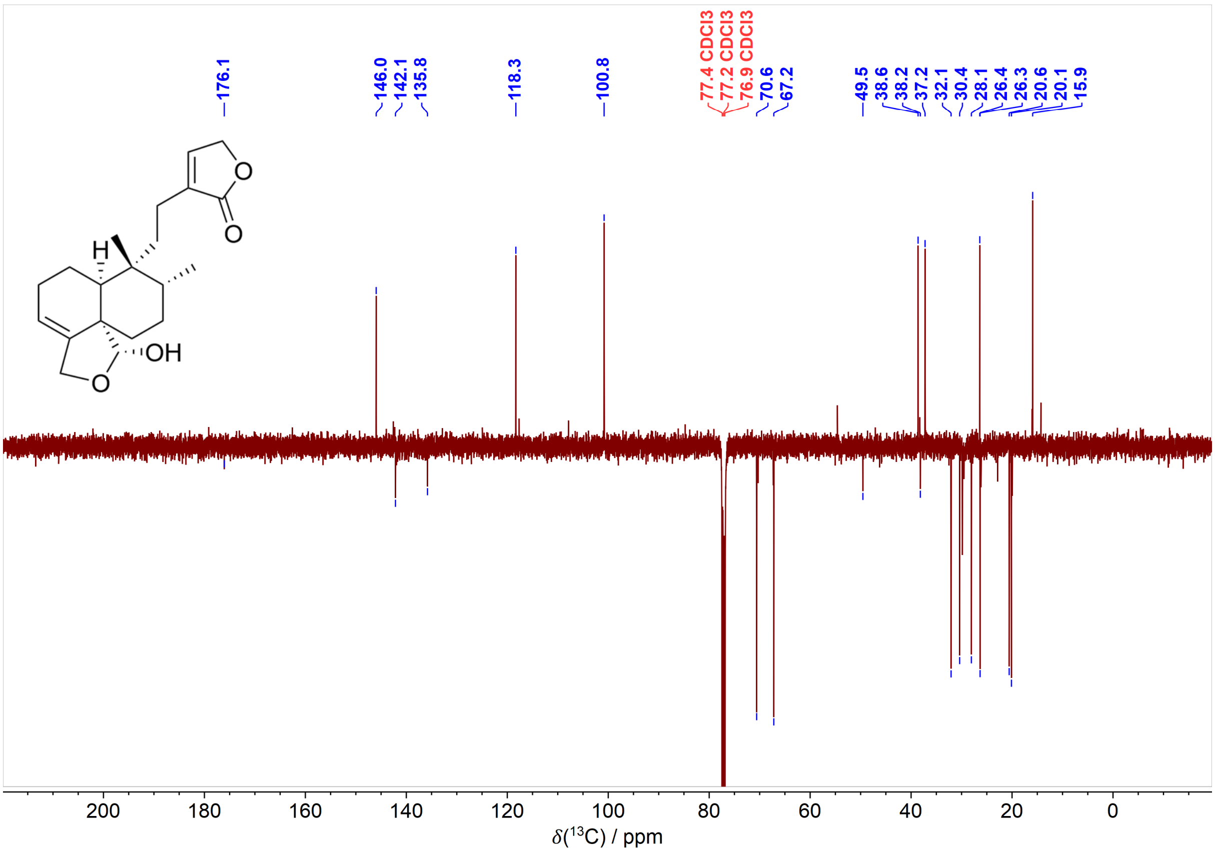

Figure S3. ^{13}C DEPTQ NMR spectrum of solidagolactone IX (**1**) (126 MHz, $CDCl_3$).

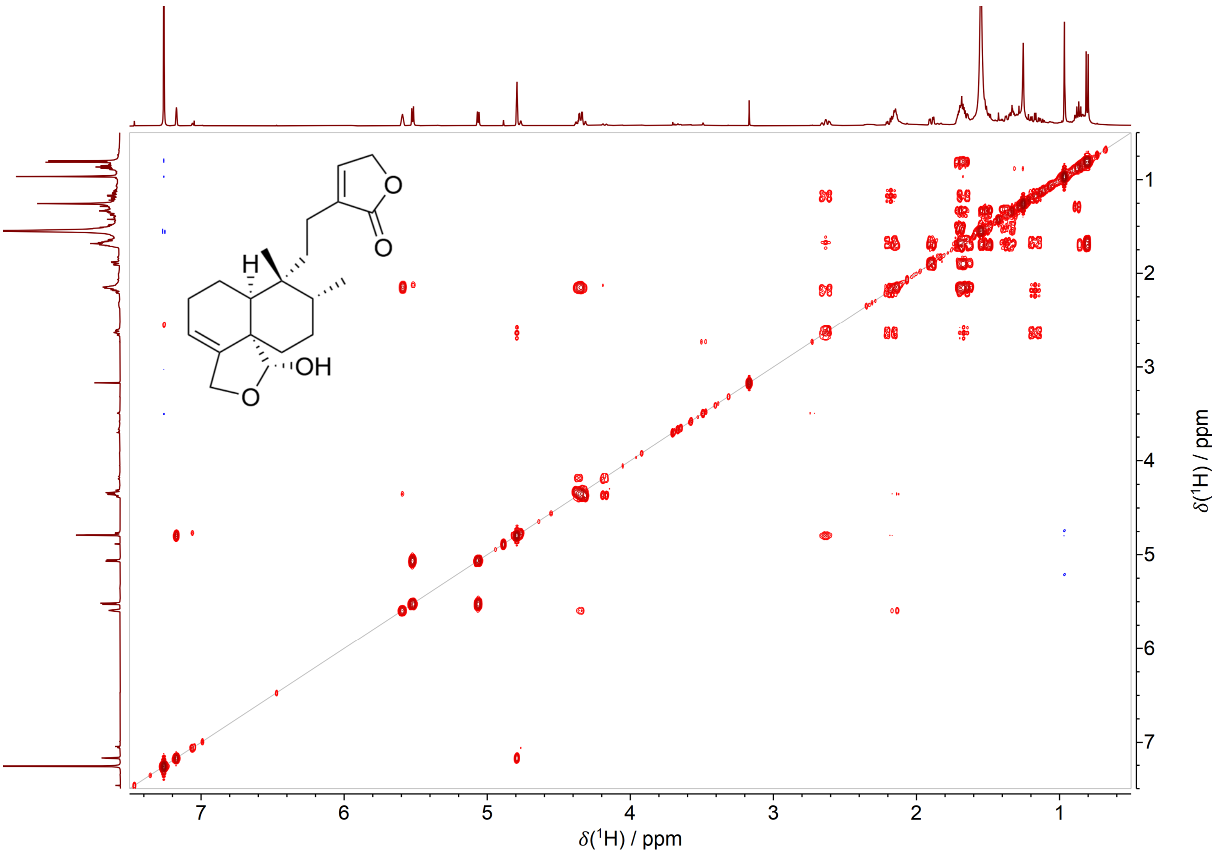

Figure S4. ^{1}H–^{1}H COSY NMR spectrum of solidagolactone IX (**1**) (500 MHz, $CDCl_3$).

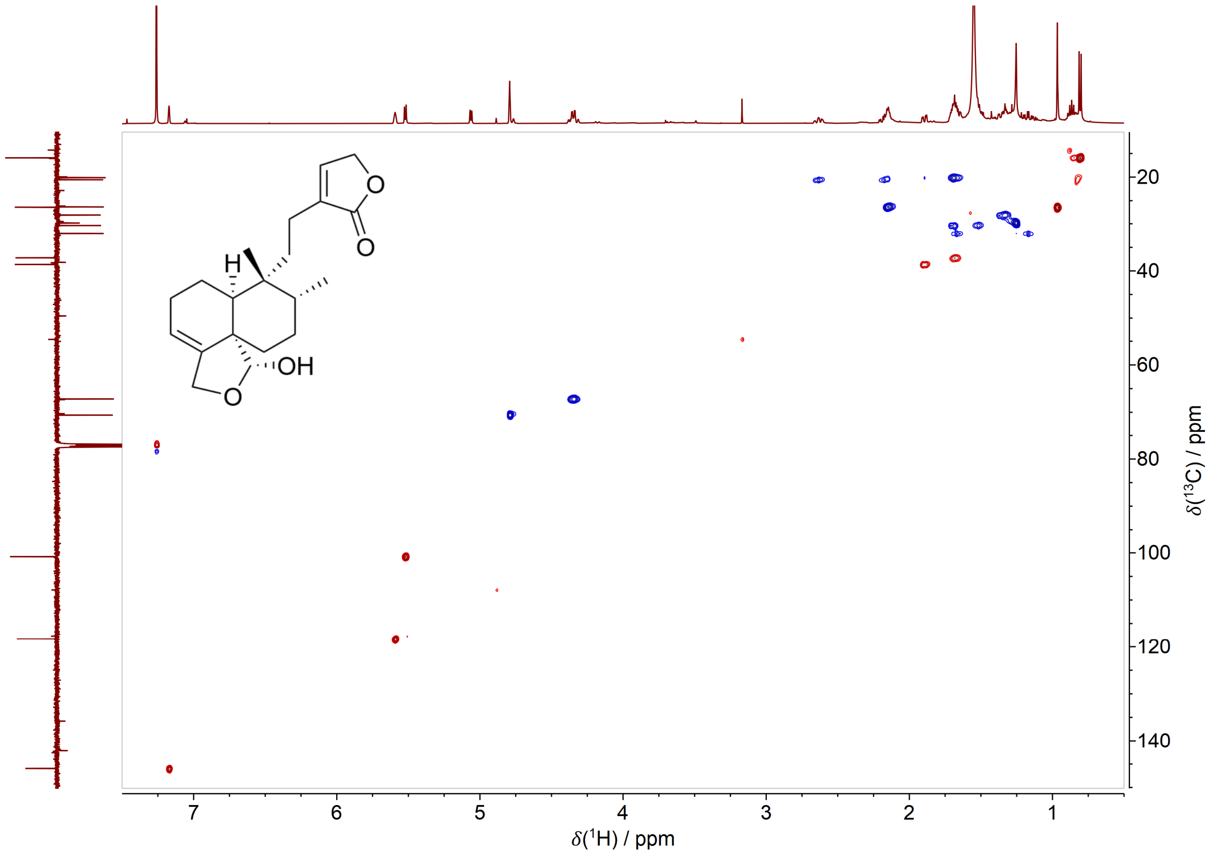

Figure S5. ^{1}H–^{13}C edHSQC NMR spectrum of solidagolactone IX (**1**) (500/126 MHz, CDCl$_3$).

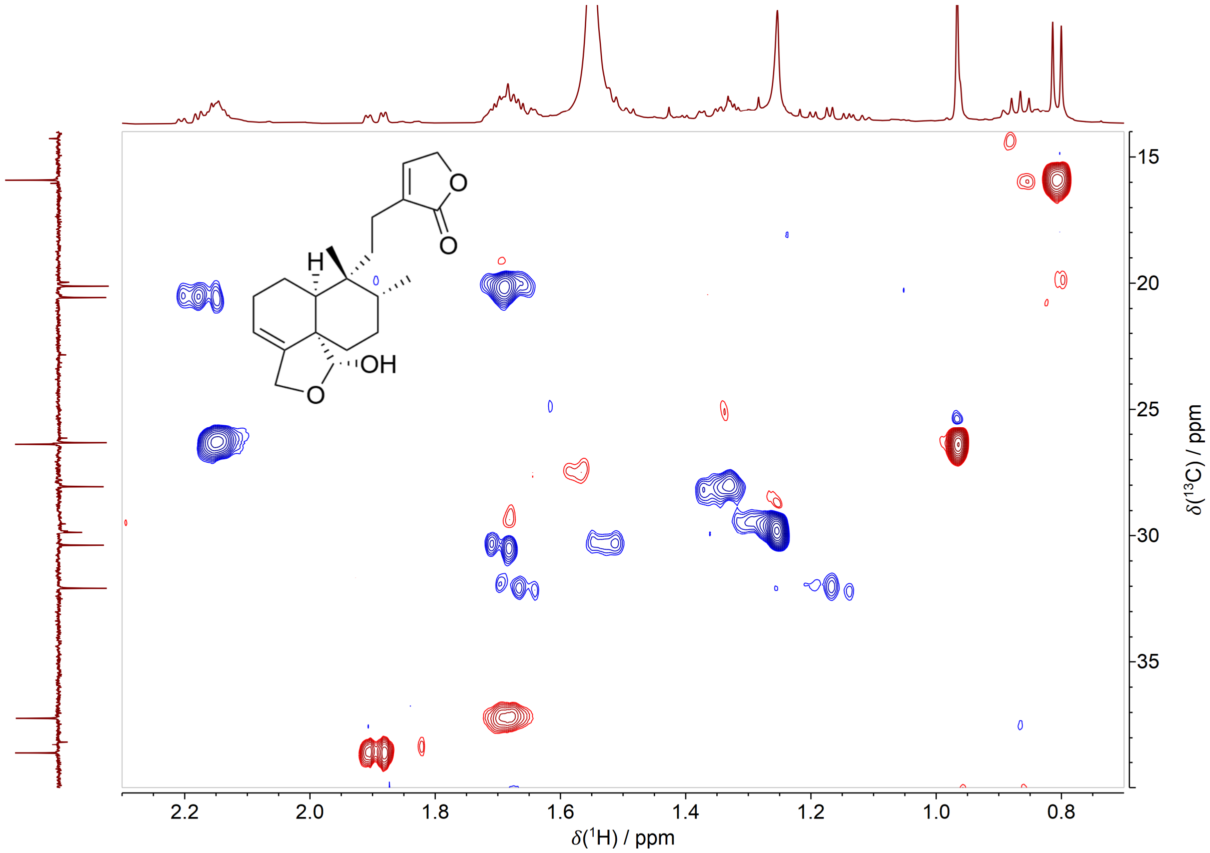

Figure S6. ^{1}H–^{13}C edHSQC NMR spectrum of solidagolactone IX (**1**) (500/126 MHz, CDCl$_3$) – aliphatic region (δ_C 40.0–14.0).

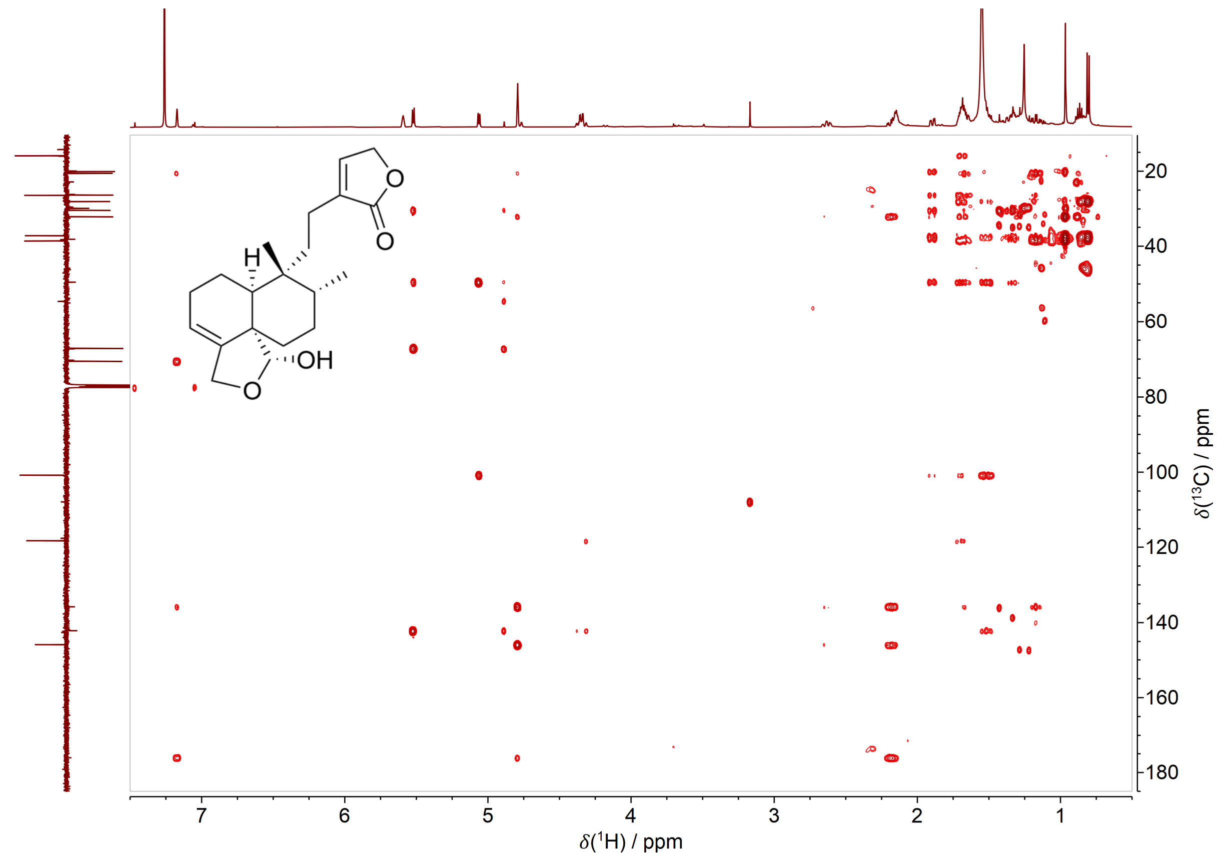

Figure S7. ^{1}H–^{13}C HMBC NMR spectrum of solidagolactone IX (**1**) (500/126 MHz, $CDCl_3$).

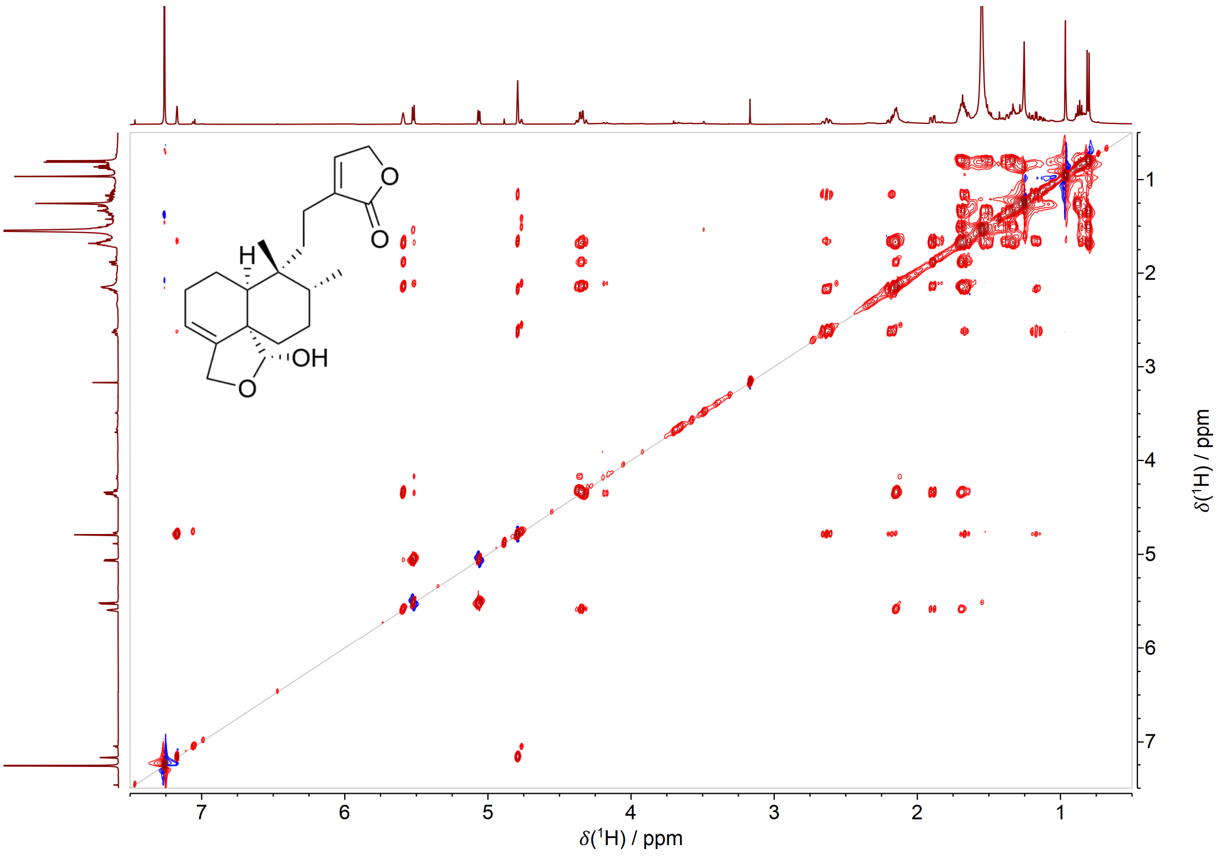

Figure S8. ^{1}H–^{1}H TOCSY NMR spectrum of solidagolactone IX (**1**) (500 MHz, $CDCl_3$).

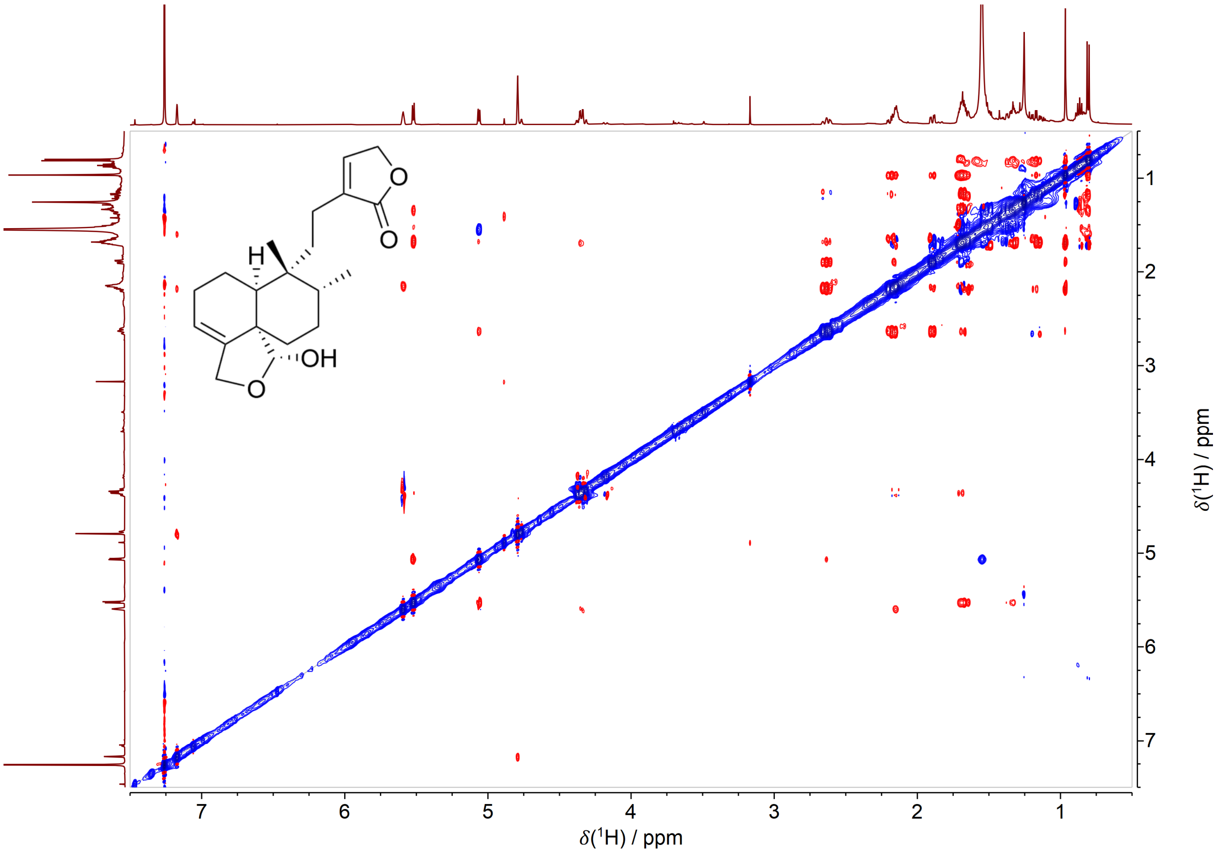

Figure S9. ^{1}H–^{1}H ROESY NMR spectrum of solidagolactone IX (**1**) (500 MHz, $CDCl_3$).

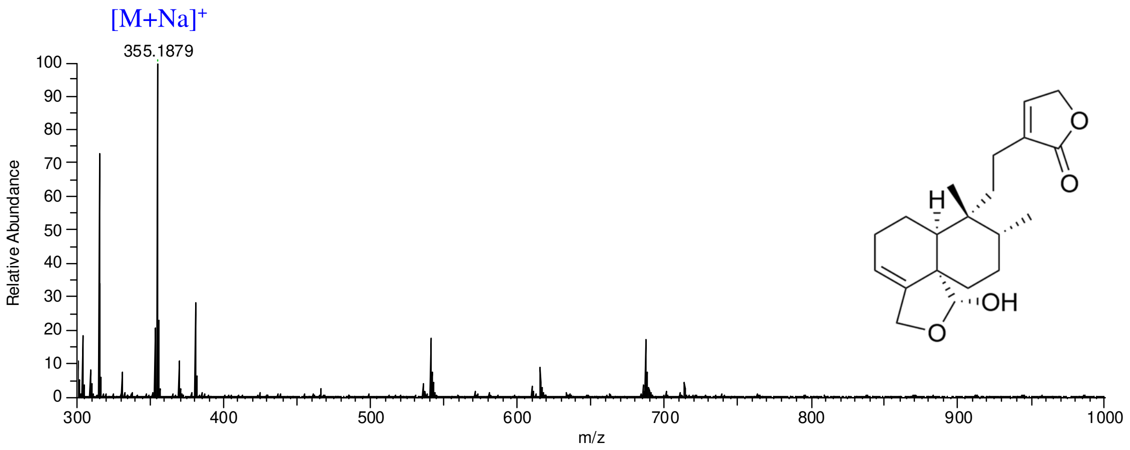

Figure S10. HR-ESI^{+}-MS spectrum of solidagolactone IX (**1**), *m/z* 355.1879 $[M+Na]^+$ (calculated for $C_{20}H_{28}O_4Na^+$, *m/z* 355.1880 $[M+Na]^+$, error: –0.3 ppm).

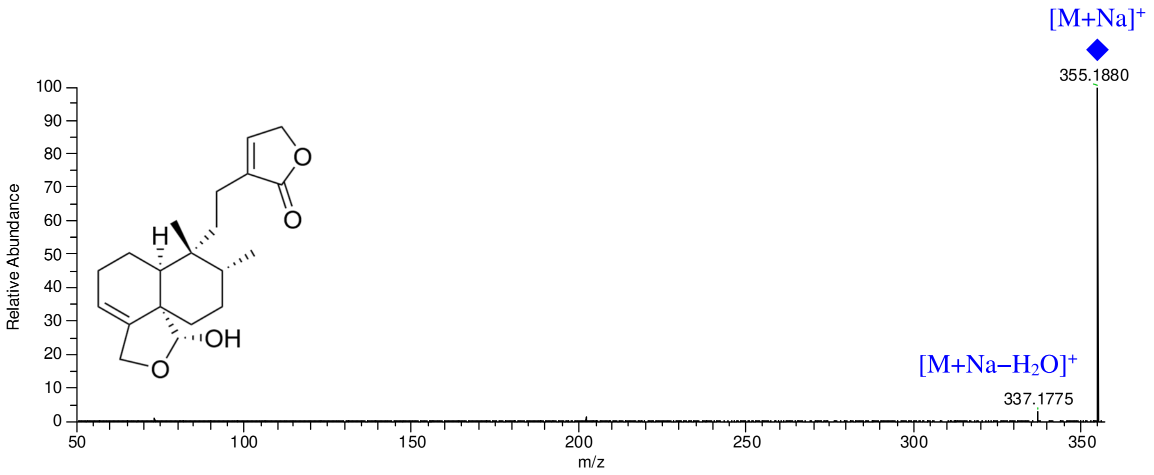

Figure S11. HR-ESI$^+$-MS/MS spectrum of solidagolactone IX (**1**) with a normalized HCD collision energy of 50%. Precursor ion: *m/z* 355.1880 [M+Na]$^+$, $C_{20}H_{28}O_4Na^+$.

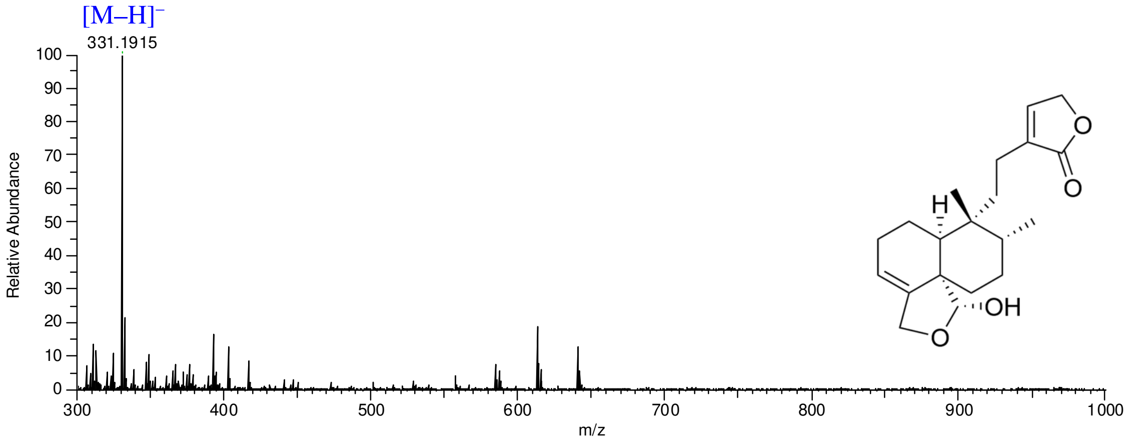

Figure S12. HR-ESI$^-$-MS spectrum of solidagolactone IX (**1**), *m/z* 331.1915 [M–H]$^-$ (calculated for $C_{20}H_{27}O_4^-$, *m/z* 331.1915 [M–H]$^-$, error: 0.1 ppm).

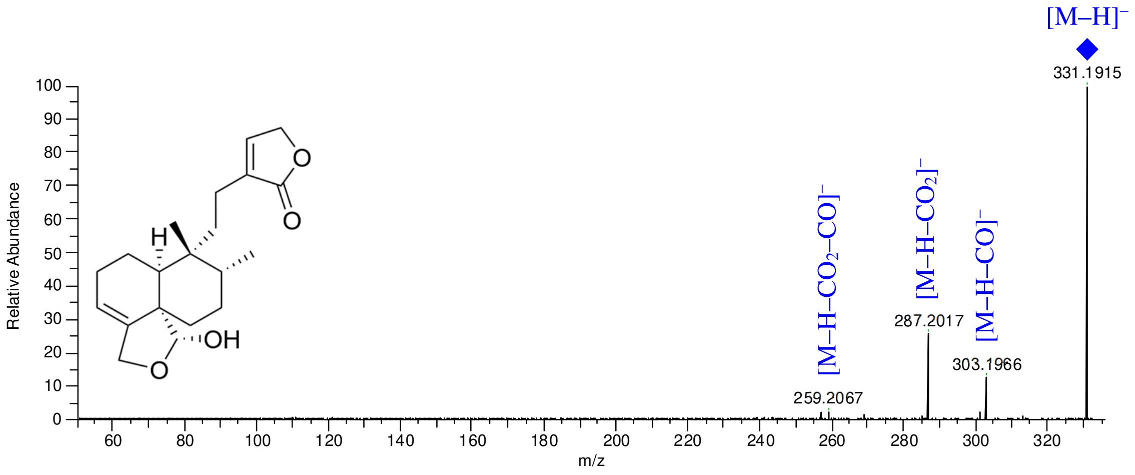

Figure S13. HR-ESI$^-$-MS/MS spectrum of solidagolactone IX (**1**) with a normalized HCD collision energy of 20%. Precursor ion: *m/z* 331.1915 [M–H]$^-$, $C_{20}H_{27}O_4^-$.

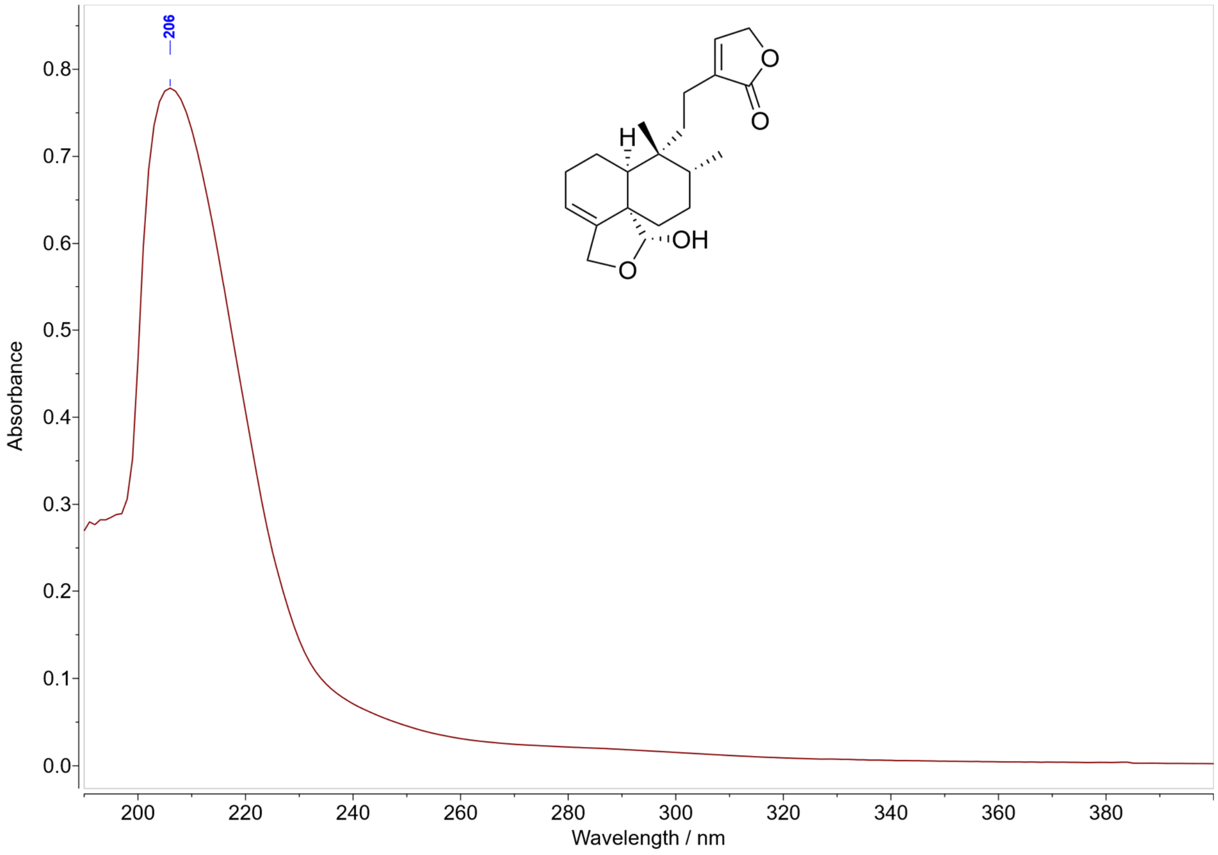

Figure S14. UV spectrum of solidagolactone IX (**1**) in ethanol.

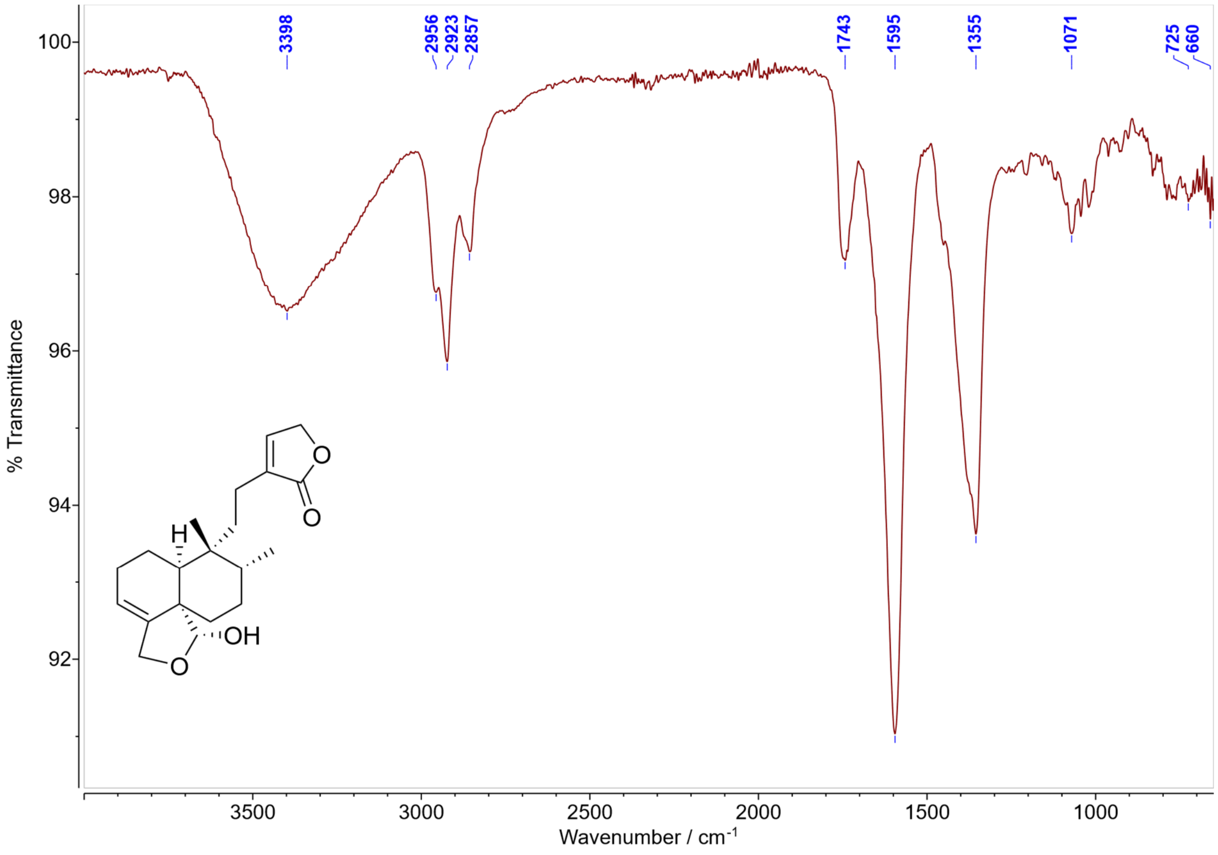

Figure S15. ATR-FTIR spectrum of solidagolactone IX (**1**).

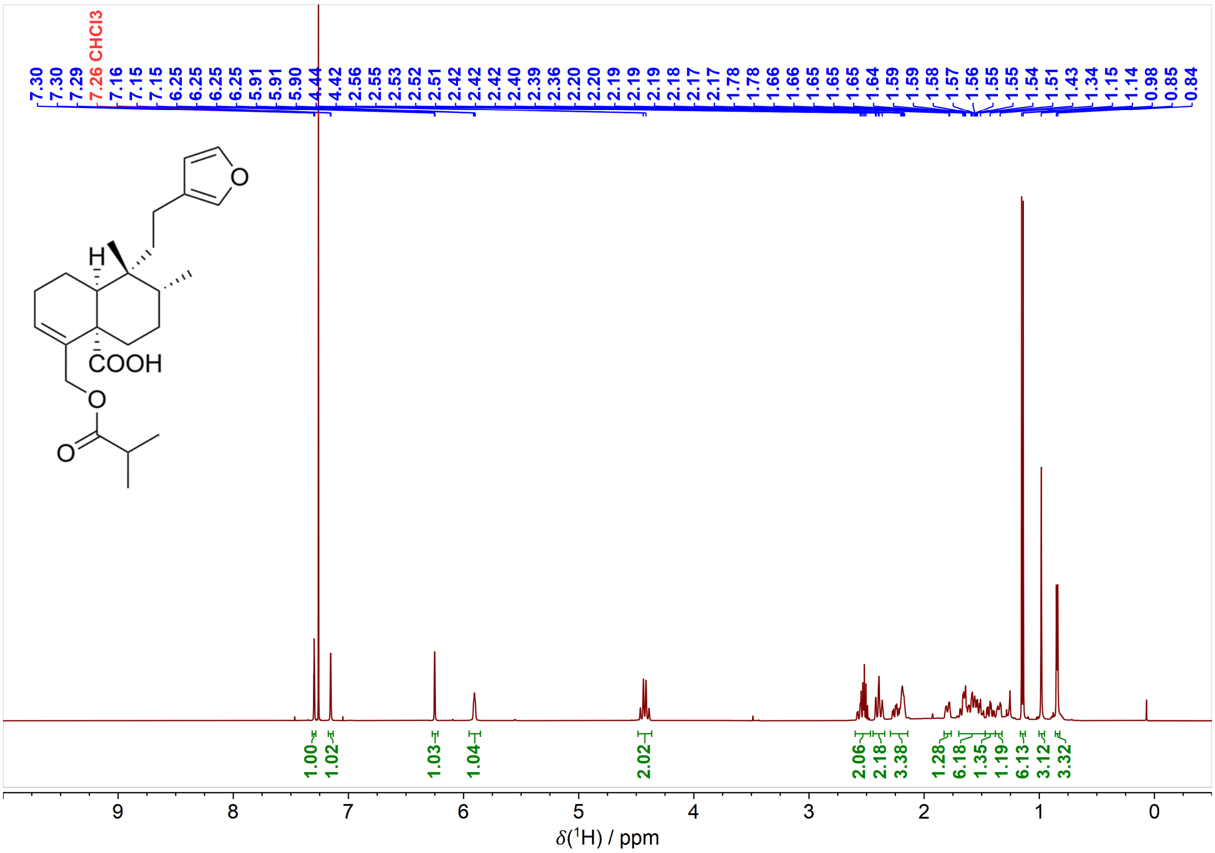

Figure S16. ^{1}H NMR spectrum of solidagoic acid K (**2**) (500 MHz, $CDCl_3$).

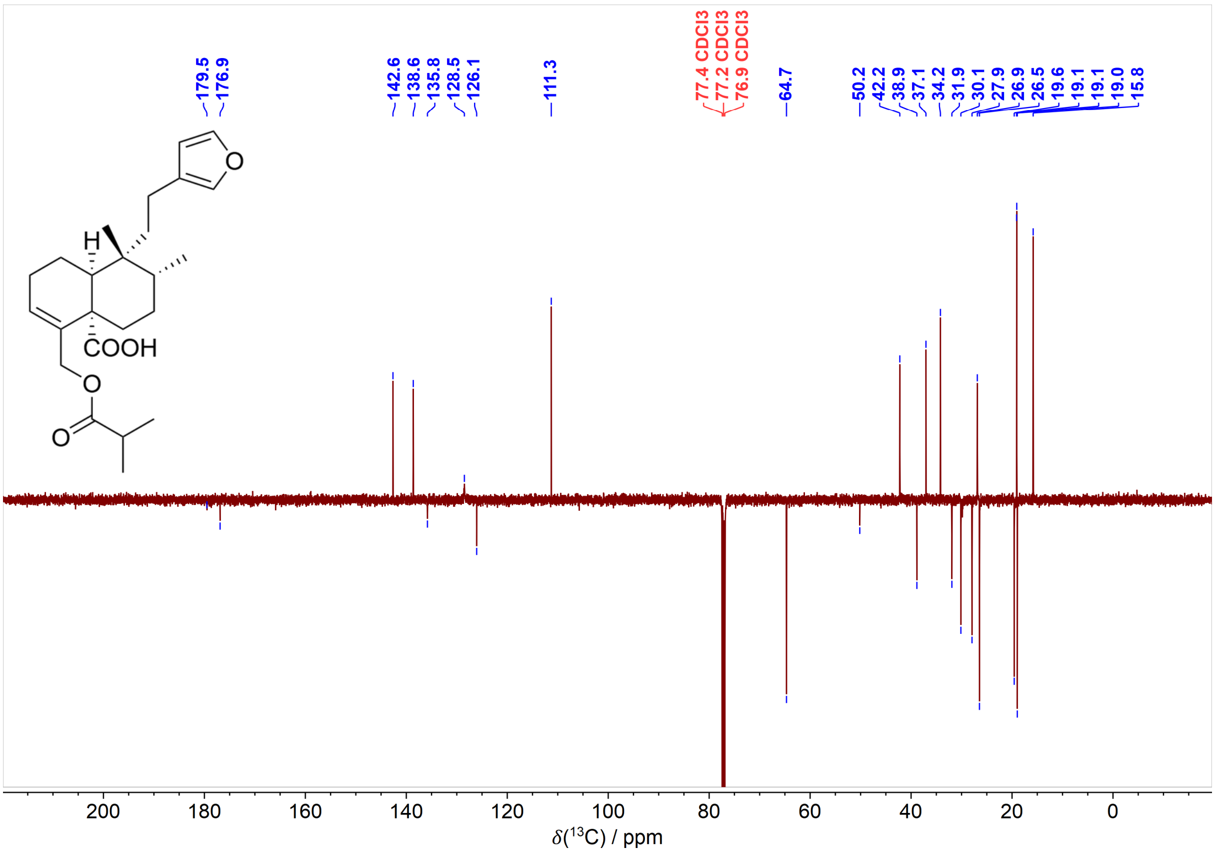

Figure S17. ^{13}C DEPTQ NMR spectrum of solidagoic acid K (**2**) (126 MHz, $CDCl_3$).

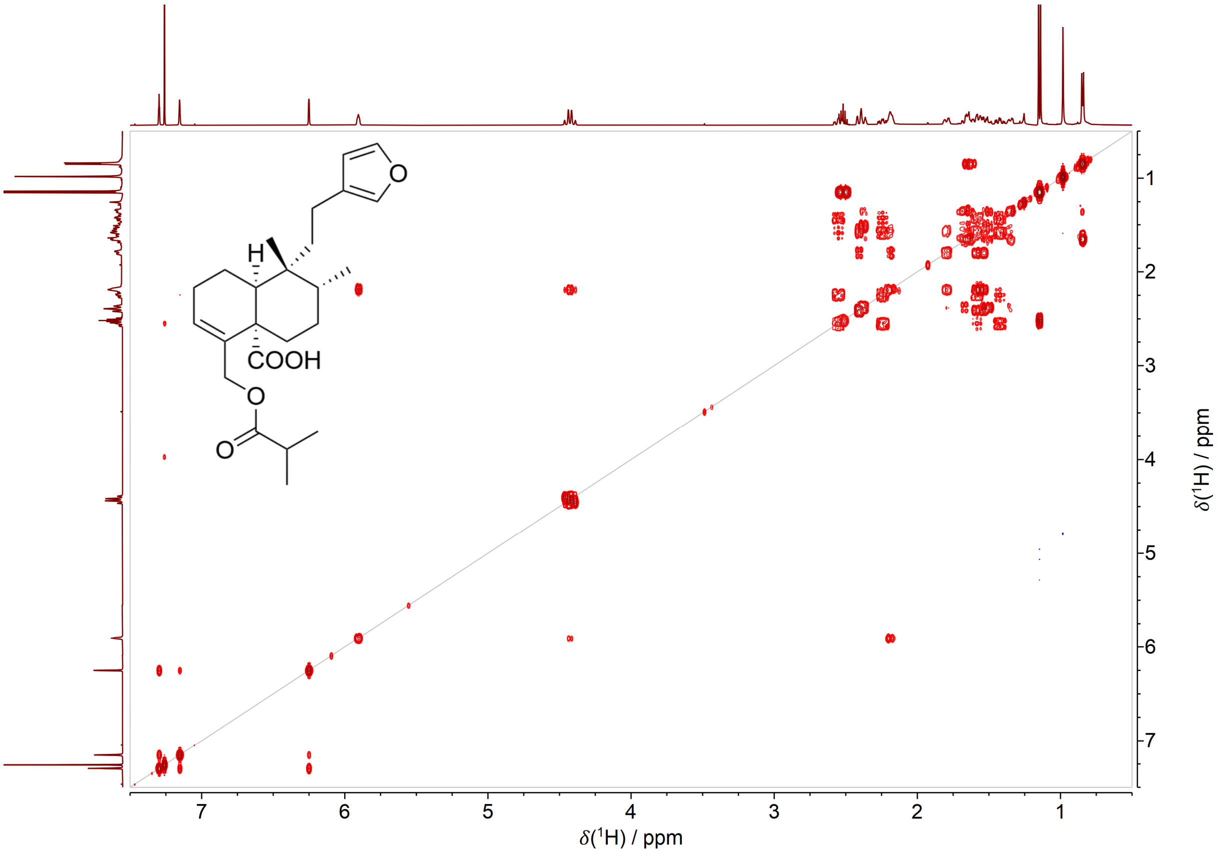

Figure S18. ^{1}H–^{1}H COSY NMR spectrum of solidagoic acid K (**2**) (500 MHz, $CDCl_3$).

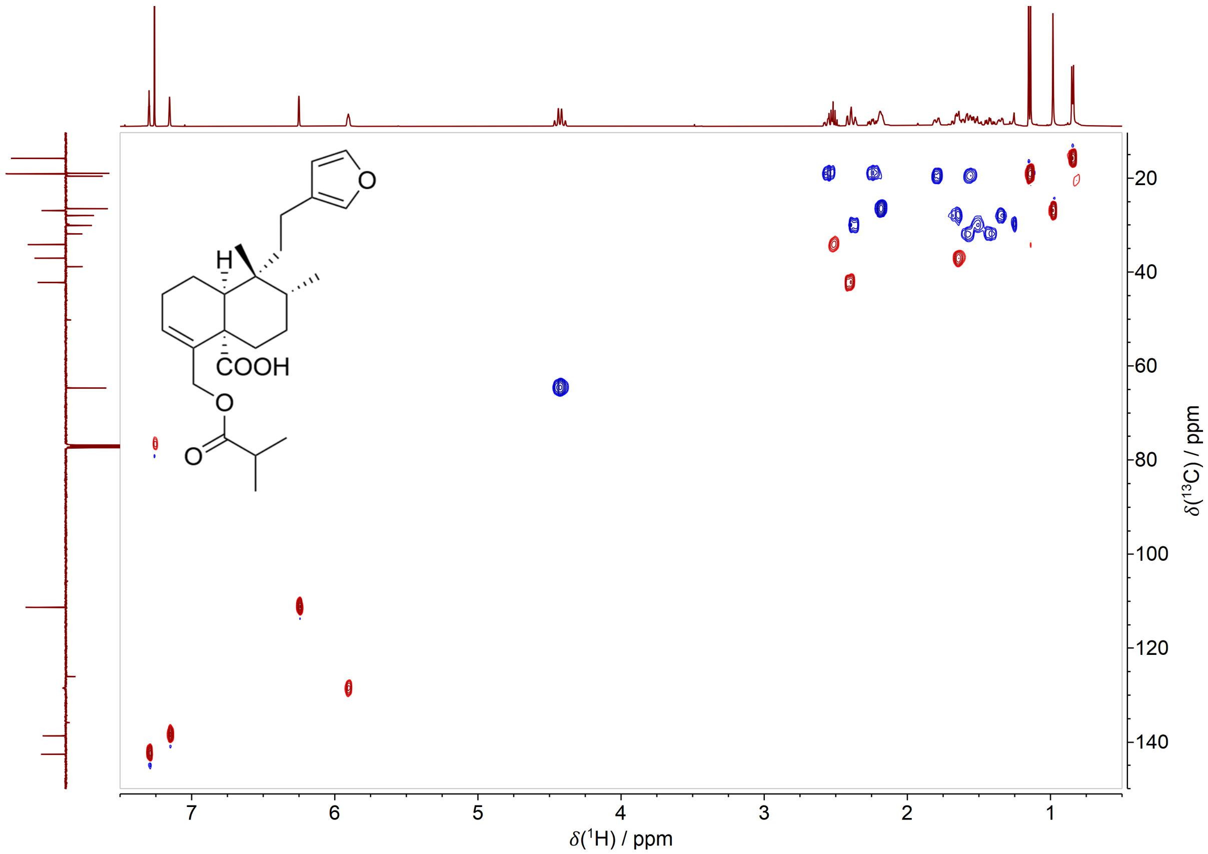

Figure S19. ^{1}H–^{13}C edHSQC NMR spectrum of solidagoic acid K (**2**) (500/126 MHz, $CDCl_3$).

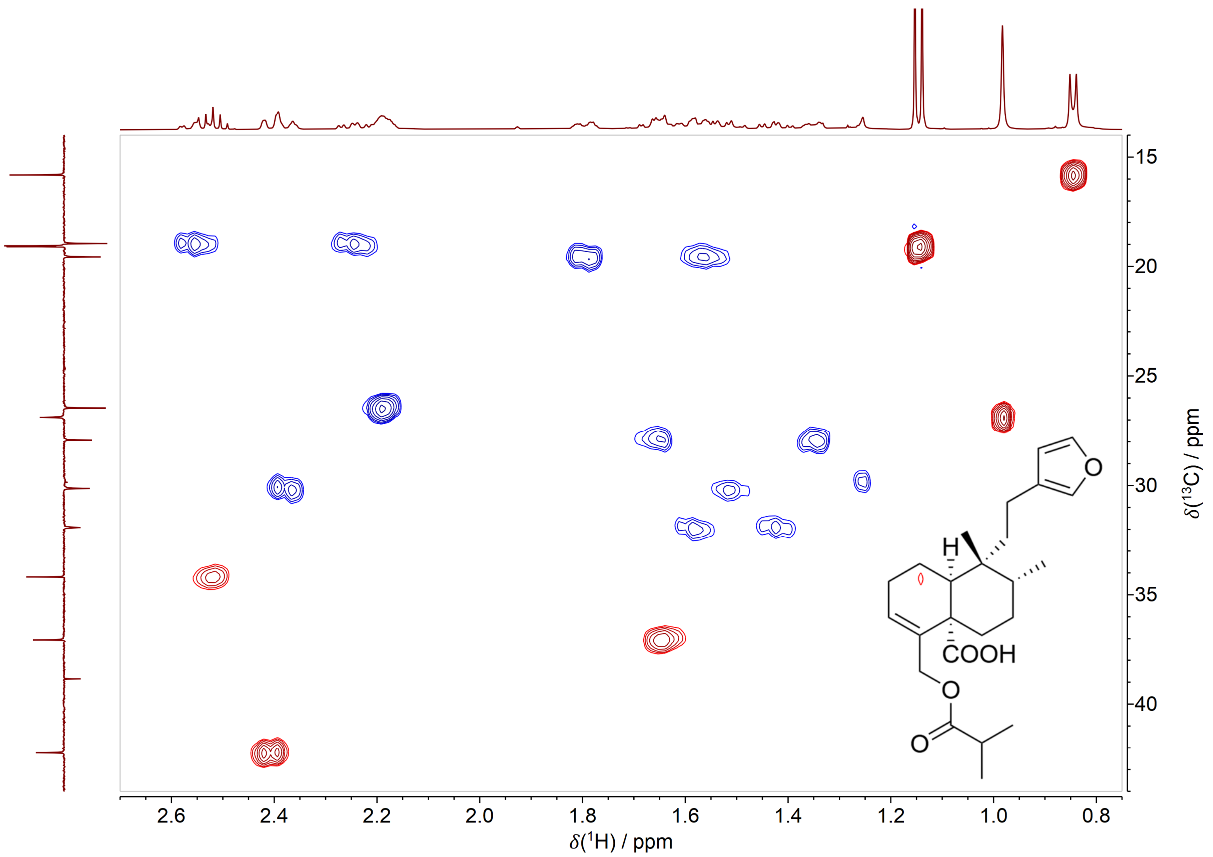

Figure S20. ^{1}H–^{13}C edHSQC NMR spectrum of solidagoic acid K (**2**) (500/126 MHz, $CDCl_3$) – aliphatic region (δ_C 44.0–14.0).

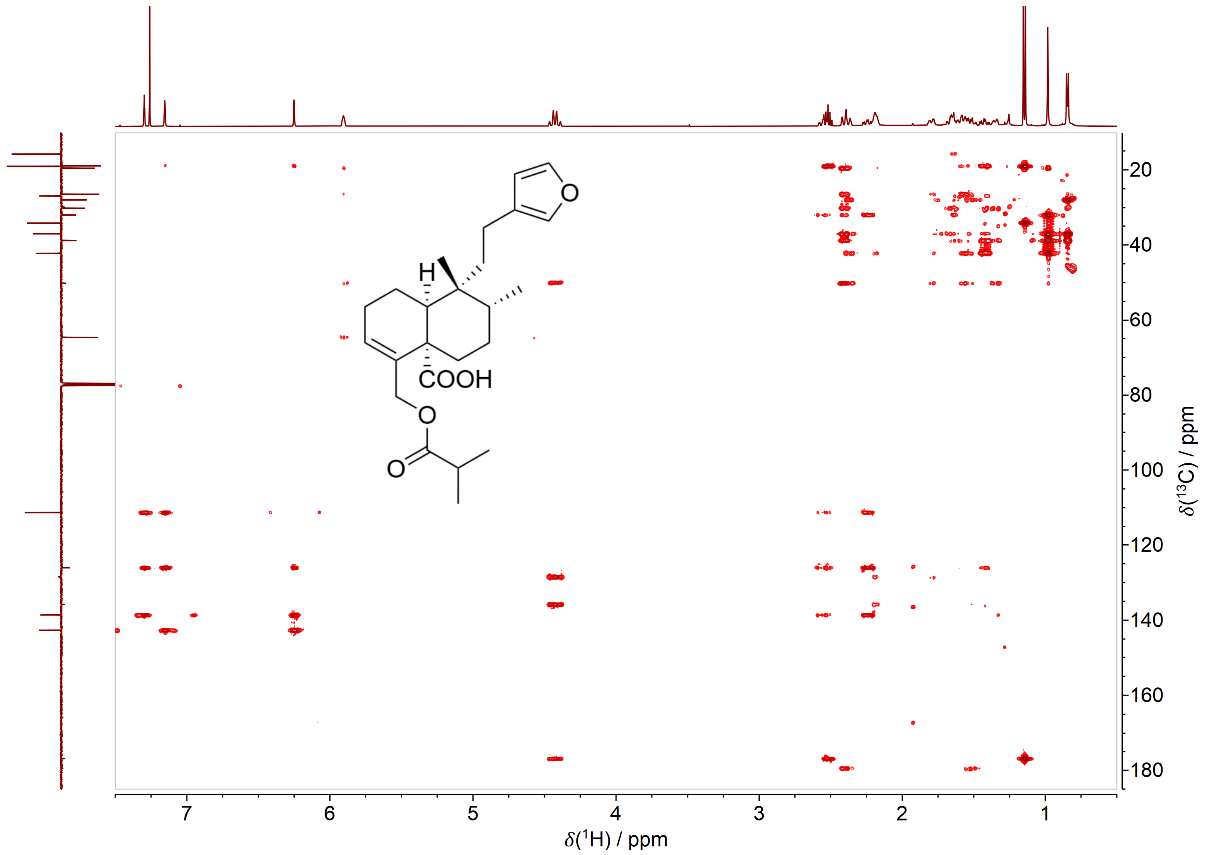

Figure S21. ^{1}H–^{13}C HMBC NMR spectrum of solidagoic acid K (**2**) (500/126 MHz, $CDCl_3$).

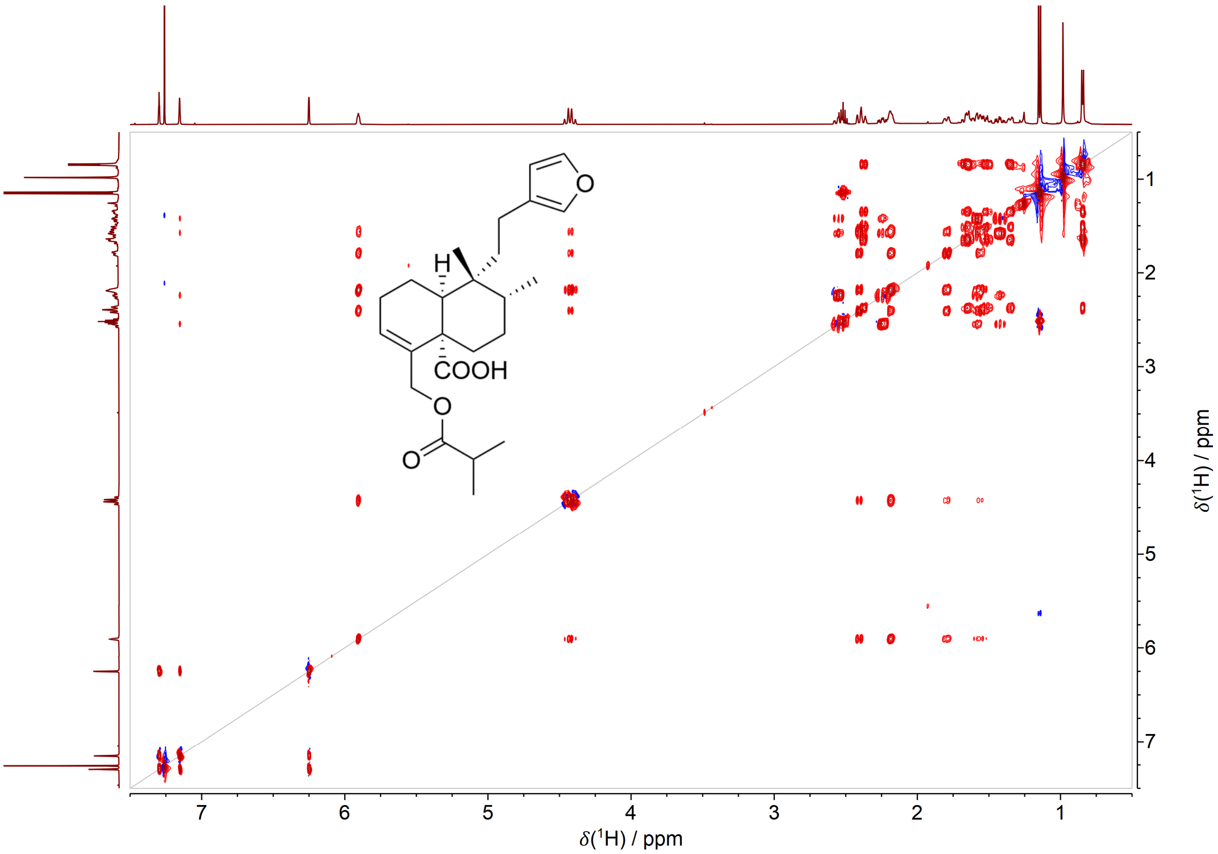

Figure S22. ^{1}H–^{1}H TOCSY NMR spectrum of solidagoic acid K (**2**) (500 MHz, $CDCl_3$).

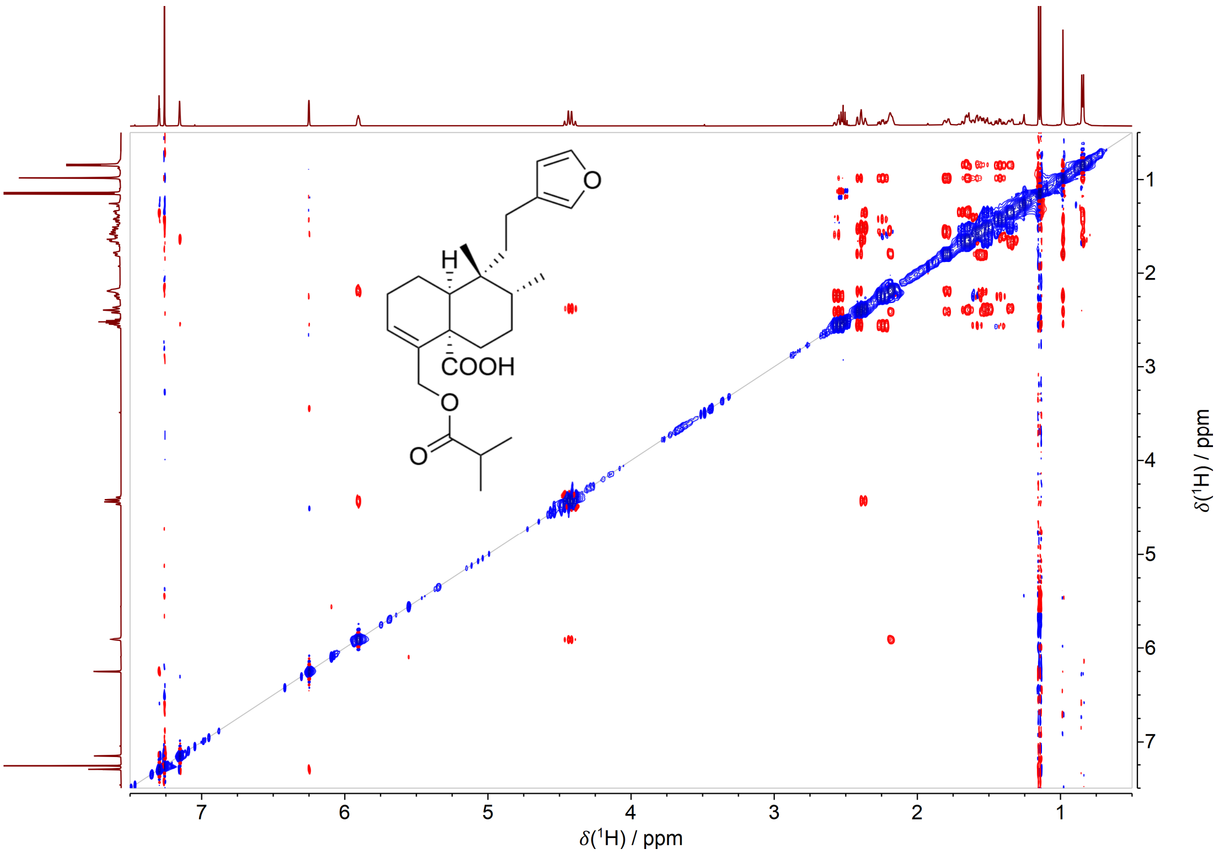

Figure S23. ^{1}H–^{1}H ROESY NMR spectrum of solidagoic acid K (**2**) (500 MHz, $CDCl_3$).

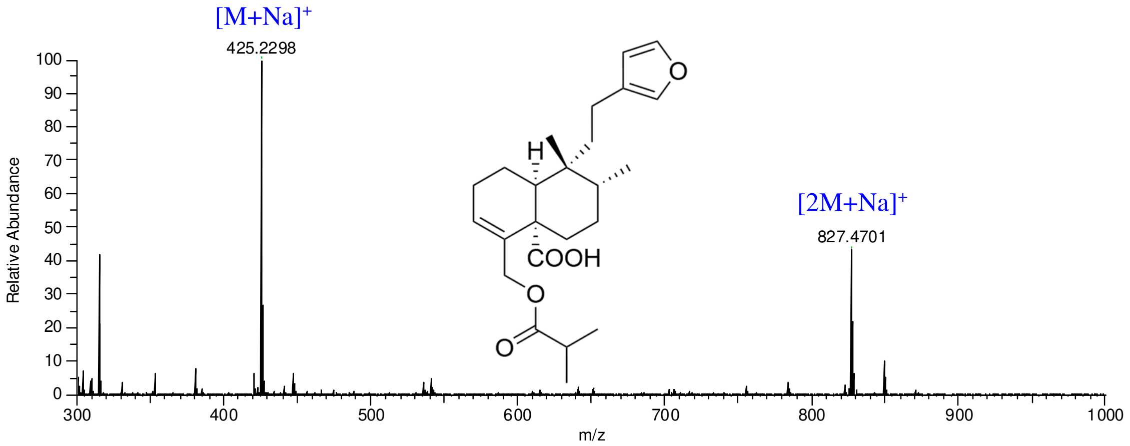

Figure S24. HR-ESI$^+$-MS spectrum of solidagoic acid K (**2**), *m/z* 425.2298 [M+Na]$^+$ (calculated for $C_{24}H_{34}O_5Na^+$, *m/z* 425.2299 [M+Na]$^+$, error: –0.1 ppm).

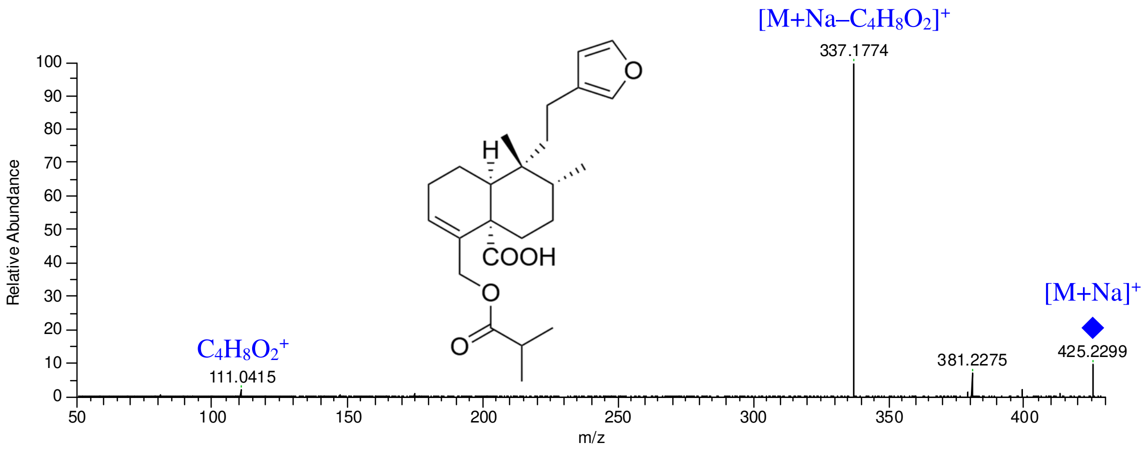

Figure S25. HR-ESI$^+$-MS/MS spectrum of solidagoic acid K (**2**) with a normalized HCD collision energy of 30%. Precursor ion: *m/z* 425.2299 [M+Na]$^+$, $C_{24}H_{34}O_5Na^+$.

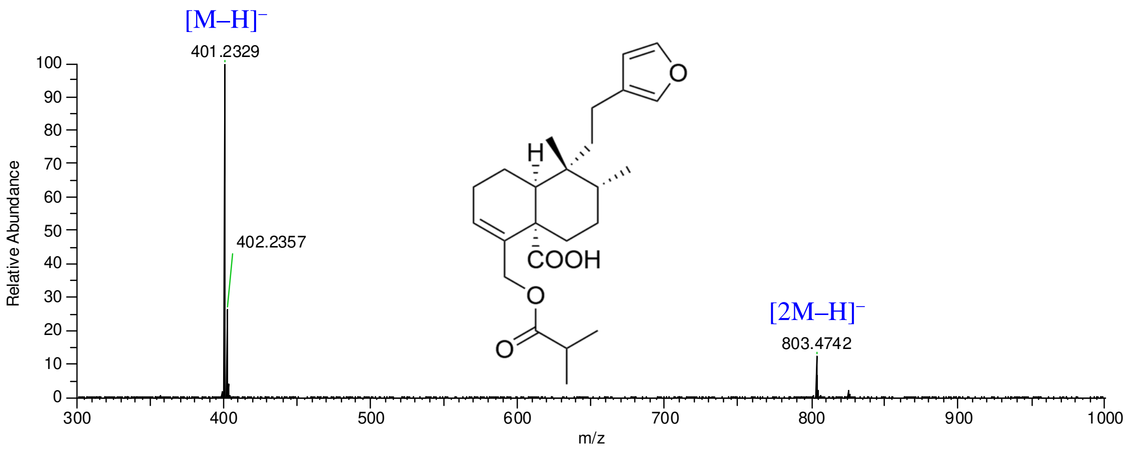

Figure S26. HR-ESI$^-$-MS spectrum of solidagoic acid K (**2**), *m/z* 401.2329 [M–H]$^-$ (calculated for $C_{24}H_{33}O_5^-$, *m/z* 401.2329 [M–H]$^-$, error: 0.1 ppm).

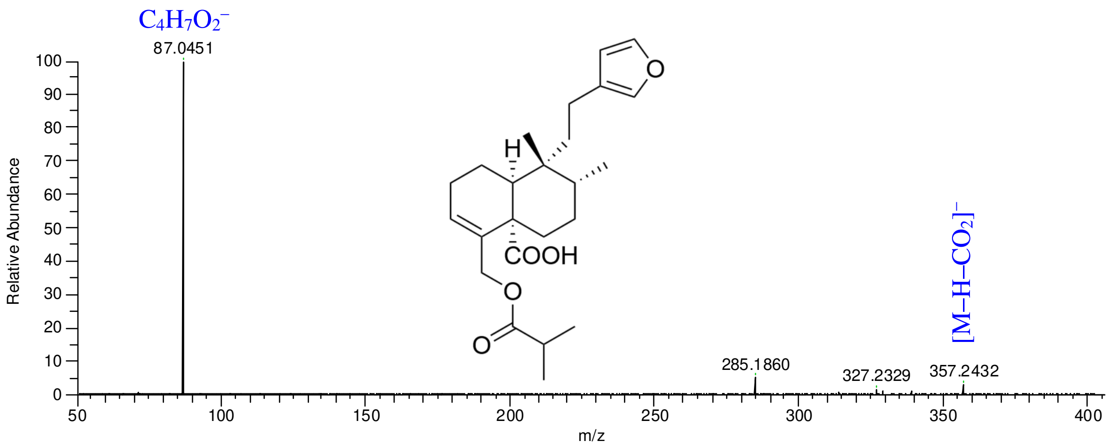

Figure S27. HR-ESI⁻-MS/MS spectrum of solidagoic acid K (**2**) with a normalized HCD collision energy of 30%. Precursor ion: *m*/*z* 401.2329 $[M-H]^-$, $C_{24}H_{33}O_5^-$.

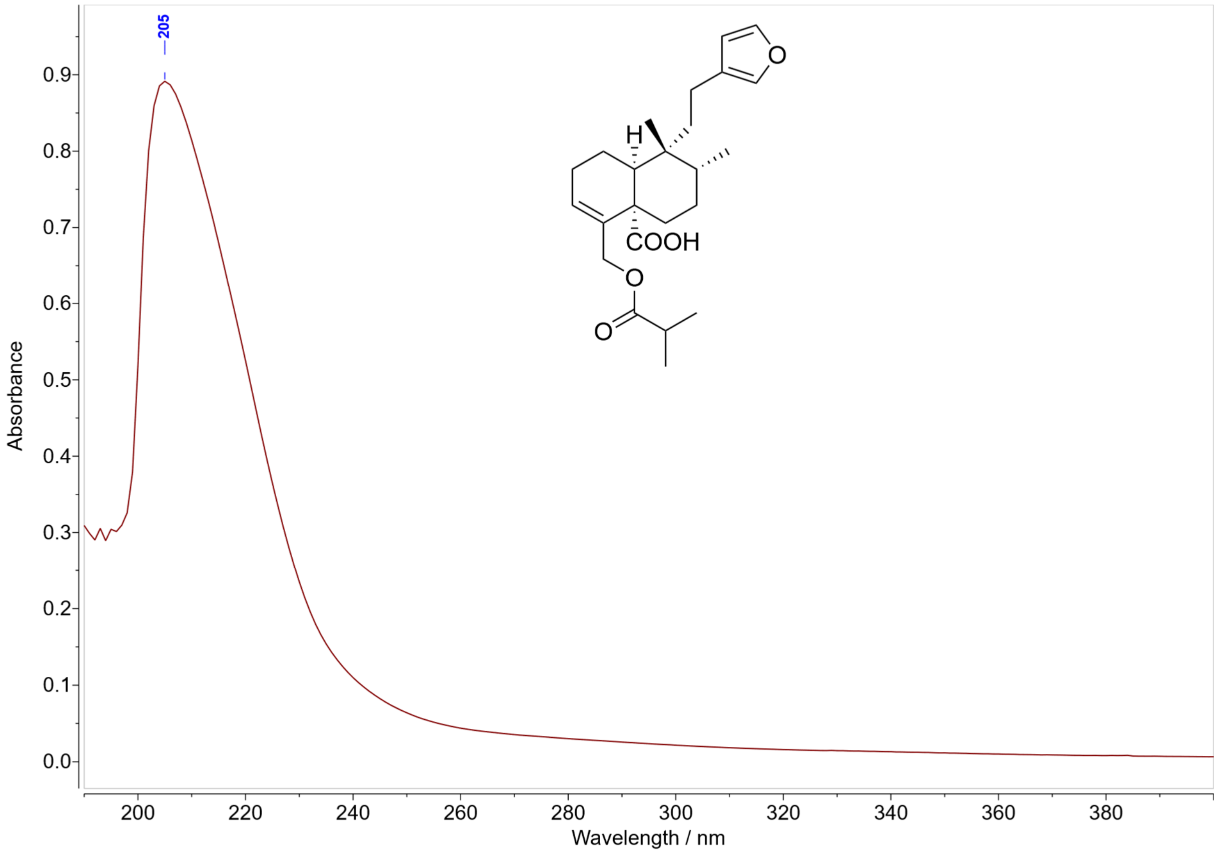

Figure S28. UV spectrum of solidagoic acid K (**2**) in ethanol.

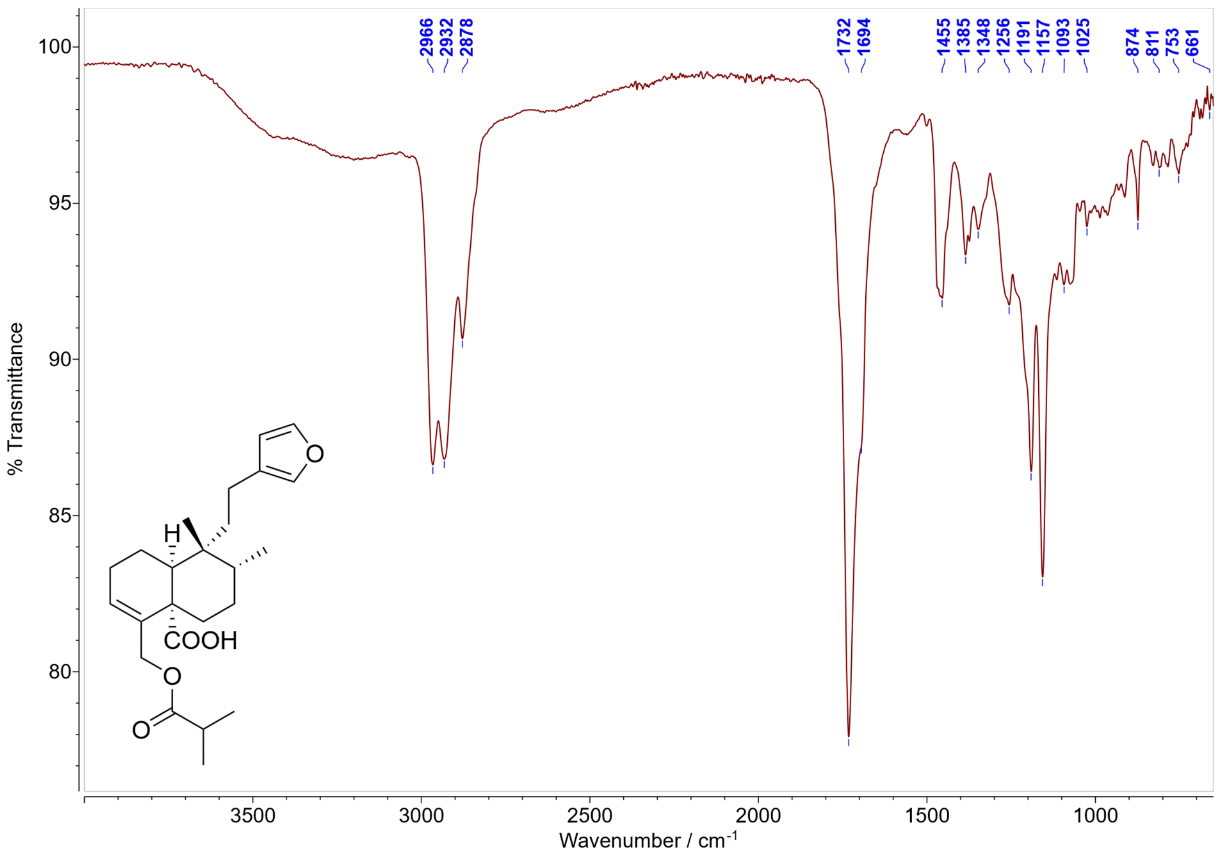

Figure S29. ATR-FTIR spectrum of solidagoic acid K (**2**).

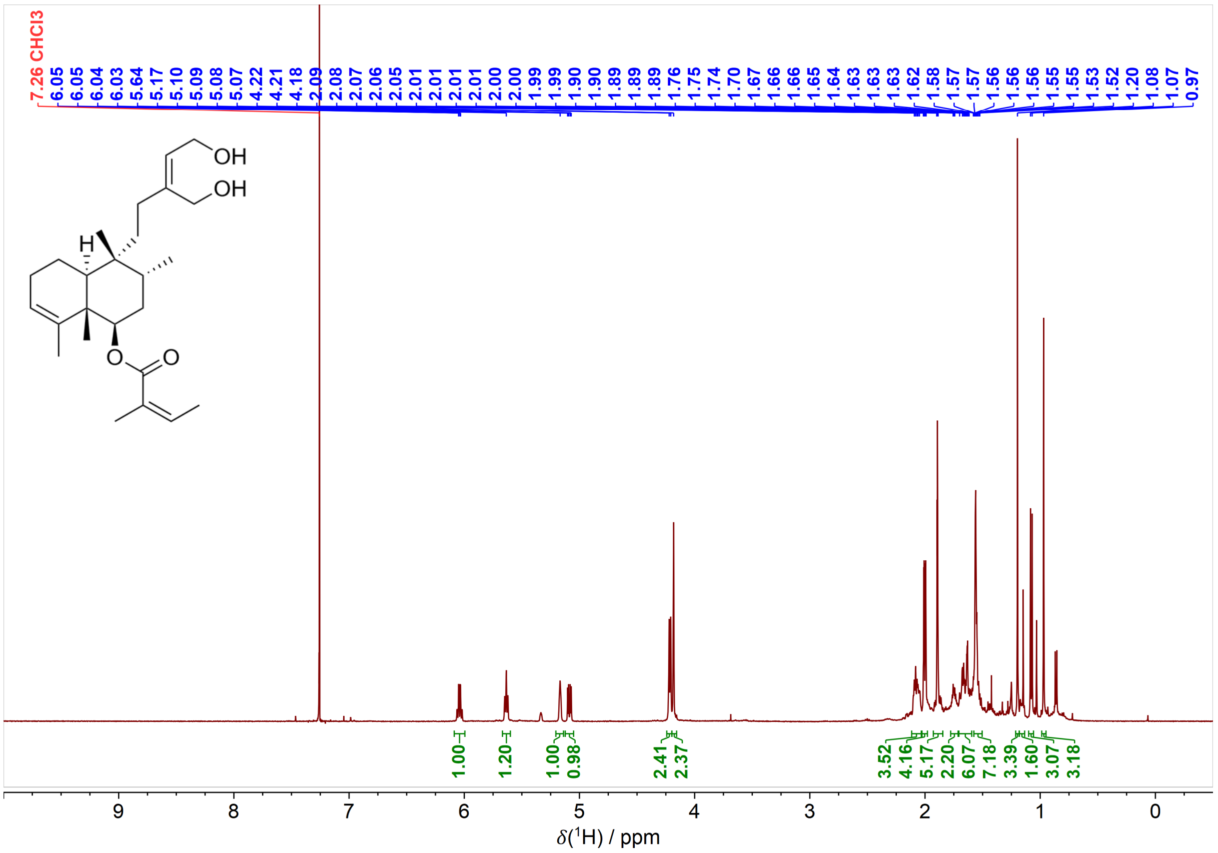

Figure S30. ^{1}H NMR spectrum of solidagodiol (**3**) (500 MHz, $CDCl_3$).

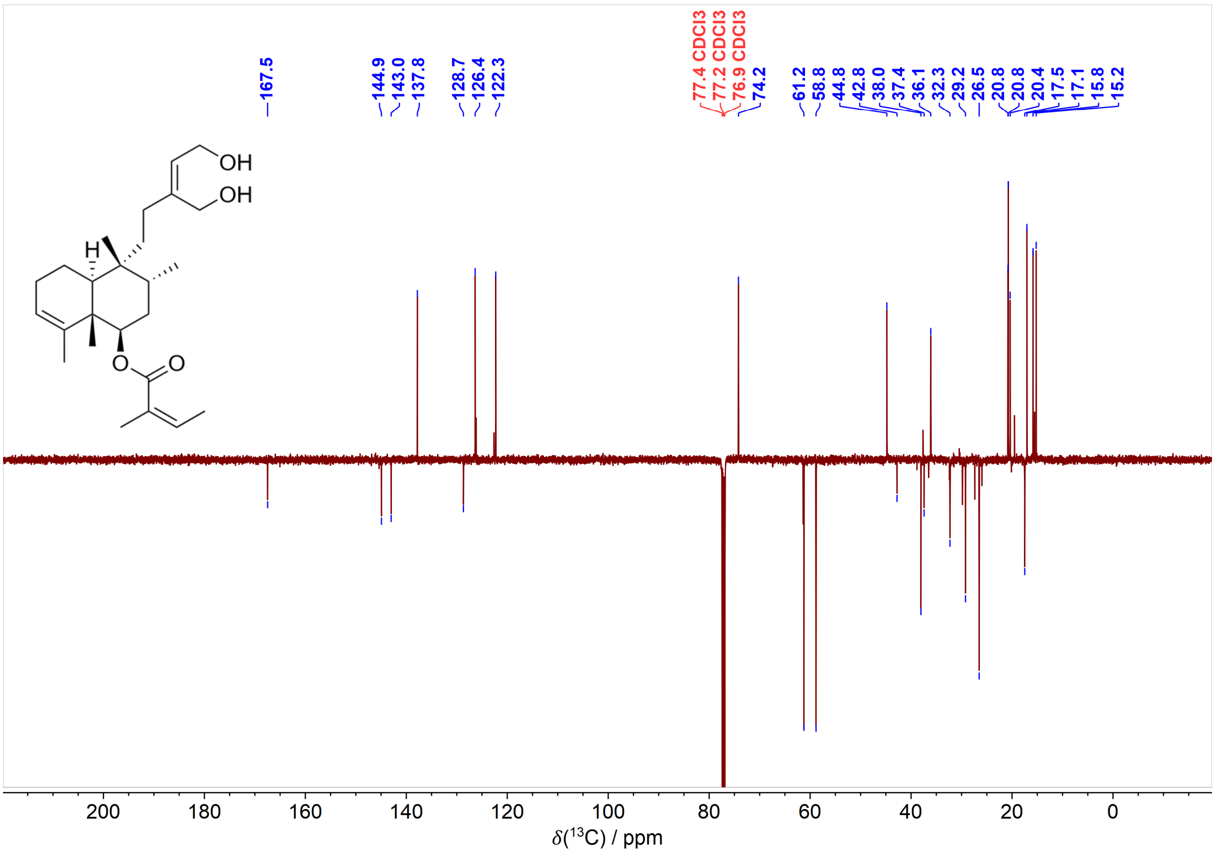

Figure S31. ^{13}C DEPTQ NMR spectrum of solidagodiol (**3**) (126 MHz, $CDCl_3$).

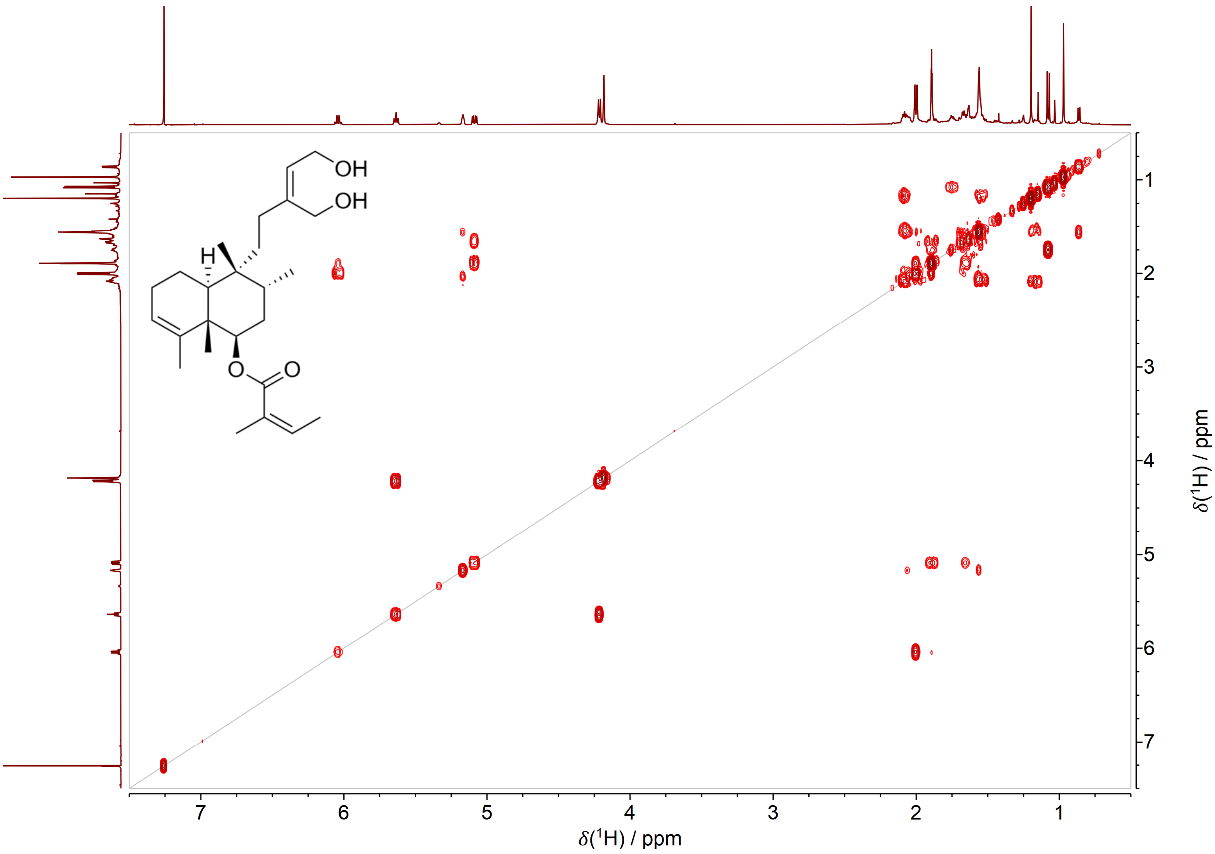

Figure S32. ^{1}H–^{1}H COSY NMR spectrum of solidagodiol (**3**) (500 MHz, $CDCl_3$).

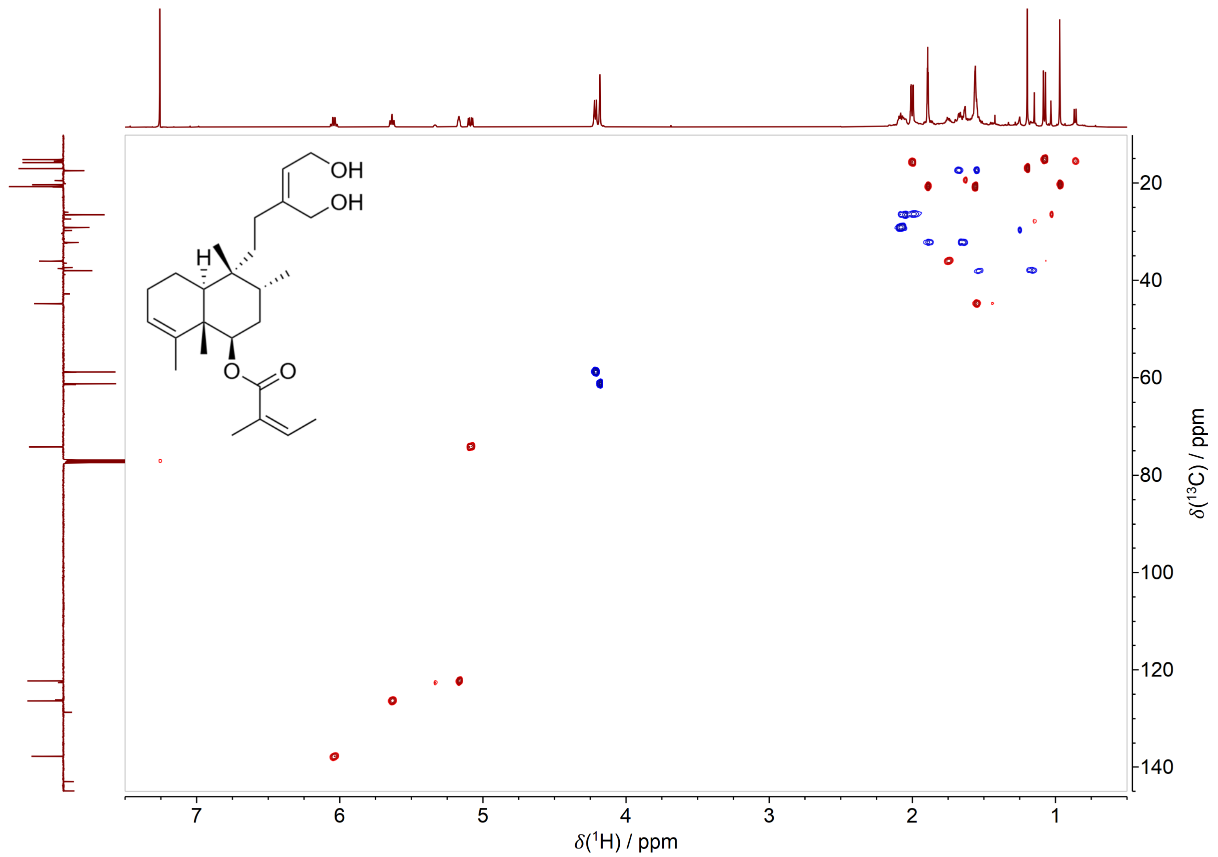

Figure S33. ^{1}H–^{13}C edHSQC NMR spectrum of solidagodiol (**3**) (500/126 MHz, $CDCl_3$).

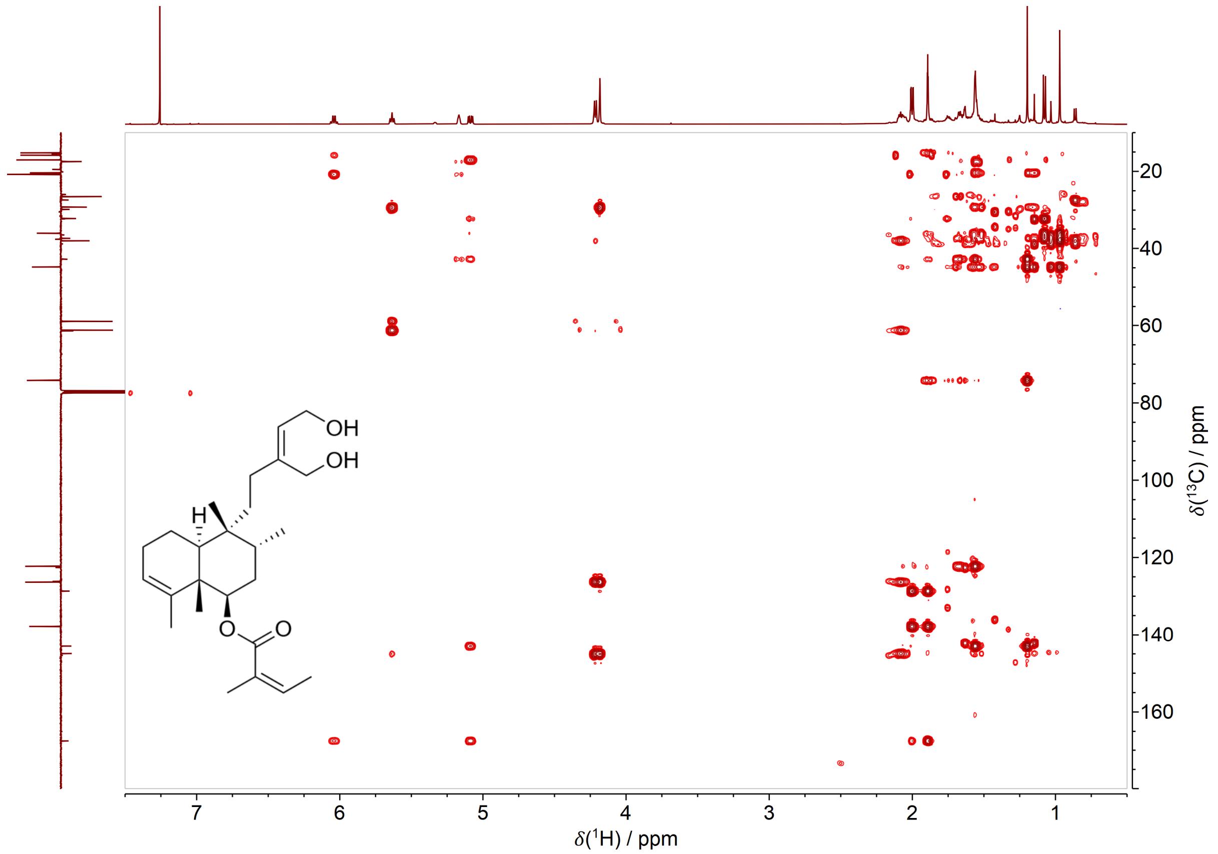

Figure S34. ^{1}H–^{13}C HMBC NMR spectrum of solidagodiol (**3**) (500/126 MHz, $CDCl_3$).

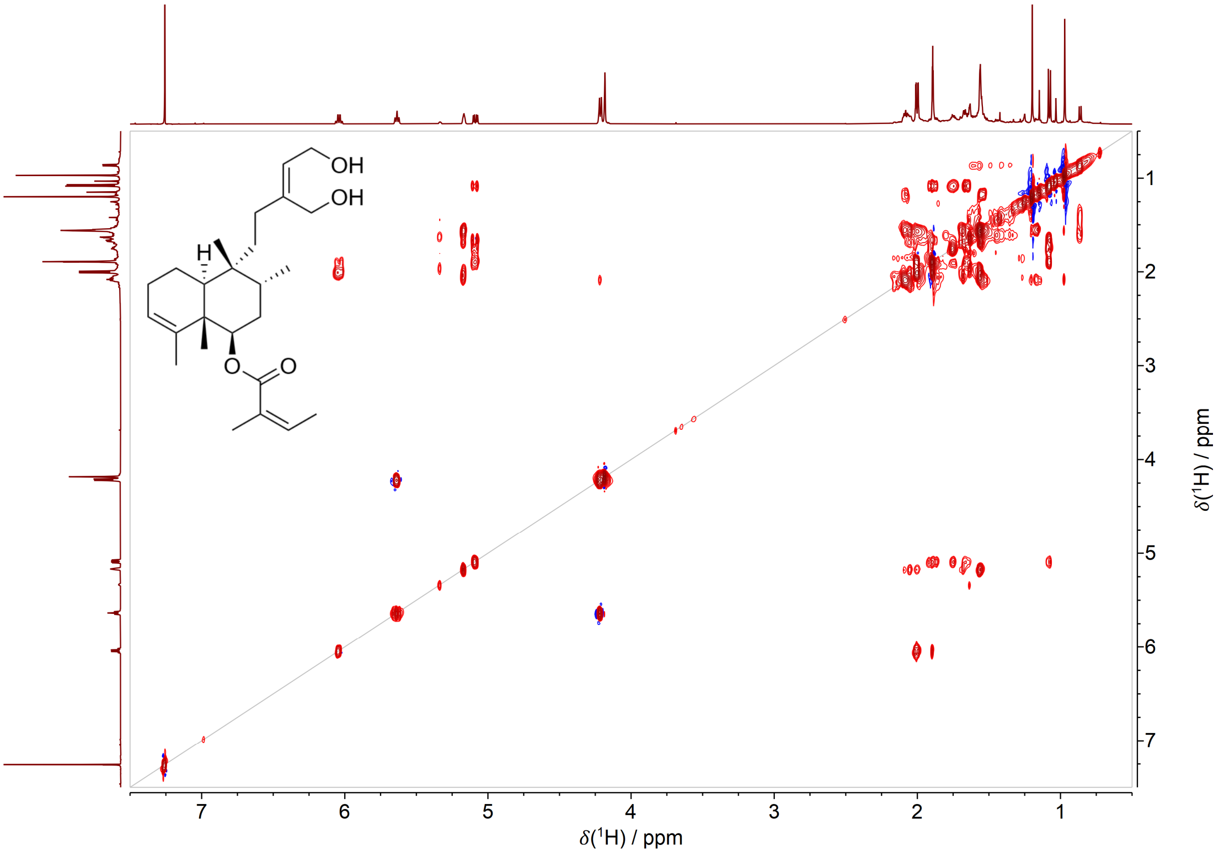

Figure S35. ^{1}H–^{1}H TOCSY NMR spectrum of solidagodiol (**3**) (500 MHz, $CDCl_3$).

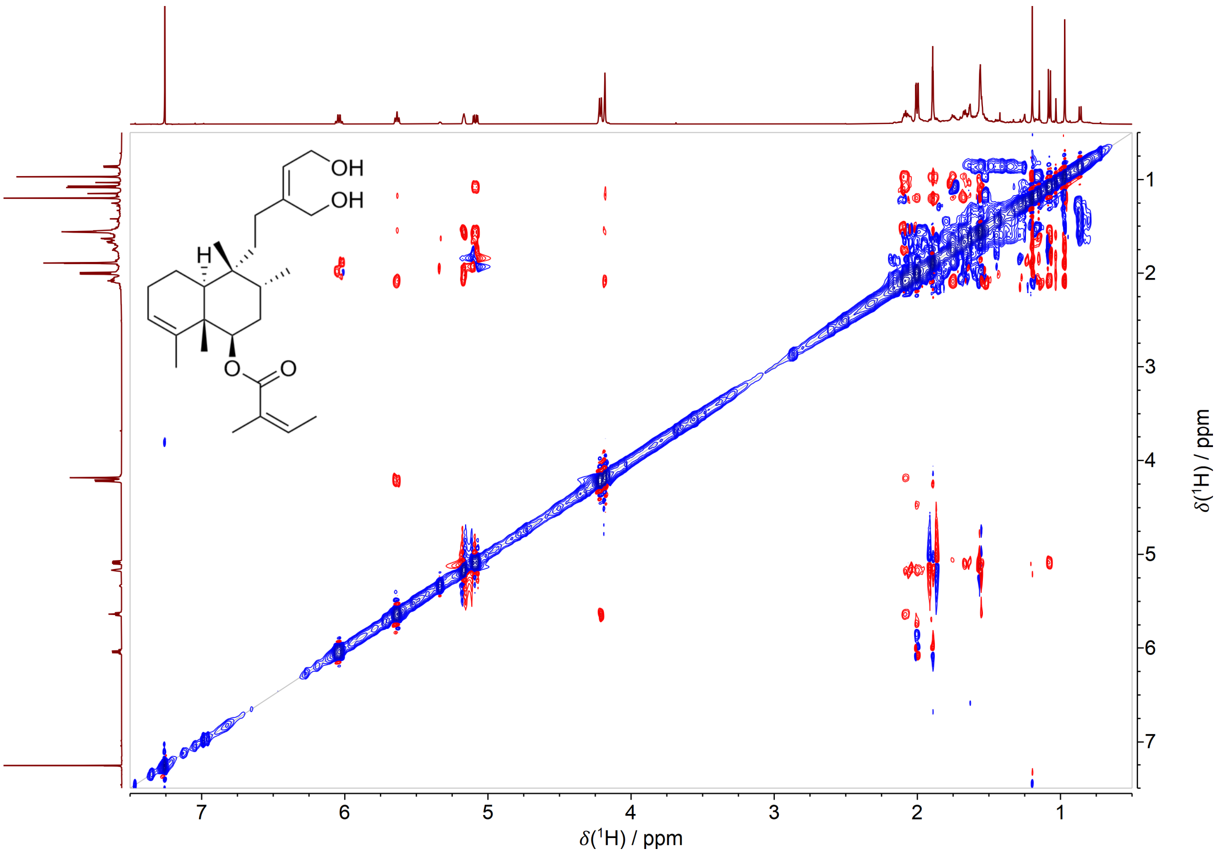

Figure S36. ^{1}H–^{1}H ROESY NMR spectrum of solidagodiol (**3**) (500 MHz, $CDCl_3$).

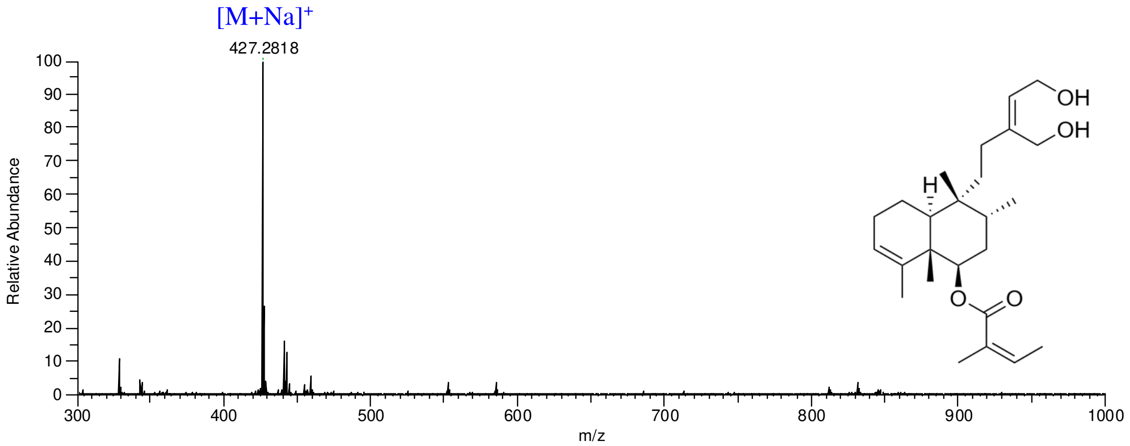

Figure S37. HR-ESI$^+$-MS spectrum of solidagodiol (**3**), *m*/*z* 427.2818 $[M+Na]^+$ (calculated for $C_{25}H_{40}O_4Na^+$, *m*/*z* 427.2819 $[M+Na]^+$, error: –0.3 ppm).

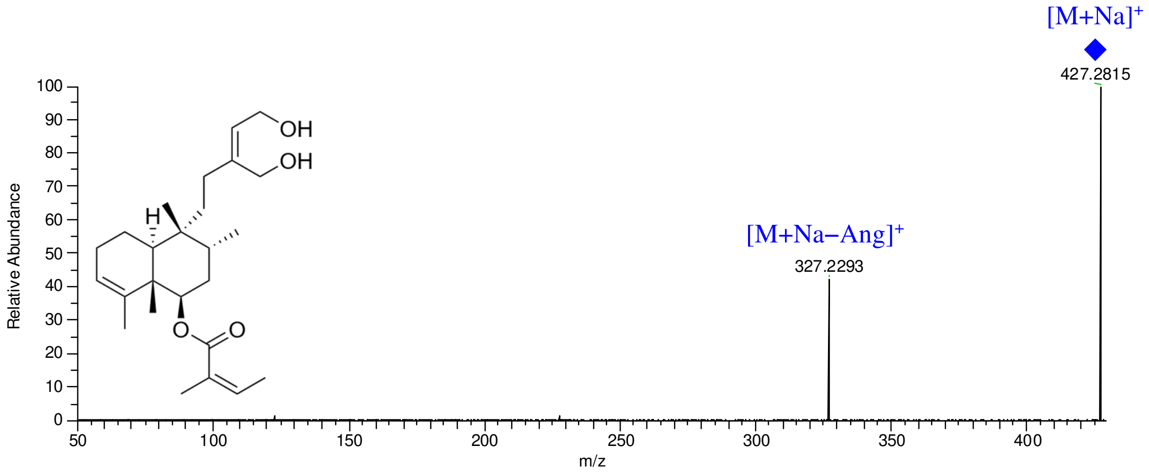

Figure S38. HR-ESI$^+$-MS/MS spectrum of solidagodiol (**3**) with a normalized HCD collision energy of 25%. Precursor ion: *m*/*z* 427.2815 $[M+Na]^+$, $C_{25}H_{40}O_4Na^+$. Ang denotes an angeloyloxy group ($C_5H_8O_2$).

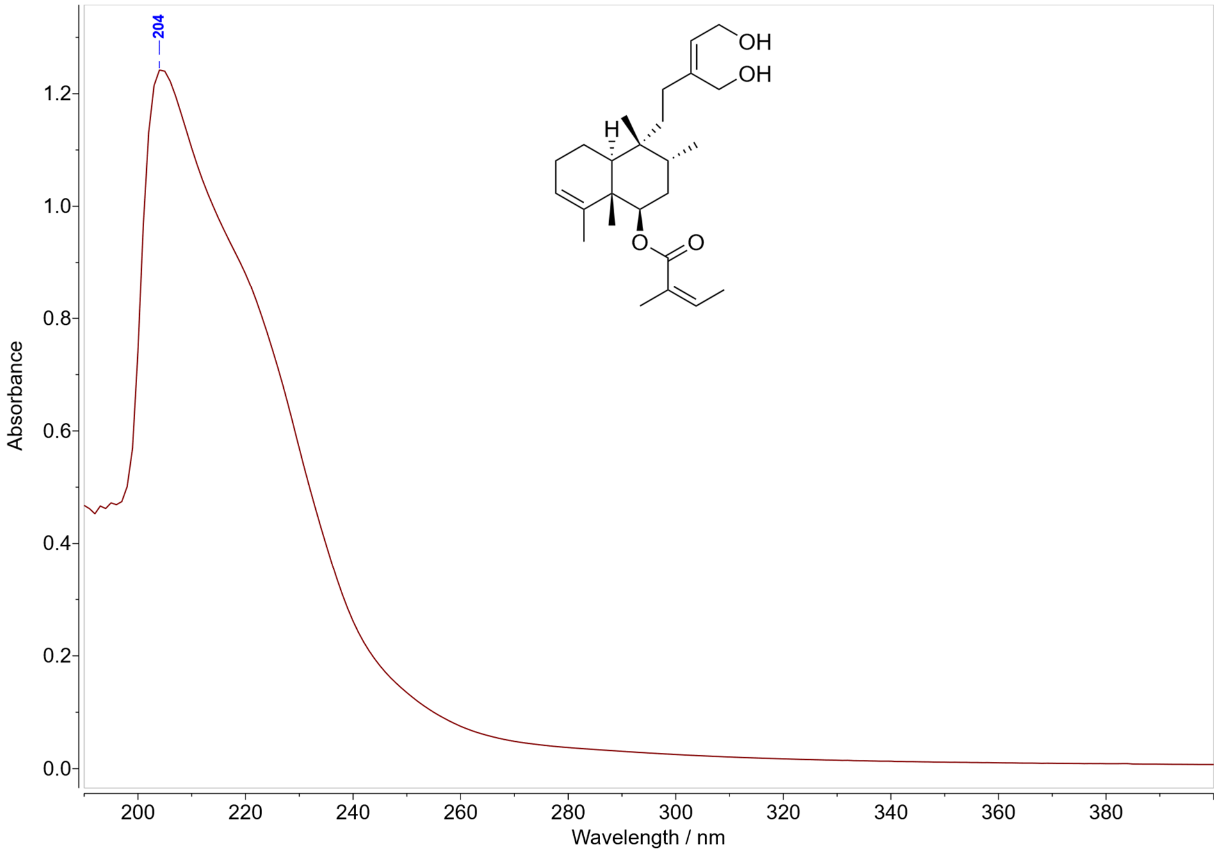

Figure S39. UV spectrum of solidagodiol (**3**) in ethanol.

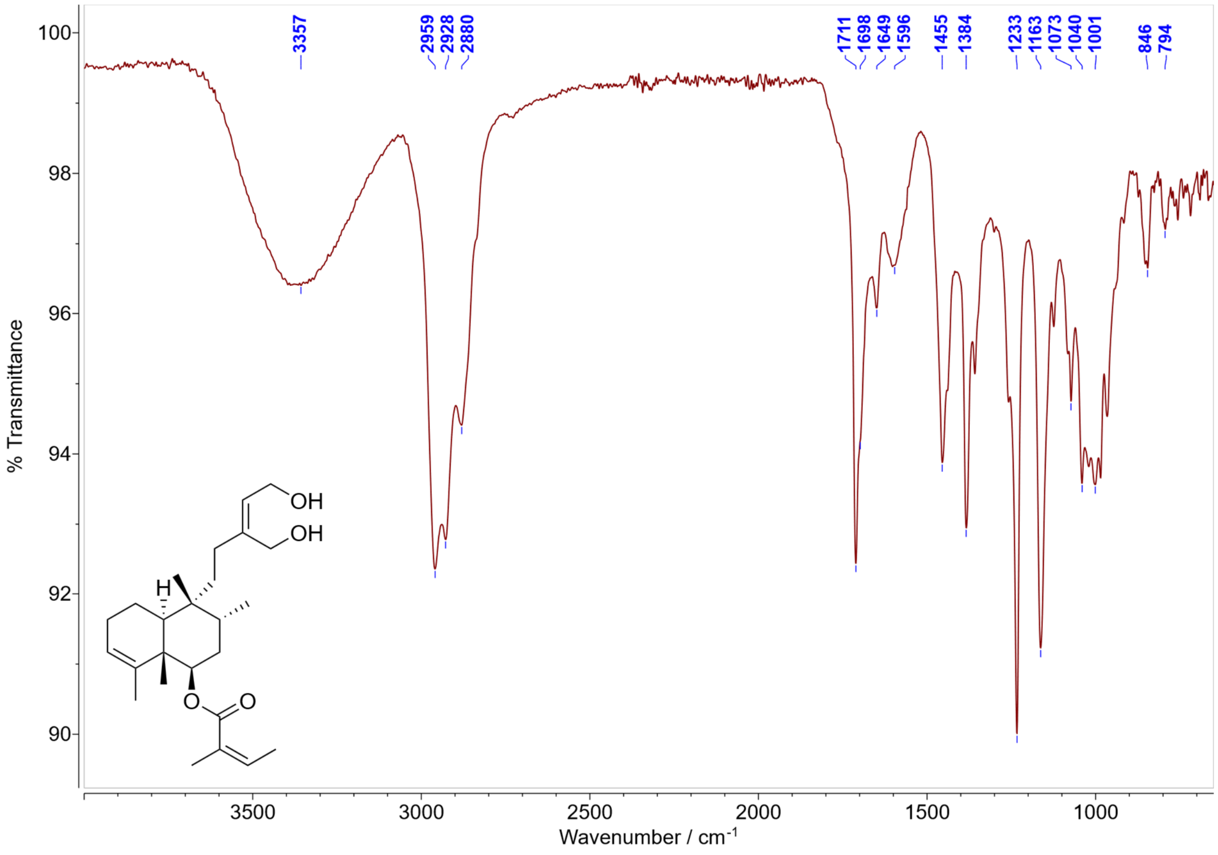

Figure S40. ATR-FTIR spectrum of solidagodiol (**3**).

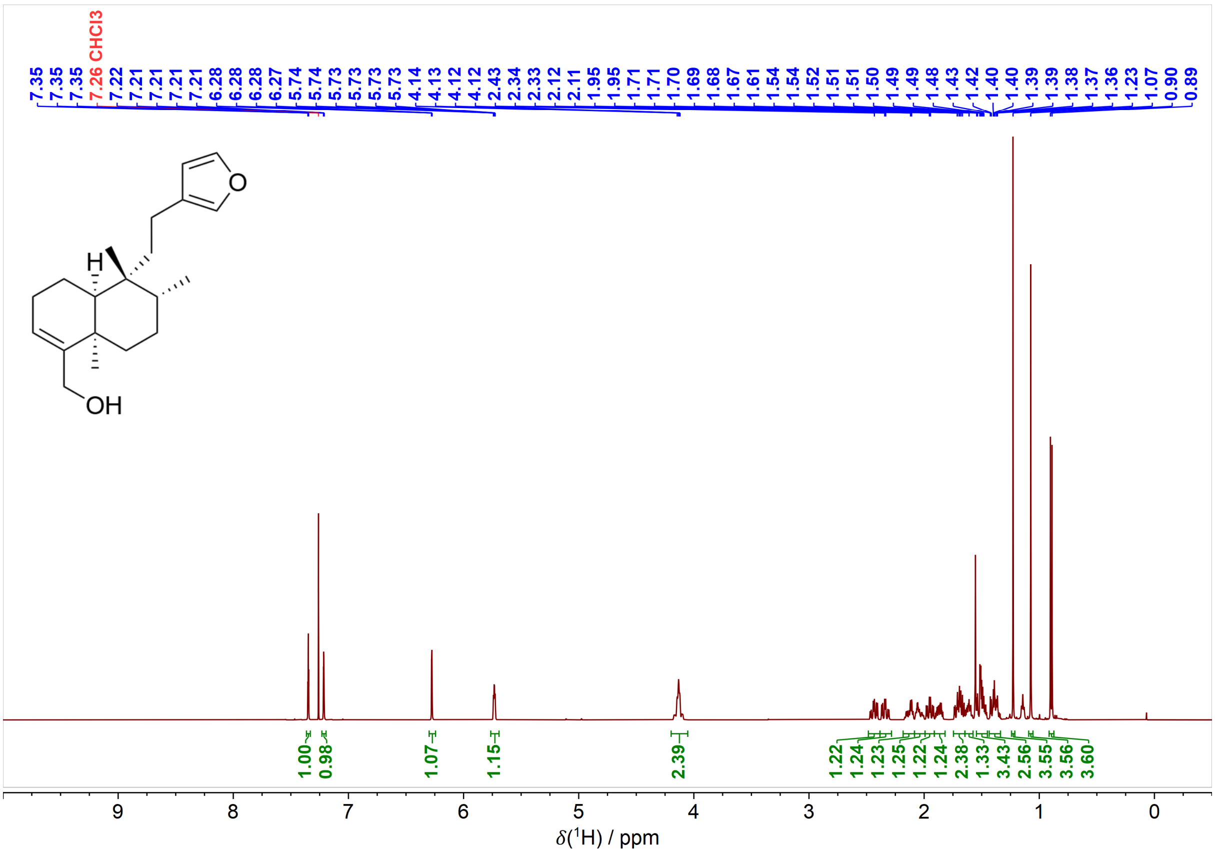

Figure S41. ^{1}H NMR spectrum of (−)-(5*R*,8*R*,9*R*,10*S*)-15,16-epoxy-*ent*-*neo*-cleroda-3,13,14-trien-18-ol (**4**) (500 MHz, $CDCl_3$).

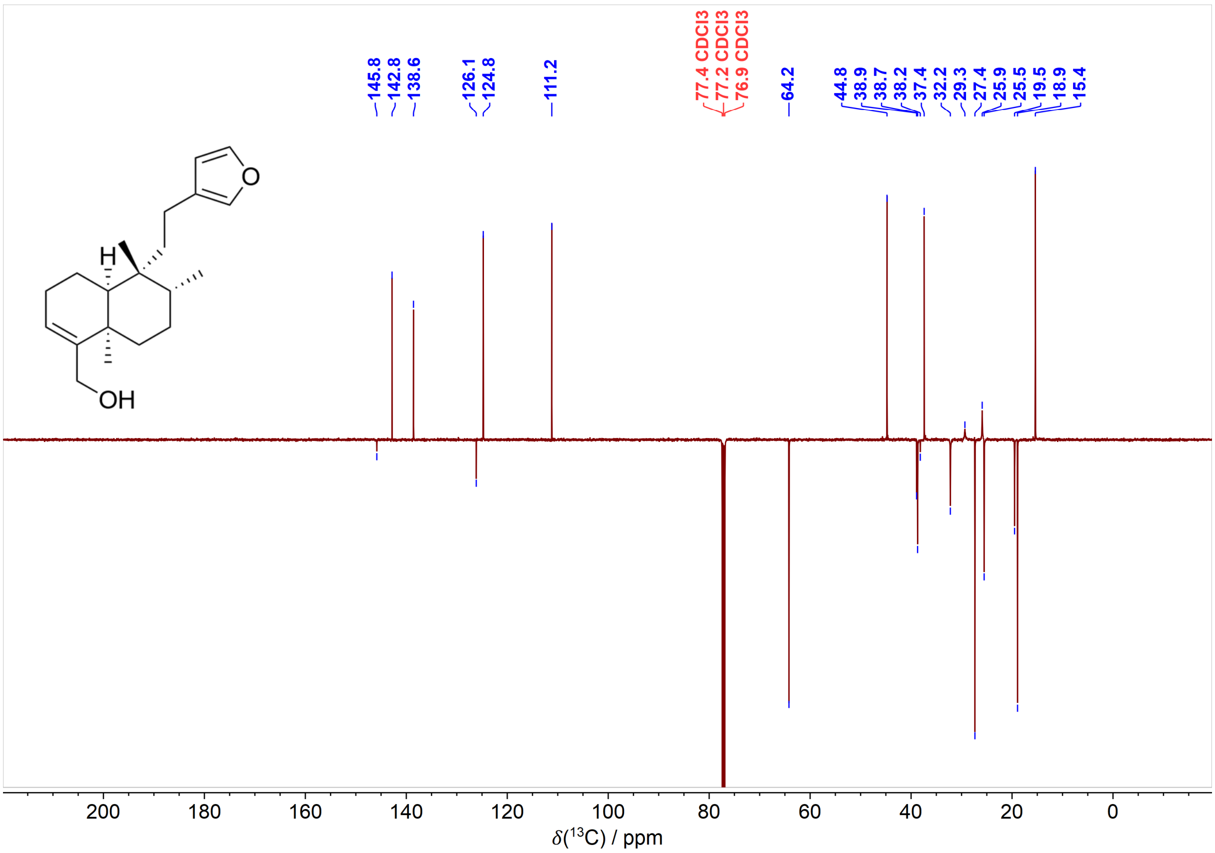

Figure S42. ^{13}C DEPTQ NMR spectrum of (−)-(5*R*,8*R*,9*R*,10*S*)-15,16-epoxy-*ent*-*neo*-cleroda-3,13,14-trien-18-ol (**4**) (126 MHz, $CDCl_3$).

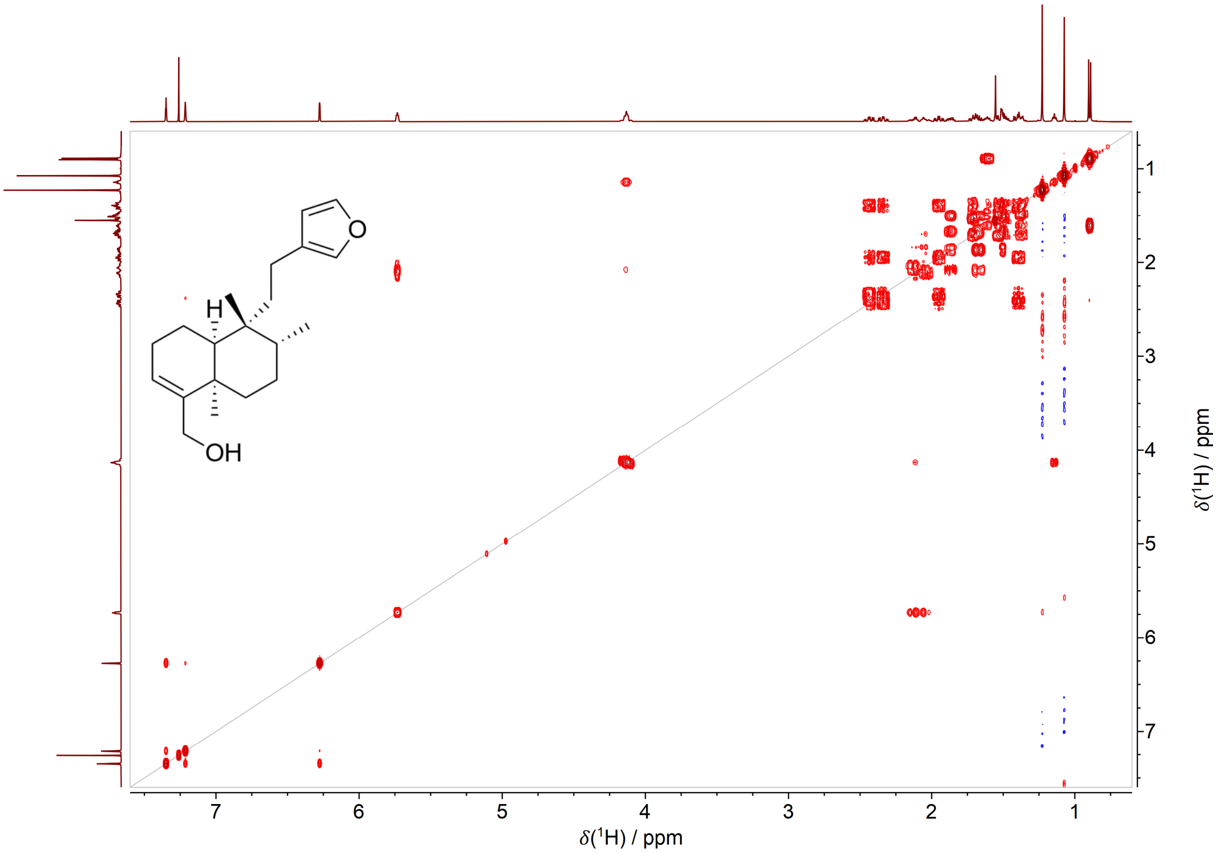

Figure S43. ^{1}H–^{1}H COSY NMR spectrum of (−)-(5*R*,8*R*,9*R*,10*S*)-15,16-epoxy-*ent*-*neo*-cleroda-3,13,14-trien-18-ol (**4**) (500 MHz, $CDCl_3$).

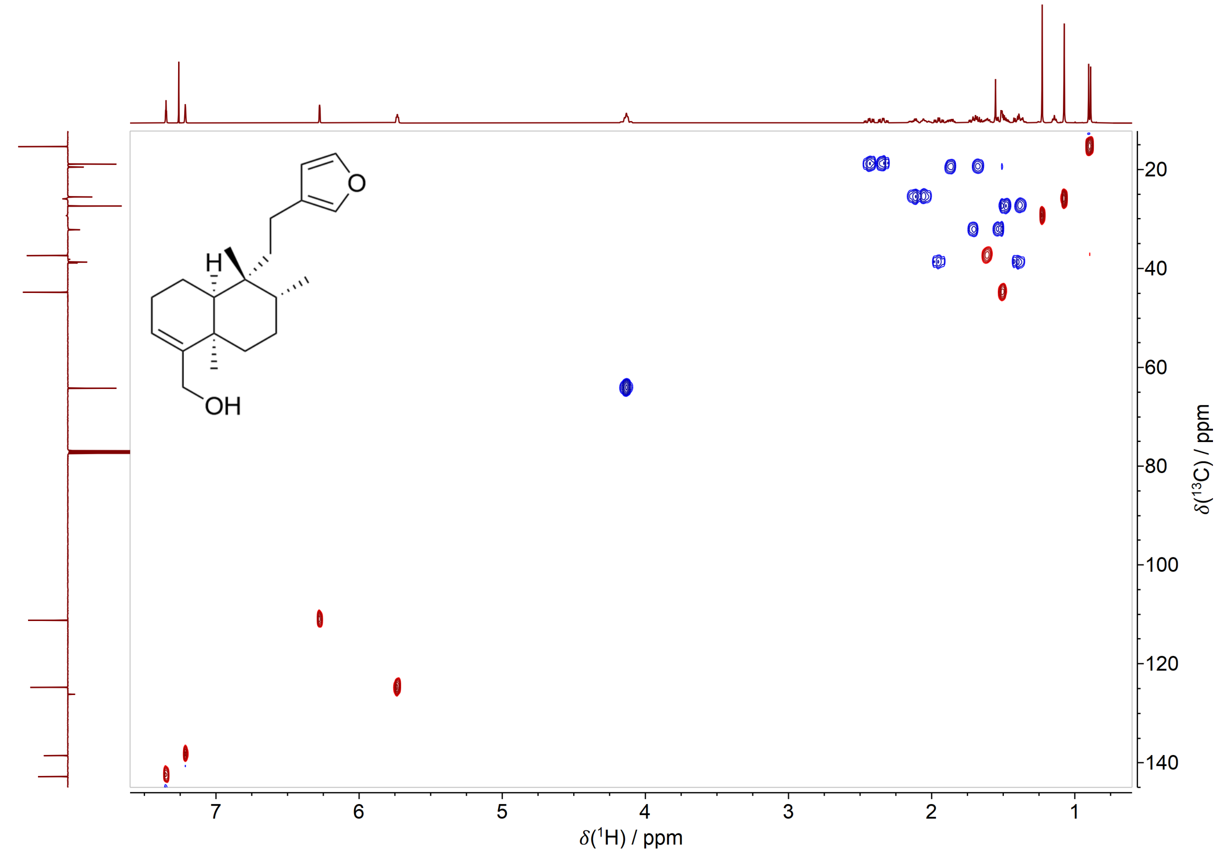

Figure S44. ^{1}H–^{13}C edHSQC NMR spectrum of (−)-(5*R*,8*R*,9*R*,10*S*)-15,16-epoxy-*ent*-*neo*-cleroda-3,13,14-trien-18-ol (**4**) (500/126 MHz, $CDCl_3$).

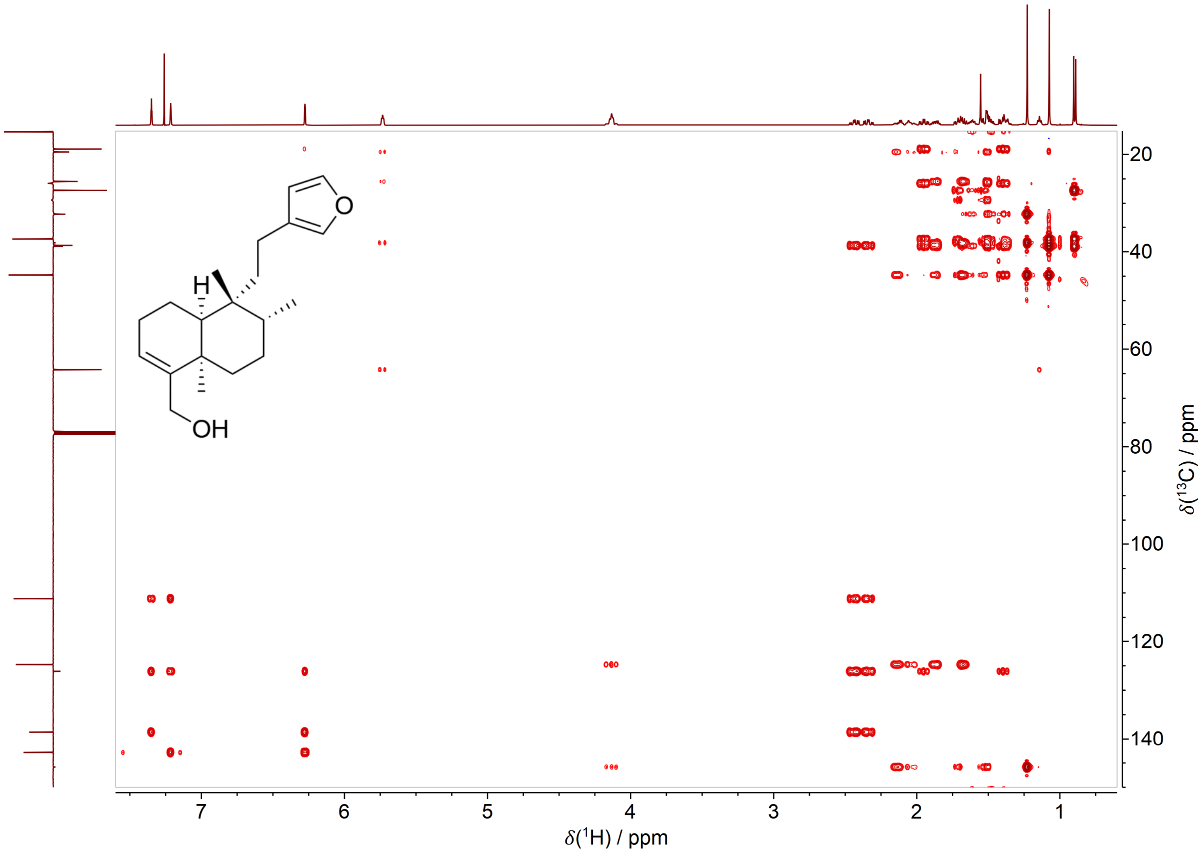

Figure S45. ^{1}H–^{13}C HMBC NMR spectrum of (−)-(5*R*,8*R*,9*R*,10*S*)-15,16-epoxy-*ent*-*neo*-cleroda-3,13,14-trien-18-ol (**4**) (500/126 MHz, $CDCl_3$).

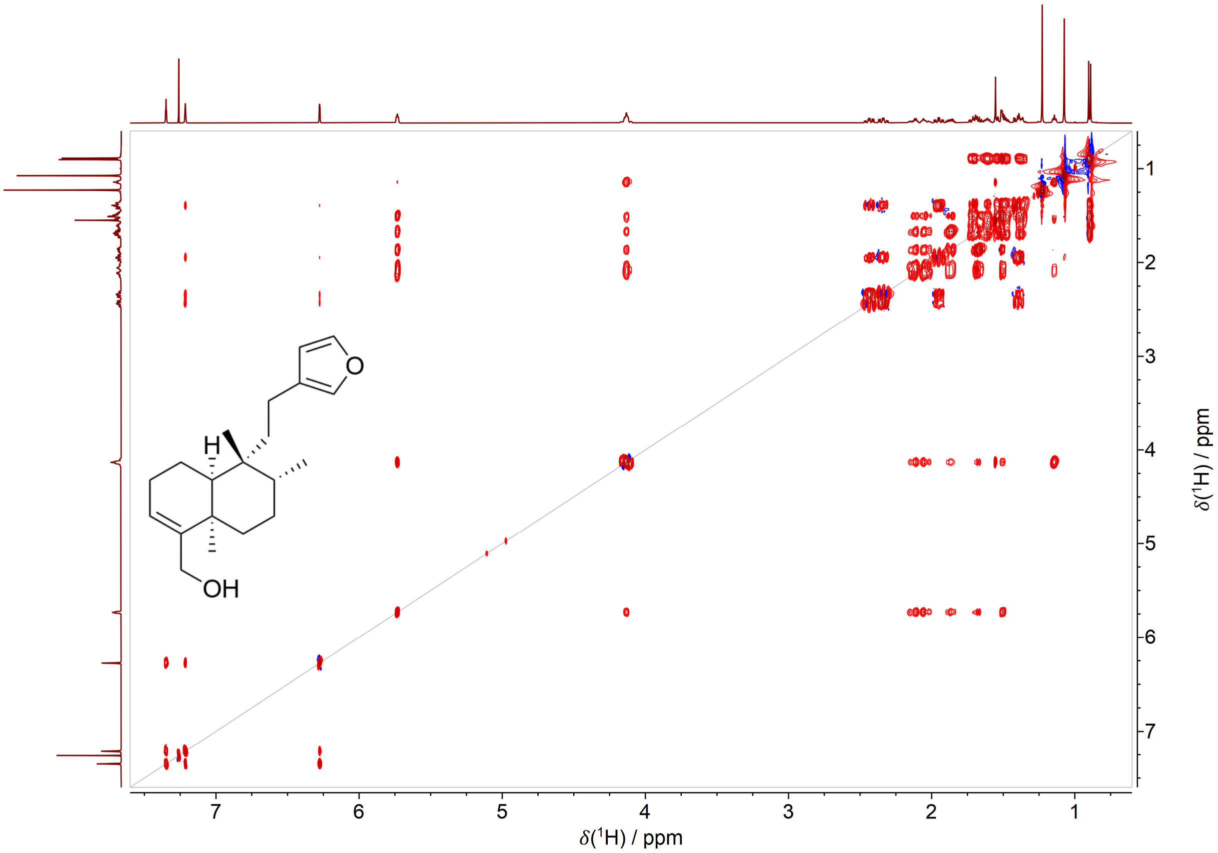

Figure S46. ^{1}H–^{1}H TOCSY NMR spectrum of (−)-(5*R*,8*R*,9*R*,10*S*)-15,16-epoxy-*ent*-*neo*-cleroda-3,13,14-trien-18-ol (**4**) (500 MHz, $CDCl_3$).

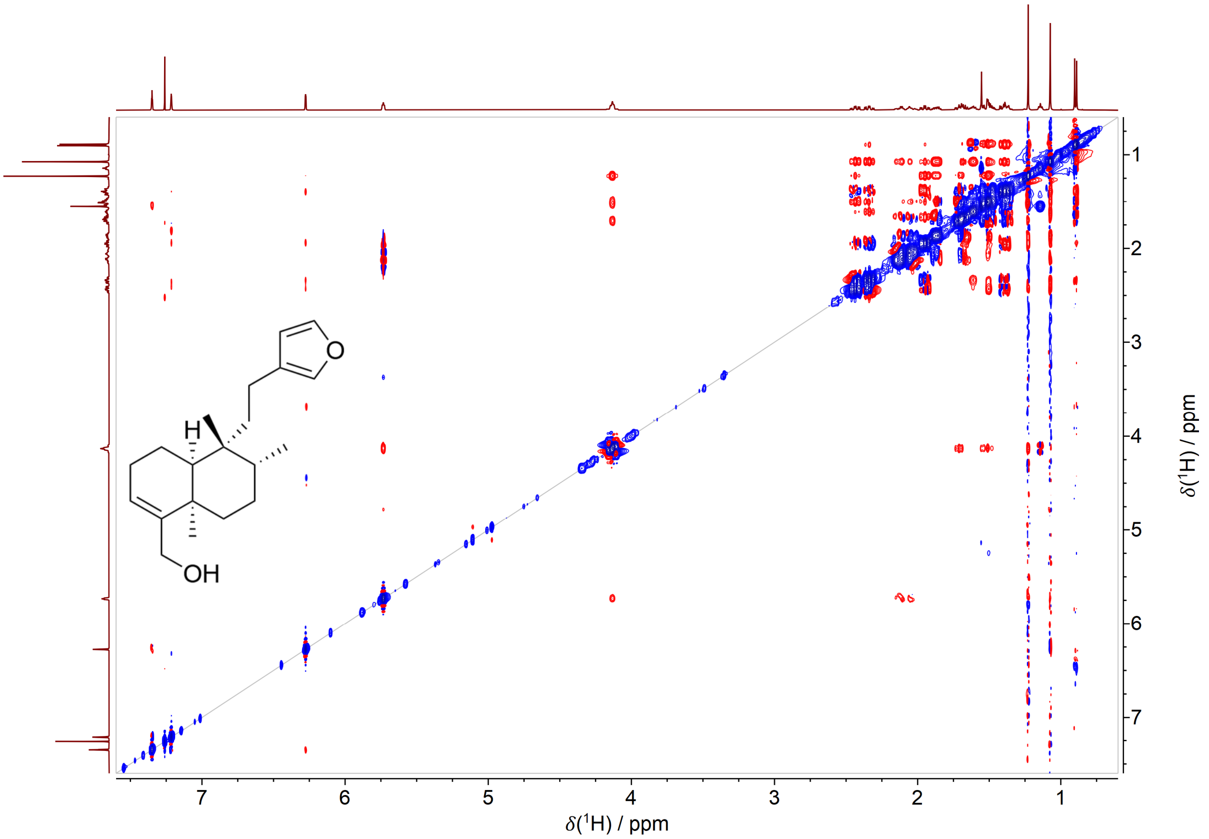

Figure S47. ^{1}H–^{1}H ROESY NMR spectrum of (−)-(5*R*,8*R*,9*R*,10*S*)-15,16-epoxy-*ent*-*neo*-cleroda-3,13,14-trien-18-ol (**4**) (500 MHz, $CDCl_3$).

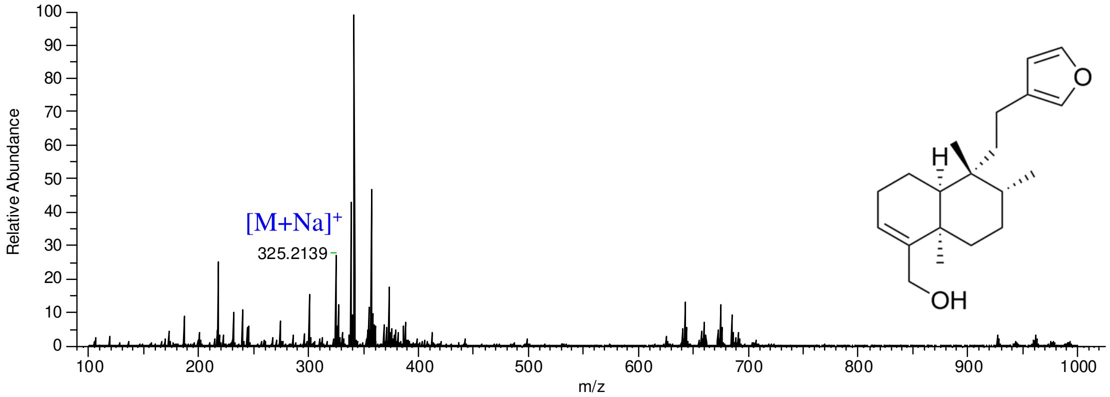

Figure S48. HR-ESI^{+}-MS spectrum of (−)-(5*R*,8*R*,9*R*,10*S*)-15,16-epoxy-*ent*-*neo*-cleroda-3,13,14-trien-18-ol (**4**), *m*/*z* 325.2139 $[M+Na]^+$ (calculated for $C_{20}H_{30}O_2Na^+$, *m*/*z* 325.2138 $[M+Na]^+$, error: 0.2 ppm).

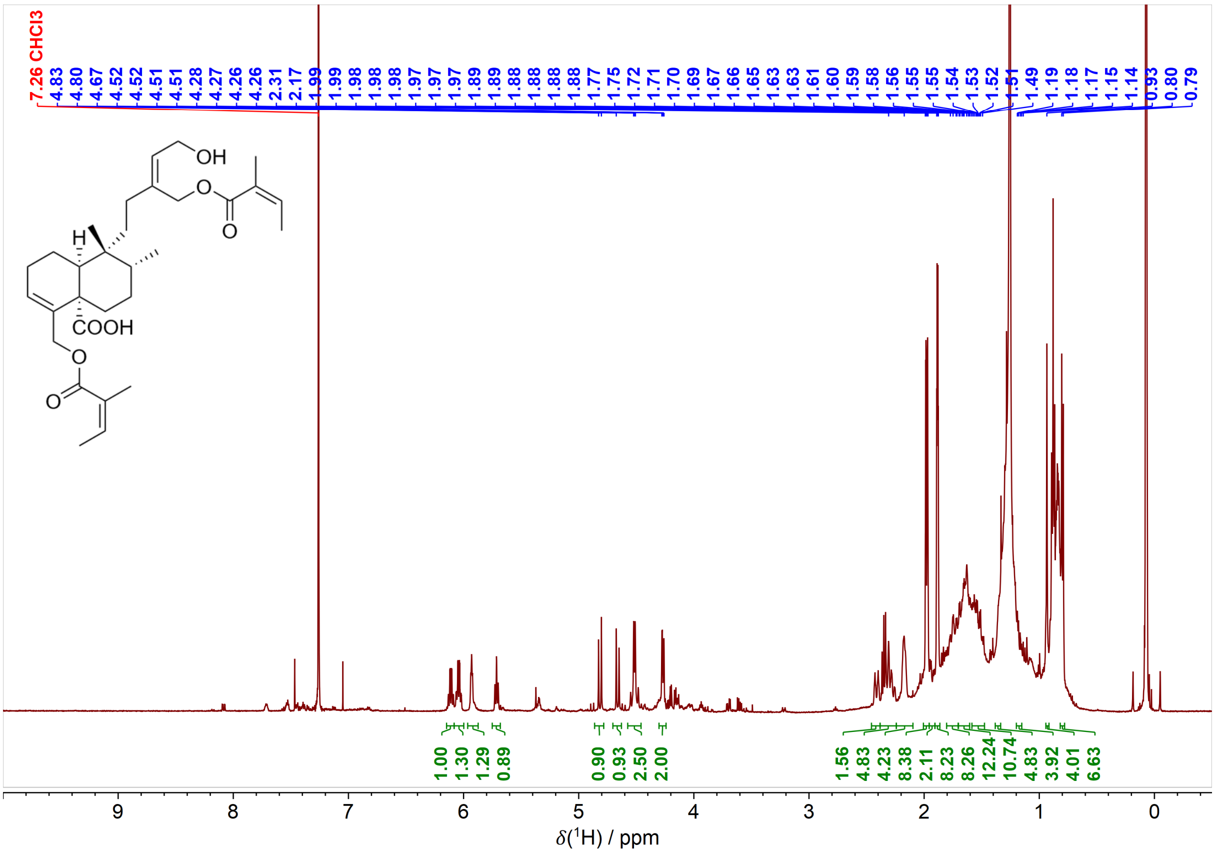

Figure S49. ^{1}H NMR spectrum of solidagoic acid J (**5**) (500 MHz, $CDCl_3$).

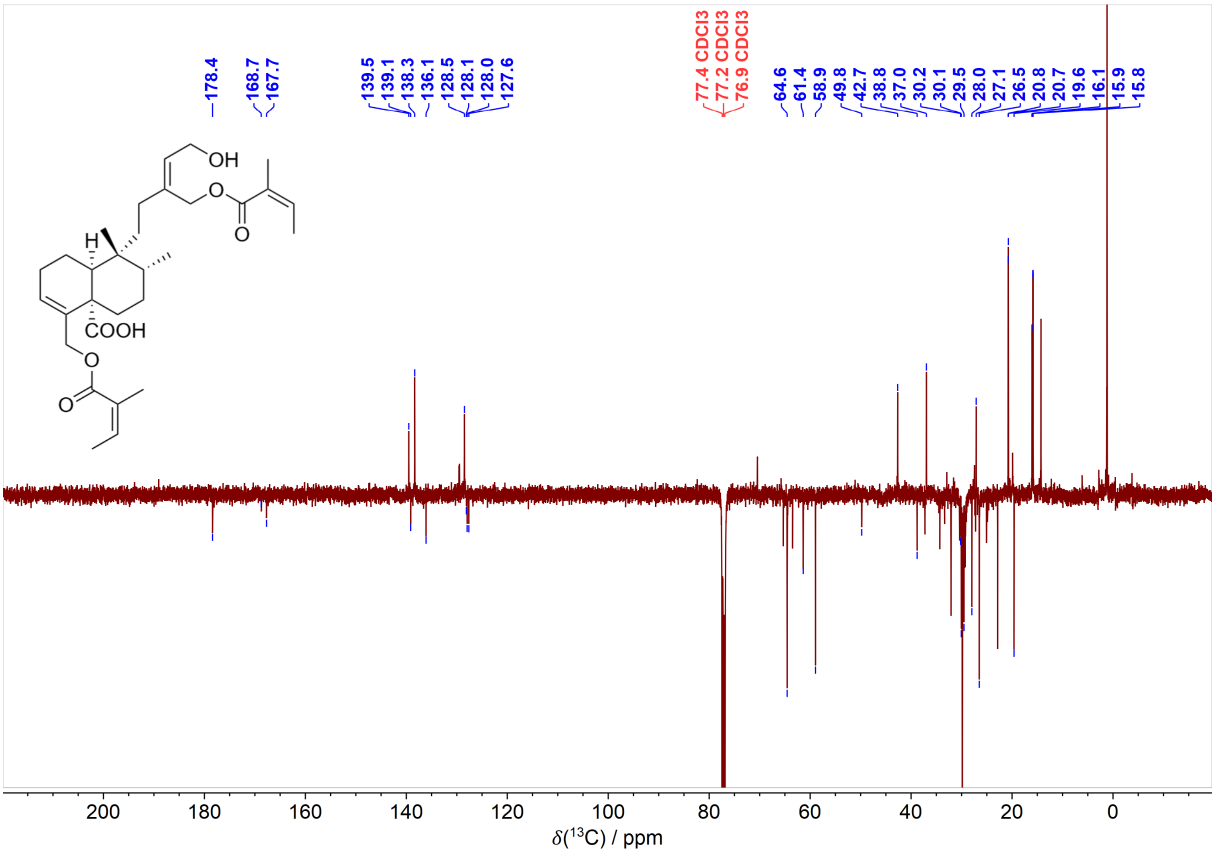

Figure S50. ^{13}C DEPTQ NMR spectrum of solidagoic acid J (**5**) (126 MHz, $CDCl_3$).

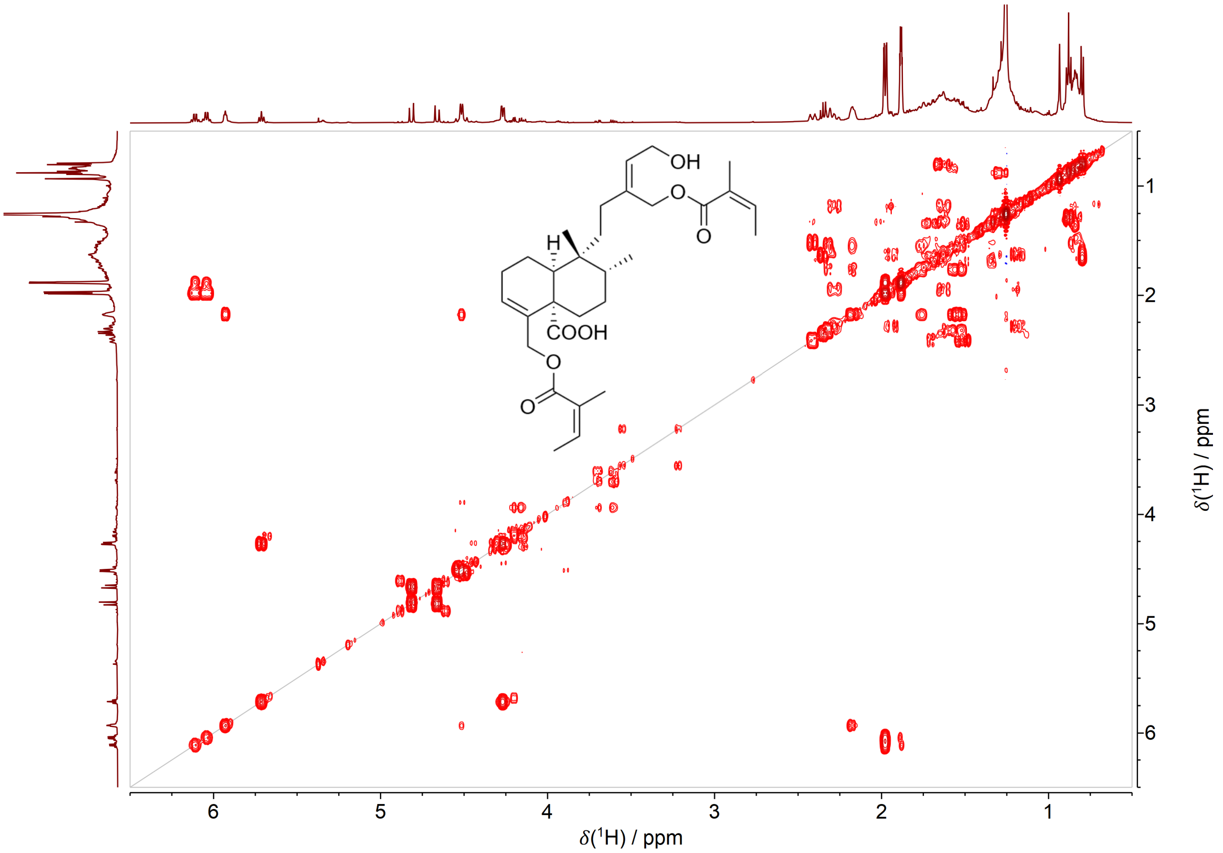

Figure S51. ^{1}H–^{1}H COSY NMR spectrum of solidagoic acid J (**5**) (500 MHz, $CDCl_3$).

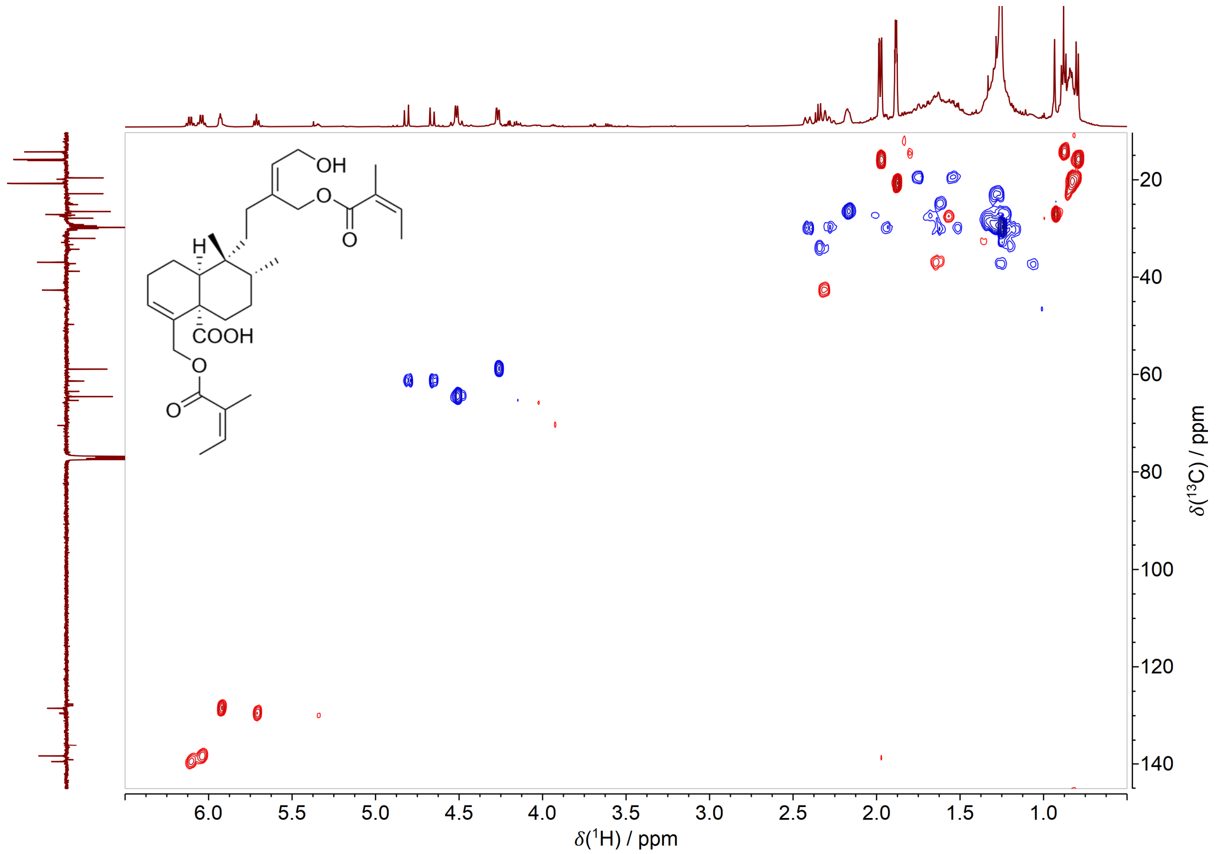

Figure S52. ^{1}H–^{13}C edHSQC NMR spectrum of solidagoic acid J (**5**) (500/126 MHz, $CDCl_3$).

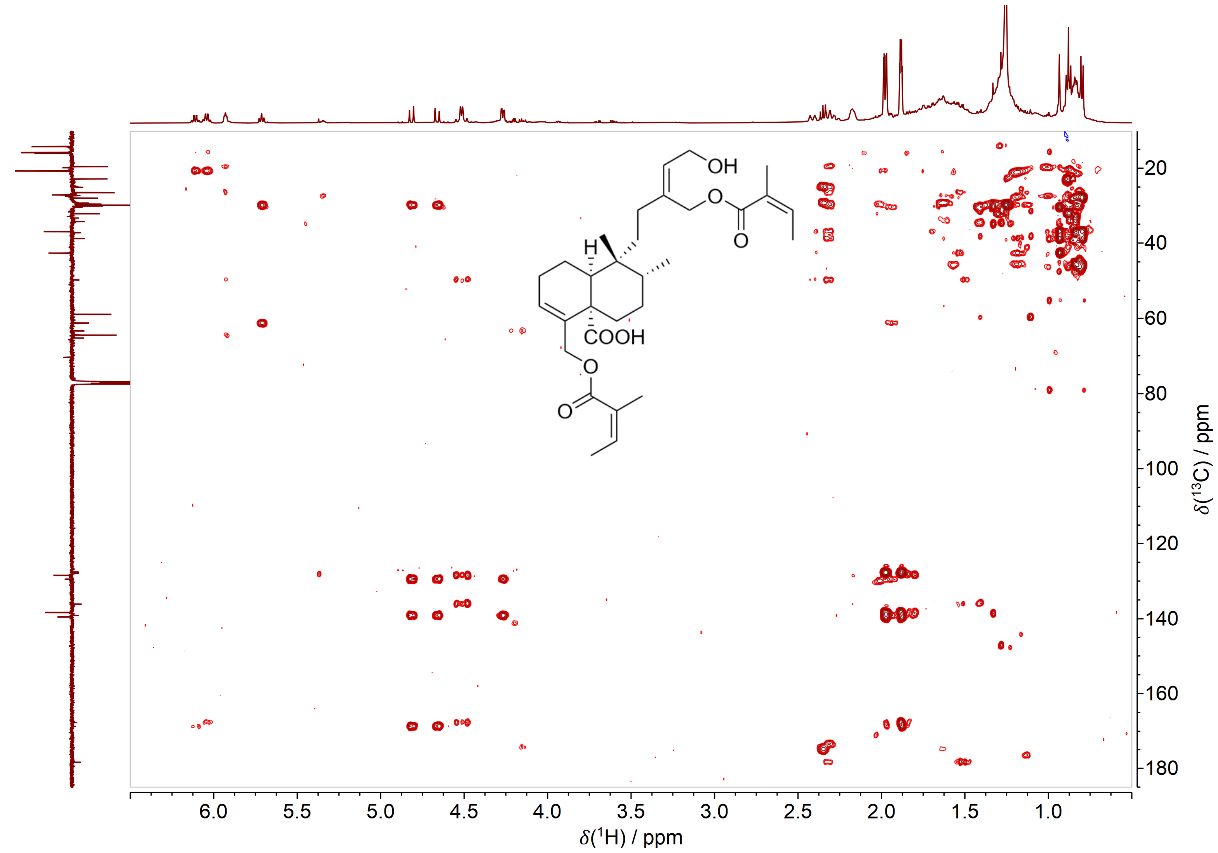

Figure S53. ¹H–¹³C HMBC NMR spectrum of solidagoic acid J (**5**) (500/126 MHz, CDCl₃).

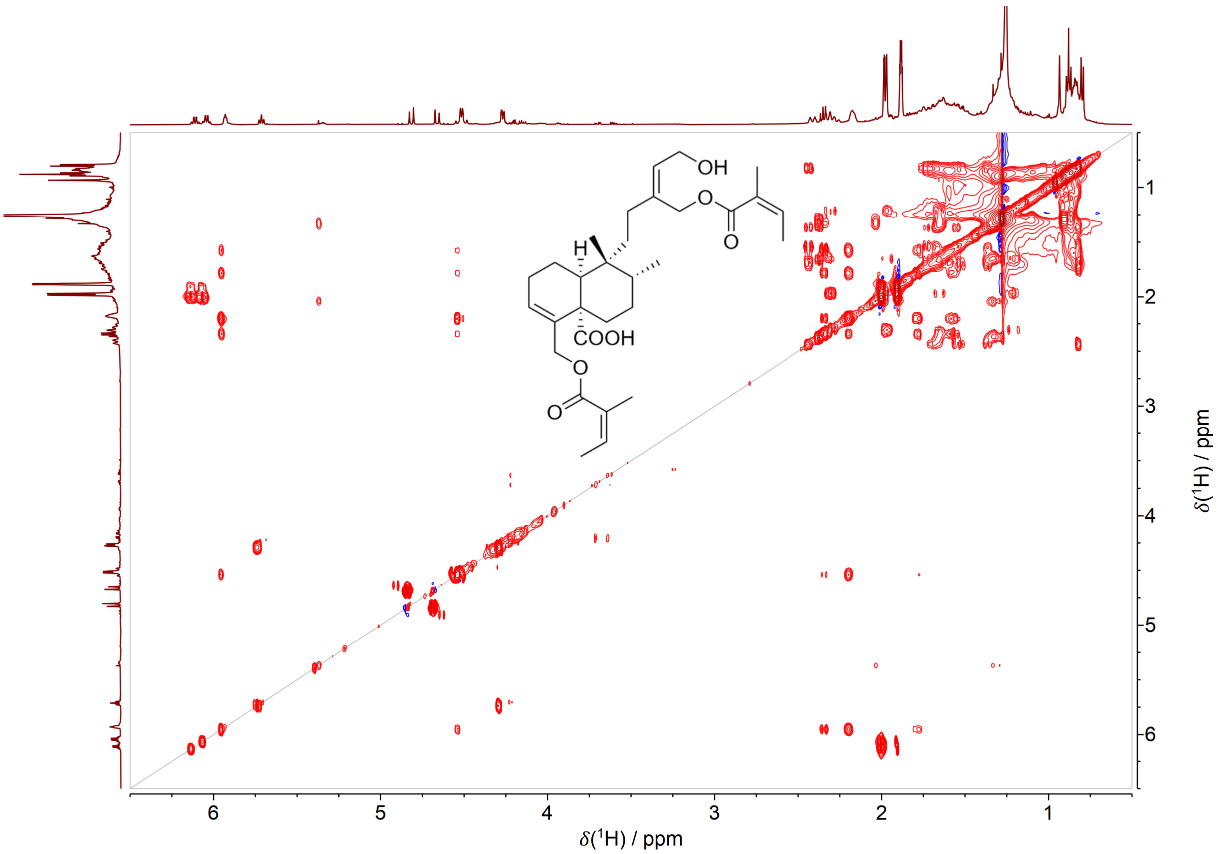

Figure S54. ¹H–¹H TOCSY NMR spectrum of solidagoic acid J (**5**) (500 MHz, CDCl₃).

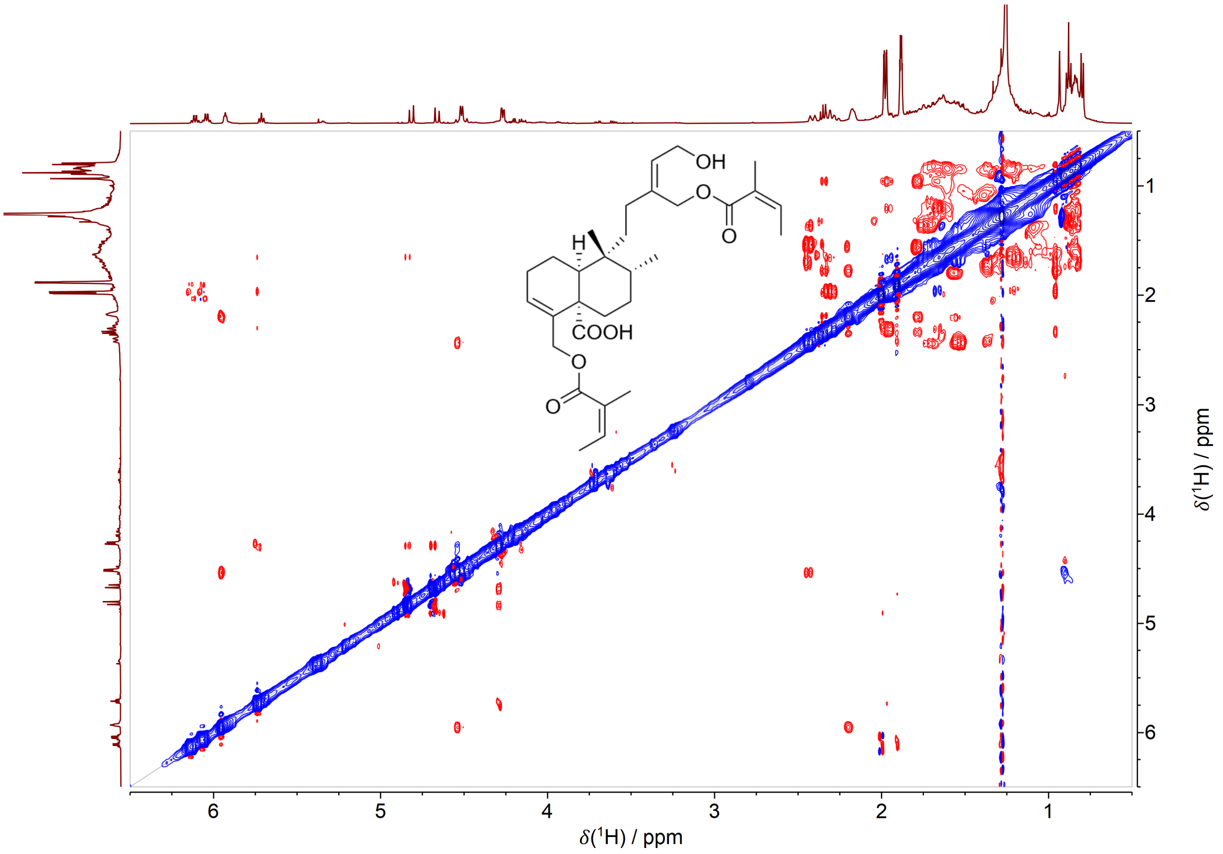

Figure S55. ^1H–^1H ROESY NMR spectrum of solidagoic acid J (**5**) (500 MHz, $CDCl_3$).

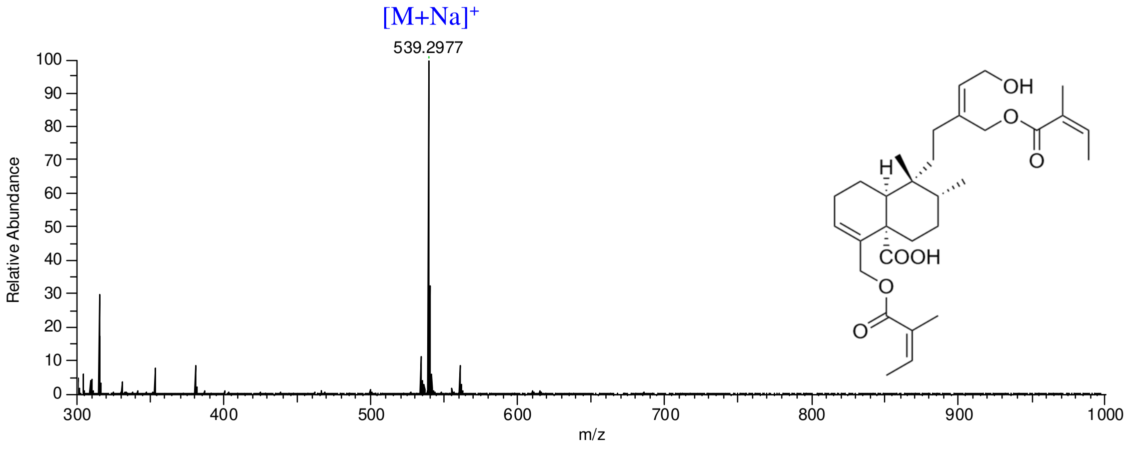

Figure S56. HR-ESI$^+$-MS spectrum of solidagoic acid J (**5**), *m/z* 539.2977 $[M+Na]^+$ (calculated for $C_{30}H_{44}O_7Na^+$, *m/z* 539.2979 $[M+Na]^+$, error: –0.5 ppm).

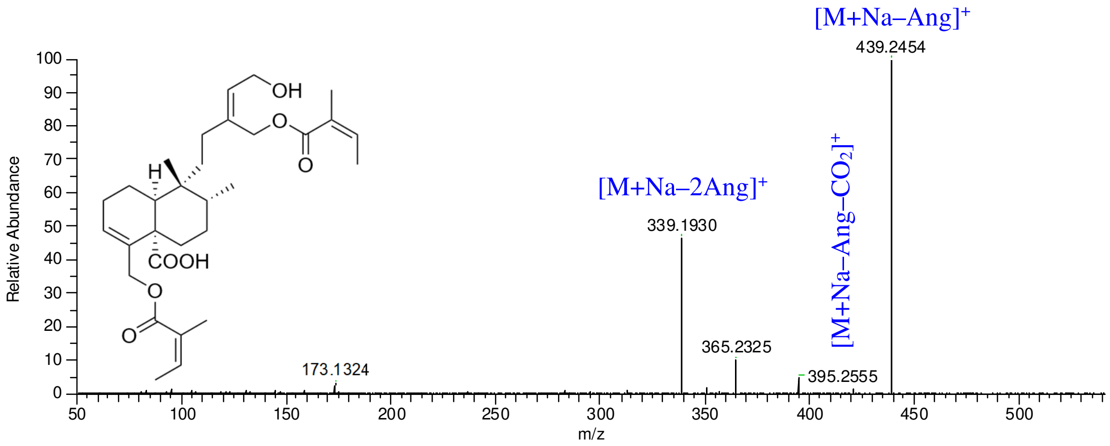

Figure S57. HR-ESI$^+$-MS/MS spectrum of solidagoic acid J (**5**) with a normalized HCD collision energy of 40%. Precursor ion: *m/z* 539.2979 $[M+Na]^+$, $C_{30}H_{44}O_7Na^+$. Ang denotes an angeloyloxy group ($C_5H_8O_2$).

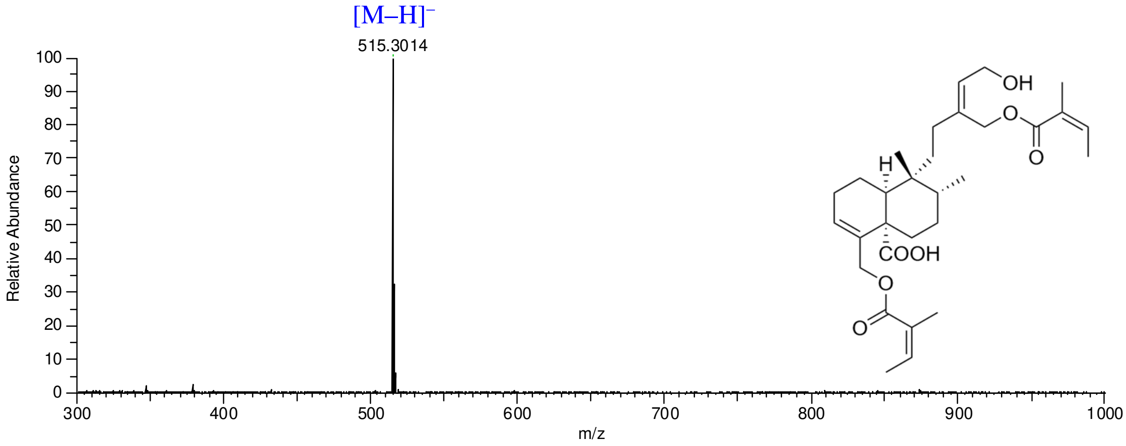

Figure S58. HR-ESI$^-$-MS spectrum of solidagoic acid J (**5**), *m/z* 515.3014 $[M-H]^-$ (calculated for $C_{30}H_{43}O_7^-$, *m/z* 515.3014 $[M-H]^-$, error: 0.0 ppm).

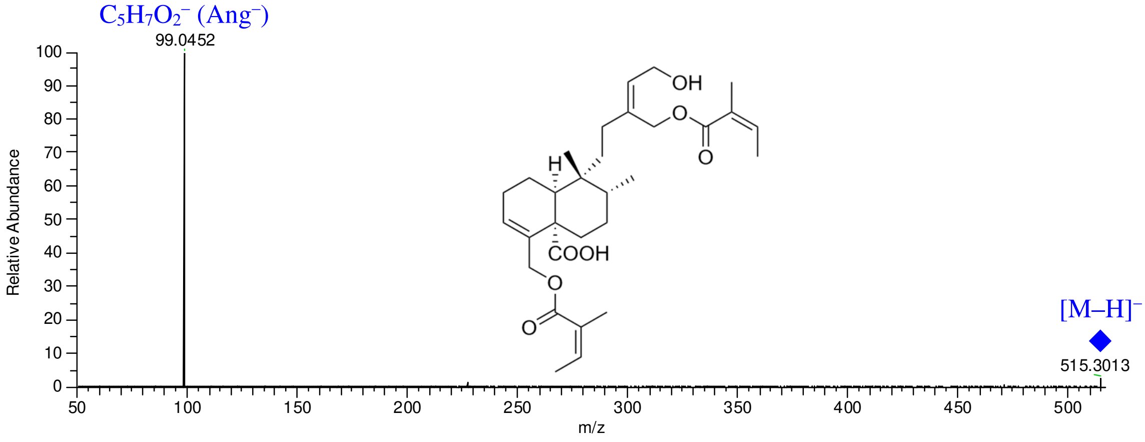

Figure S59. HR-ESI$^-$-MS/MS spectrum of solidagoic acid J (**5**) with a normalized HCD collision energy of 15%. Precursor ion: *m/z* 515.3013 $[M-H]^-$, $C_{30}H_{43}O_7^-$.
